# Association of Human iPSC Gene Signatures and X Chromosome Dosage with Two Distinct Cardiac Differentiation Trajectories

**DOI:** 10.1016/j.stemcr.2019.09.011

**Published:** 2019-10-24

**Authors:** Agnieszka D'Antonio-Chronowska, Margaret K.R. Donovan, William W. Young Greenwald, Jennifer Phuong Nguyen, Kyohei Fujita, Sherin Hashem, Hiroko Matsui, Francesca Soncin, Mana Parast, Michelle C. Ward, Florence Coulet, Erin N. Smith, Eric Adler, Matteo D'Antonio, Kelly A. Frazer

**Affiliations:** 1 Department of Pediatrics, UC San Diego, La Jolla, CA 92093, USA; 2 Bioinformatics and Systems Biology Graduate Program, UC San Diego, La Jolla, CA 92093, USA; 3 Division of Cardiology, Department of Medicine, UC San Diego, La Jolla, CA 92093, USA; 4 Department of Pathology, UC San Diego, La Jolla, CA 92093, USA; 5 Department of Medicine, University of Chicago, Chicago, IL 60637, USA

**Keywords:** iPSC, iPSC-derived cardiovascular progenitor cells, iPSC-derived cardiomyocytes, iPSC-derived epicardium, scRNA-seq, single-cell transcriptomics, X chromosome inactivation, X chromosome erosion, iPSC differentiation

## Abstract

Despite the importance of understanding how variability across induced pluripotent stem cell (iPSC) lines due to non-genetic factors (clone and passage) influences their differentiation outcome, large-scale studies capable of addressing this question have not yet been conducted. Here, we differentiated 191 iPSC lines to generate iPSC-derived cardiovascular progenitor cells (iPSC-CVPCs). We observed cellular heterogeneity across the iPSC-CVPC samples due to varying fractions of two cell types: cardiomyocytes (CMs) and epicardium-derived cells (EPDCs). Comparing the transcriptomes of CM-fated and EPDC-fated iPSCs, we discovered that 91 signature genes and X chromosome dosage differences are associated with these two distinct cardiac developmental trajectories. In an independent set of 39 iPSCs differentiated into CMs, we confirmed that sex and transcriptional differences affect cardiac-fate outcome. Our study provides novel insights into how iPSC transcriptional and X chromosome gene dosage differences influence their response to differentiation stimuli and, hence, cardiac cell fate.

## Highlights

• Cellular heterogeneity across iPSC-CVPCs due to varying fractions of CMs and EPDCs
• iPSC non-genetic factors (clone and passage) associated with cardiac cell fate
• Expression levels of signature genes in iPSCs associated with cardiac lineage fate
• iPSC donor sex plays a role in cardiac lineage fate

## Graphical Abstract

**Figure:**
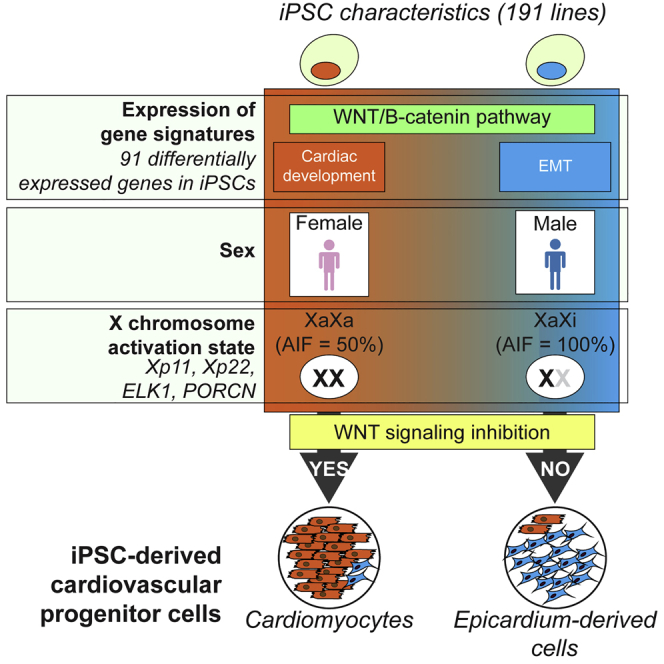

## Teaser

In this article, D'Antonio-Chronowska, Donovan and colleagues differentiated 191 iPSC lines to generate iPSC-derived cardiovascular progenitor cells (iPSC-CVPCs). The iPSC-CVPC samples showed cellular heterogeneity due to varying fractions of cardiomyocytes (CMs) and epicardium-derived cells (EPDCs). Donor sex and expression levels of signature genes in iPSCs played a role in the relative proportions of CMs and EPDCs present in the derived CVPCs.

## Introduction

Variability in human induced pluripotent stem cell (iPSC) lines compromises their utility for regenerative medicine and as a model system for genetic studies. This variability affects iPSC differentiation outcome and, despite using standardized differentiation protocols, results in the generation of samples with cellular heterogeneity (i.e., multiple cell types are present within a given sample and the proportions of cell types vary across samples). Previous large-scale quantitative trait loci studies in iPSCs (DeBoever et al., 2017, Kilpinen et al., 2017) have shown that genetic variation accounts for the majority of expression differences between iPSC lines, but non-genetic (i.e., clonality and passage) factors also contribute to these differences (Panopoulos et al., 2017b). Understanding how non-genetic transcriptional differences between iPSC lines affect their differentiation outcome is necessary to improve the ability to generate cell types of interest.

Well-established small-molecule protocols for generating iPSC-derived cardiovascular progenitor cells (iPSC-CVPCs) (Lian et al., 2013) produce fetal-like cardiomyocytes, which can undergo further specification as cells mature in culture into various cardiac subtypes (atrial, ventricular, or nodal) (Burridge et al., 2014). Based on variable cardiac troponin T (cTnT) staining, the derived samples are known to display cellular heterogeneity (Dubois et al., 2011, Witty et al., 2014), but the origin of the cTnT-negative non-myocyte cells, and whether the same or different non-myocyte cell types are consistently derived alongside cTnT-positive myocytes across samples, have not previously been investigated. The differentiation protocol is dependent on manipulation of WNT signaling, initially through activation of the pathway by GSK3 inhibition, followed by inhibition of the pathway by Porcupine (*PORCN*) inhibition (Mo et al., 2013, Wang et al., 2013). An in-depth analysis of the outcomes of independent differentiations of hundreds of iPSC lines with different genetic backgrounds could provide insights into the origins of the non-myocyte cells, as well as the extent to which non-genetic transcriptional differences between iPSC lines contribute to the iPSC-CVPC cellular heterogeneity.

Here, we used a highly standardized and systematic approach to conduct 232 directed differentiations of 191 iPSC lines into iPSC-CVPCs. We characterized the cellular heterogeneity of the iPSC-CVPC samples and showed that only two distinct cell types were present, cardiomyocytes (CMs) and epicardium-derived cells (EPDCs), which varied in proportion across samples. As differentiation protocols to derive iPSC-CMs and iPSC-EPDCs primarily differ by a step involving WNT inhibition to derive the former but not the latter (Bao et al., 2016), we hypothesized that the observed cellular heterogeneity could result from suboptimal WNT inhibition in subsets of cells across iPSC lines. To test this hypothesis, we analyzed transcriptional differences between iPSC lines that differentiated into CMs and those that differentiated into EPDCs (e.g., iPSCs with a CM fate or EPDC fate) and discovered 91 signature genes associated with these two distinct cardiac differentiation trajectories. These signature genes are involved in differentiation, including the Wnt/β-catenin pathway, muscle differentiation or cardiac-related functions, and the transition of epicardial cells to EPDCs by epithelial-mesenchymal transition (EMT). While the proportion of variance explained by each of the signature genes varied over three orders of magnitude, altogether they captured approximately half of the total variance underlying iPSC fate determination. Additionally, we show that variability in X chromosome gene dosage (X_active_X_active_ versus X_active_X_inactive_ versus XY) across iPSCs plays a role in cardiac-fate determination. The association with X chromosome gene dosage could in part be due to higher expression in CM-fated iPSCs of chrXp11 genes, which encodes *ELK1* and *PORCN*. Transcriptomic analysis of an independent set of 39 iPSCs differentiated to the cardiac lineage using a similar small-molecule protocol (Banovich et al., 2018) confirmed our findings.

## Results

### iPSC-CVPCs Show Cellular Heterogeneity across Samples

**Figure 1:**
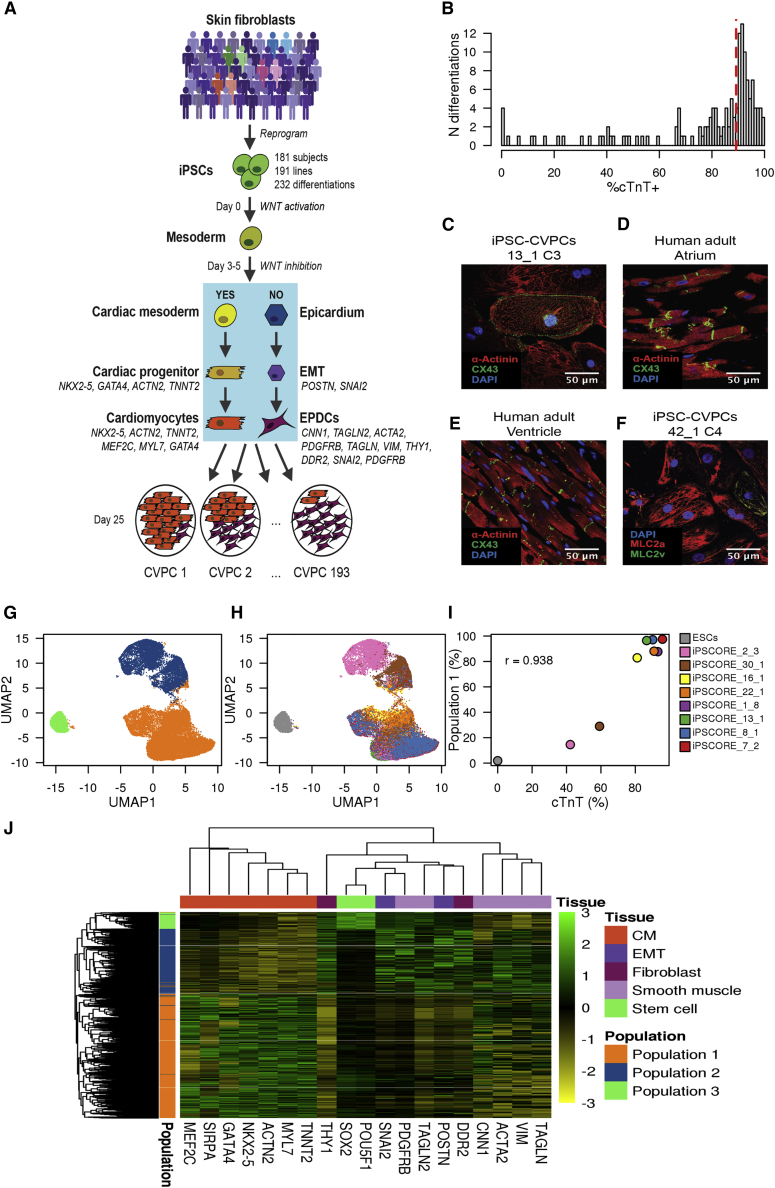
Characterization of Cellular Heterogeneity in iPSC-CVPC Samples (A) Overview of the study design. Skin fibroblasts from 181 subjects were reprogrammed to iPSCs and differentiated to iPSC-CVPCs (191 lines, 232 differentiations). After WNT pathway activation at day 0 and its inactivation by IWP-2 at days 3–5, cells differentiate to CMs if WNT signaling is successfully inhibited. If WNT signaling is not sufficiently inhibited, cells differentiate to EPDCs. Of the 232 differentiations, 193 were completed (day 25), and we observed that different CVPC samples had different proportions of CMs and EPDCs. (B) Distribution of %cTnT. Dashed red line represents the median value. (C–E) Immunofluorescence staining of (C) iPSC-CVPCs, (D) human atrium, and (E) ventricle with markers DAPI, ACTN1, and CX43. (F–H) Immunofluorescence staining of iPSC-CVPCs with markers DAPI, MLC2a^+^ and MLC2v^+^, and MLC2v^+^MLC2a^+^ (F). scRNA-seq UMAP plots showing (G) the presence of three populations: CMs (orange), EPDCs (blue), and ESCs (green), and (H) the distribution of the nine analyzed samples (eight iPSC-CVPC lines and one ESC line) across the three clusters. (I) Scatterplot showing the correlation between the %cTnT and the fraction of cells in population 1 (CMs) for each of the nine samples. (J) Heatmap showing across all 34,905 single cells the expression markers for stem cells, CMs, EMT, fibroblasts, and smooth muscle. See also Figures S1 and S2.

To gain insights into molecular mechanisms that could influence variability in human iPSC differentiation outcome, we employed a highly systematic approach (Figure S1) to differentiate 191 pluripotent lines from 181 iPSCORE individuals (Figure 1A and Table S1A) into iPSC-derived cardiovascular progenitor cells (iPSC-CVPCs). We used a small-molecule cardiac differentiation protocol used to derive cardiomyocytes (Lian et al., 2015) followed on day 15 by lactate selection to obtain pure cardiac cells (Tohyama et al., 2013). In total, we conducted 232 differentiations, of which 193 (83.2%, from 154 lines derived from 144 subjects) were completed, i.e., reached day 25 of differentiation, while 39 (from 37 lines derived from 37 subjects) were terminated prior to day 25 because they did not form a syncytial beating monolayer (Tables S1B and S1C). The completed iPSC-CVPCs at day 25 on average had a high fraction of cells that stained positive for cTnT (%cTnT, median = 89.2%; Figure 1B) and were positive by immunofluorescence for cardiac markers (Figures 1C–1F and S1); however, 15 lines had %cTnT <40%, indicating that despite lactate selection, there was substantial cellular heterogeneity within and across samples.

### Subset of Cells Shows Differential Response to WNT Inhibition during Differentiation

To examine the cellular heterogeneity in the iPSC-CVPCs, we performed single-cell RNA sequencing (scRNA-seq) on eight samples with varying %cTnT values (42.2%–95.8%, Tables S1E and S1F) and combined these data with scRNA-seq from the H9 embryonic stem cell (ESC) line (total of 34,905 cells). We detected three distinct cell populations: (1) population 1, 21,056 cells (60.3%); (2) population 2, 11,044 cells (31.6%); and (3) population 3, 2,805 cells (8.1%, Figures 1G and S1; Table S1G). While populations 1 and 2 comprised the eight iPSC-derived samples, population 3 almost exclusively included ESCs (97.7% of the 2,870 ESCs, Figures 1H and S2). The relative proportions of cells that each of the iPSC-CVPC samples contributed to population 1 versus population 2 was strongly correlated with its %cTnT value (r = 0.938, p = 1.89 × 10^−4^, t test; Figure 1I), suggesting that population 1 was cardiomyocytes (CMs).

As CMs and epicardium lineage cells could both survive lactate purification (Iyer et al., 2015, Tohyama et al., 2013), we investigated whether the non-myocyte cells composing population 2 were iPSC-EPDCs. We examined the expression levels of 17 marker genes (Figure 1J) specific for either CMs or EPDCs (including smooth muscle, fibroblasts, and genes involved in EMT) and two marker genes for stem cells. Consistent with having a high number of cTnT-positive cells, population 1 expressed high levels of CM-specific genes while population 2 expressed high levels of EPDC-specific genes, and population 3 expressed high levels of the stem cell markers *POU5F1* and *SOX2* (Figure S2). Of note, *TNNT2* was expressed in some of the cells in population 2, which is consistent with the strong, but not absolute correlation between %cTnT value and fraction of population 1 (Figure 1I), and previous studies showing that some EPDCs express *TNNT2* (Witty et al., 2014). These results show that the small-molecule differentiation protocol followed by lactate purification resulted in the absence of undifferentiated cells at day 25 and in the derivation of two distinct cell populations, one of which expresses high levels of CM markers, including *TNNT2*, *NKX2-5*, and *MEF2C* (population 1), and the other which expresses EPDC markers, including *SNAI2*, *DDR2*, *VIM*, and *ACTA2* (population 2). Of note, the protocols for generating iPSC-derived cardiomyocytes (iPSC-CMs) and iPSC-EPDCs both involve activating the WNT signaling pathway (Bao et al., 2016, Iyer et al., 2015) and have a shared intermediate mesoderm progenitor, but subsequent WNT inhibition directs differentiating cells to iPSC-CMs and endogenous levels of WNT signaling direct differentiating cells to iPSC-EPDCs (Witty et al., 2014) (Figure 1A). Therefore, our results suggest that iPSC-CVPC cellular heterogeneity results from suboptimal WNT inhibition in a subset of cells during differentiation, which then give rise to EPDCs.

### iPSC-CVPCs Are Composed of Immature CMs and EPDCs

**Figure 2:**
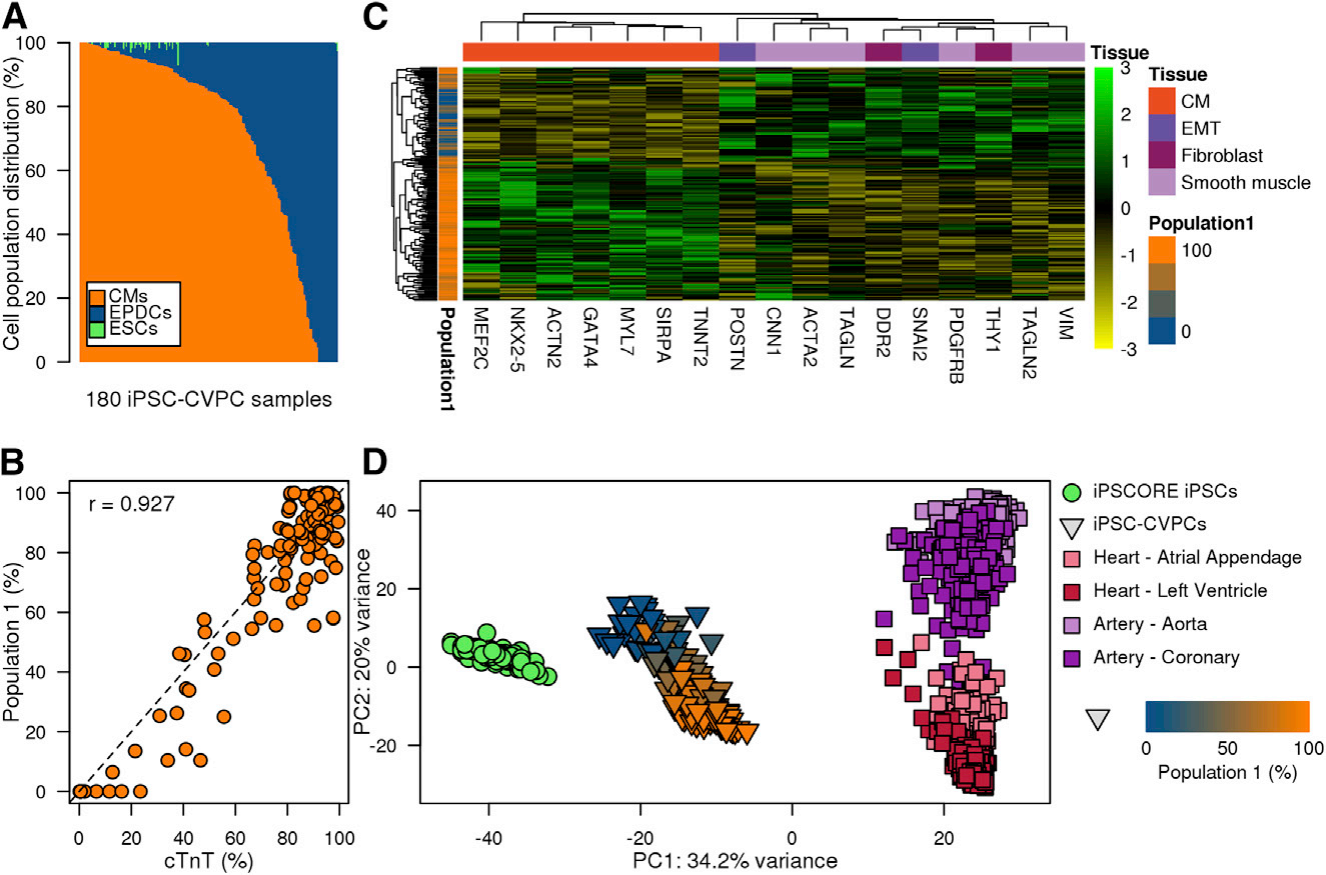
Transcriptomic Features of 180 iPSC-CVPC Samples (A) Relative distributions of cell populations estimated using CIBERSORT across 180 iPSC-CVPC samples. (B) Scatterplot showing the correlation between %cTnT (x axis) and the fraction of population 1 in the iPSC-CVPCs calculated using CIBERSORT (y axis). (C) Heatmap showing the expression levels of CM and EPDC marker genes (Figure 1J) in 180 iPSC-CVPC samples. Samples are colored based on their fraction of population 1. (D) PCA of the 1,000 genes with highest variability from 184 iPSC samples, 180 iPSC-CVPCs (triangles colored according to their percentage of population 1), and samples from GTEx (squares—left ventricle, right ventricle, coronary artery, and aorta).

To estimate the relative abundances of CM and EPDC cells across our collection of iPSC-CVPC samples, we selected the top 50 significantly overexpressed genes in each of the three scRNA-seq populations (150 genes in total, p < 10^−13^, edgeR, Table S2), obtained their expression levels in bulk RNA-seq from 180 iPSC-CVPCs, and inputted these values into CIBERSORT (Newman et al., 2015). We observed that the proportions of each cell type varied across the samples, although the iPSC-CVPCs tended to have a greater fraction of CMs (84.8% ± 31.8%, Figure 2A) than EPDCs (14.7% ± 32.0%), and essentially no stem cells (0% ± 0.8%). Due to lactate selection, the small number (67) of cells predicted to be ESCs may represent a distinct differentiated cell type that is more similar to stem cells than either CMs or EPDCs. The estimated fraction of CMs and EPDCs in the iPSC-CVPCs was highly correlated with %cTnT values (r = 0.927, p ≈ 0, t test; Figure 2B), similar to that observed in the analysis of the scRNA-seq data (Figure 1J). Finally, we showed that the iPSC-CVPCs with high estimated CM or EPDC cellular fractions, respectively, showed higher expression of CM markers (*MEF2C*, *NKX2-5*, and *ACTN2*) and EPDC markers (*ACTA2*, *TAGLN*, *DDR2*, and *SNAI2*, Figure 2C). These results indicate that cellular heterogeneity across iPSC-CVPC samples largely reflects different proportions of CMs and EPDCs.

To characterize the similarities between the iPSC-CVPC transcriptomes and those of adult heart and artery samples, we performed a principal-component analysis (PCA) using the transcriptomes of 184 iPSCORE iPSCs, 180 iPSC-CVPCs, and the 1,072 GTEx samples, including left ventricle, atrial appendage, coronary artery, and aorta (GTEx Consortium et al., 2017). We found that principal component 1 (PC1) showed that iPSC-CVPCs correspond to an intermediate state between the iPSCs and adult samples, suggesting that the derived CMs and EPDCs are similar to immature cardiac cells (Figure 2D). PC2 divided the samples based on cardiac lineage, namely the myocardium (left ventricles and atrial appendages) and epicardium (coronaries and aorta) (Perez-Pomares et al., 2016). This analysis shows that derived iPSC-CMs and iPSC-EPDCs lie on different cardiac developmental trajectories, with the CMs corresponding to immature myocardium and the EPDCs to immature epicardium.

### iPSC Expression Signatures Affect Cardiac-Fate Differentiation

**Figure 3:**
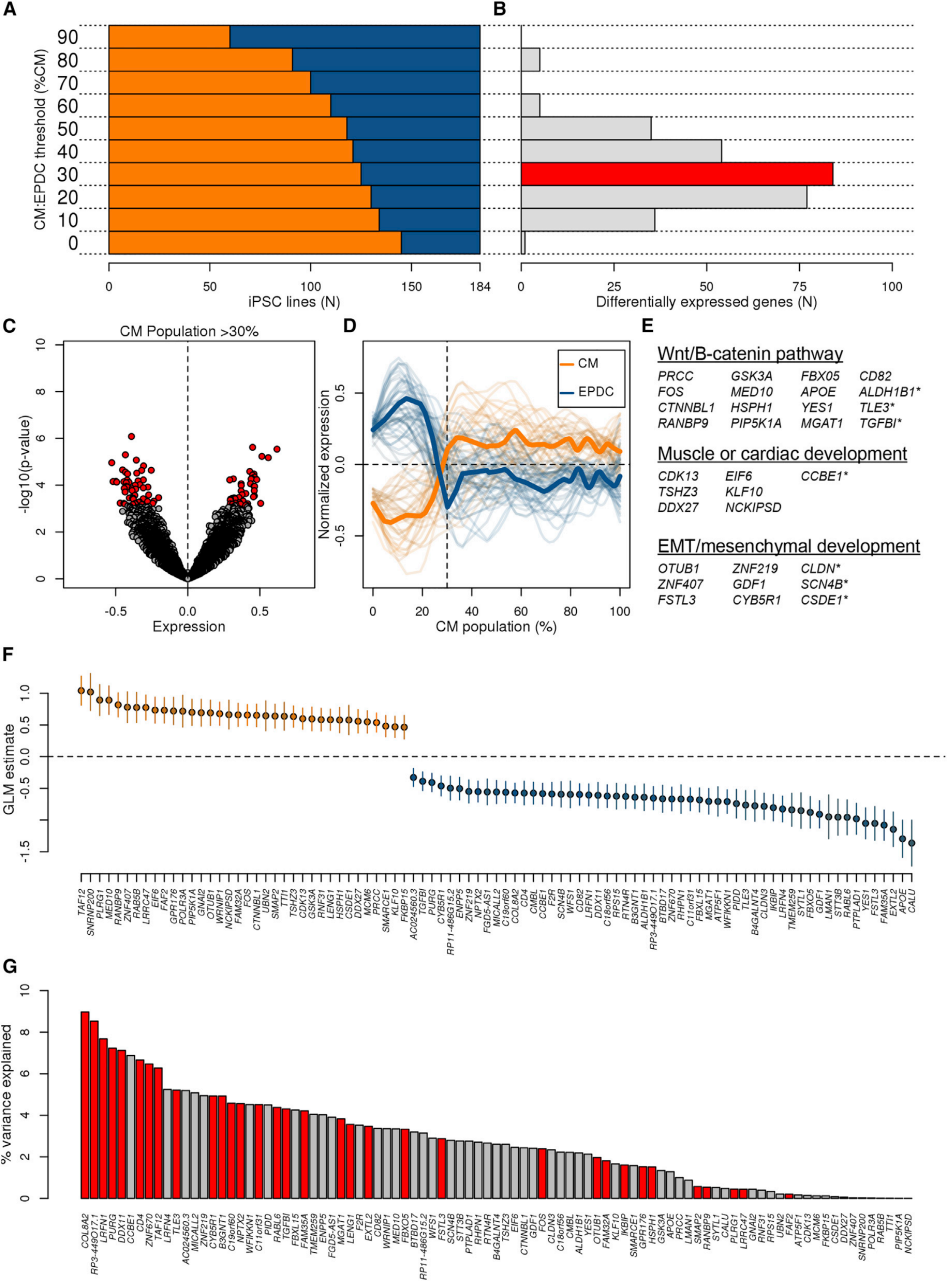
iPSC Gene Signatures Associated with Cardiac Differentiation Fate (A) Testing of ten CM/EPDC ratios (0:100 to 90:10, with 10% increments) to determine the optimal threshold for defining an iPSC as CM-fated or EPDC-fated. For each threshold, the number of iPSC lines defined as CM-fated (orange) or EPDC-fated (blue) is shown. (B) At the same thresholds indicated in (A), shown are the numbers of differentially expressed autosomal genes between the iPSC lines defined as CM-fated and EPDC-fated. The 30:70 threshold has the maximum number of differentially expressed genes. (C) Volcano plot showing mean difference in expression levels for all autosomal genes between CM-fated iPSC lines and EPDC-fated iPSC lines (x axis) and p value (y axis, t test). A positive difference indicates overexpression in CM-fated iPSCs, whereas a negative difference indicates overexpression in EPDC-fated iPSCs. Significant genes are indicated in red. (D) Expression levels of the 91 signature genes in iPSCs as a function of the %CM population in their corresponding iPSC-CVPC samples. Thick lines represent the average for 36 genes overexpressed in CM-fated iPSCs (orange) and for 55 genes overexpressed in EPDC-fated iPSCs (blue). (E) WNT/β-catenin pathway, muscle/cardiac related, or EMT/mesenchymal development signature genes (those differentially expressed with nominal p values [p < 0.0015] indicated with an asterisk). (F) GLM estimate (%CM population/expression) calculated for each signature gene. Mean and 95% confidence interval are shown. (G) Bar plot showing the percentage of variability in iPSC fate that is explained by each of the 91 signature genes. Bars highlighted in red show the 35 signature genes identified by L1 normalization that independently contributed to variance. Due to the fact that the 91 genes do not have independent expression, the total sum of the percent variance explained is >1. See also Figures S3–S5.

Although all iPSCORE iPSCs have previously been shown to be pluripotent (Panopoulos et al., 2017a), we sought to determine whether transcriptomic differences existed between the iPSC lines that derived CVPCs containing CMs versus those that gave rise to EPDCs (Figure 3A). Given that all 180 iPSC-CVPCs contain both CMs and EPDCs but at different ratios, we initially had to determine the optimal CM/EPDC ratio to group the iPSC lines into those that were CM-fated and those that were EPDC-fated. Thresholds for 193 iPSC-CVPCs that completed differentiation (harvested on day 25) were defined by the ratio of CM/EPDC estimates from CIBERSORT (estimated %CM/estimated %EPDC), while the 39 iPSC-CVPC differentiations terminated prior to day 25 for not forming a beating syncytium were assigned a CM/EPDC ratio of 0:100 (0% CM/100% EPDC). We tested ten different CM/EPDC ratios and found 116 autosomal genes that were differentially expressed at one or more of these ratios (Storey q value <0.1, t test; Figures 3A and 3B; Table S3A). We observed that the maximum number of the 116 genes (84, 72.5%) was differentially expressed at the 30:70 (CM/EPDC) threshold and 55 of them (47.4%) had their strongest p value at this ratio (Figure S3). For this reason, we determined that the 30:70 threshold was optimal, and grouped the iPSCs into 125 that were CM-fated (produced ≥30% CMs) and 59 that were EPDC-fated (produced >70% EPDCs; Figures 3B and S4; Table S3B).

Of the 84 autosomal differentially expressed genes at the 30:70 (CM/EPDC) threshold, 35 were overexpressed in the CM-fated iPSC lines and 49 were overexpressed in the EPDC-fated iPSCs (Figures 3B–3D). These genes have functions associated with three differentiation signatures: (1) Wnt/β-catenin pathway (13 genes); (2) muscle and/or cardiac differentiation (six genes); and (3) EMT and/or mesenchymal tissue development (six genes; Figure 3E and Table S3C). We noted that seven borderline significant autosomal genes were also involved in one of the three represented signatures, and therefore added them to the final list of differentially expressed genes. We investigated the associations between the expression levels of the final list of 91 signature genes in the 184 iPSCs and the fraction of CMs in the resulting iPSC-CVPCs using linear regression, and found significant associations for all genes (Figure 3F and Table S3D). These results show that, independently of the 30:70 (CM/EPDC) threshold used in the initial differential expression analysis, the expression levels of these signature genes in the 184 iPSCs were significantly associated with differentiation outcome (e.g., CM or EPDC fate).

### Signature Genes Capture a Large Fraction of the Variance Underlying iPSC Fate Outcome

While the signature genes likely affected cardiac-fate determination, we did not expect each gene to contribute equally. To explore the impact of each gene individually on differentiation outcome, we calculated how much the 91 genes explained the variability underlying iPSC cell fate. To quantify the percent of variance explained by each gene (R^2^), we fit a generalized linear regression model with a logit link function to each gene individually. We found that the percentage of variance explained by each individual gene varied over three orders of magnitude (1.73 × 10^−3^ < R^2^< 8.97%; Figure 3G).

We next asked how these signature genes altogether captured variability in differentiation fate. As several of the signature genes had correlated expression levels (Figure S4), to reduce overfitting in the regression analysis we included an L1 norm penalty (i.e., LASSO regression) and used 10-fold cross-validation. We identified 35 genes that independently contributed to variance and whose expression levels collectively explained more than half of the variability in differentiation outcome across iPSC lines (average R^2^ from the 10-fold cross-validation = 0.512). Together these data show that, while the proportion of variance explained by each of the signature genes varied widely, altogether they captured approximately half of the total variance underlying differential iPSC fate outcome.

### Inherited Genetic Variation Does Not Influence Differentiation Outcome

We investigated whether genetic variation associated with the expression of any of the signature genes contributed to the differentiation outcome of iPSCs. We assessed the genotypes of 8,620,159 variants in each iPSC line and performed a genome-wide association study (GWAS) to investigate the association between genotype and the fraction of CMs in the corresponding iPSC-CVPCs. We found that none of these variants was associated with differentiation outcome at genome-wide significance (p < 5 × 10^−8^; Table S3E and Figure S5). To further examine the association between genetic background and differentiation outcome, we tested whether differentiations of different iPSC clones from the same individual, and from members of the same twin pair, were more likely to yield similar outcomes compared with differentiations of iPSC clones from individuals with different genetic backgrounds, and observed similar distributions (Figure S5). While our power to perform a GWAS was limited, this analysis shows that the genetic background did not contribute to the variance underlying iPSC differentiation outcome, indicating that non-genetic (i.e., clonality and passage) factors played a role in determining whether an iPSC line differentiated to CMs or EPDCs.

### GSEA Implicates ELK1 Targets and Genes on the X Chromosome

**Figure 4:**
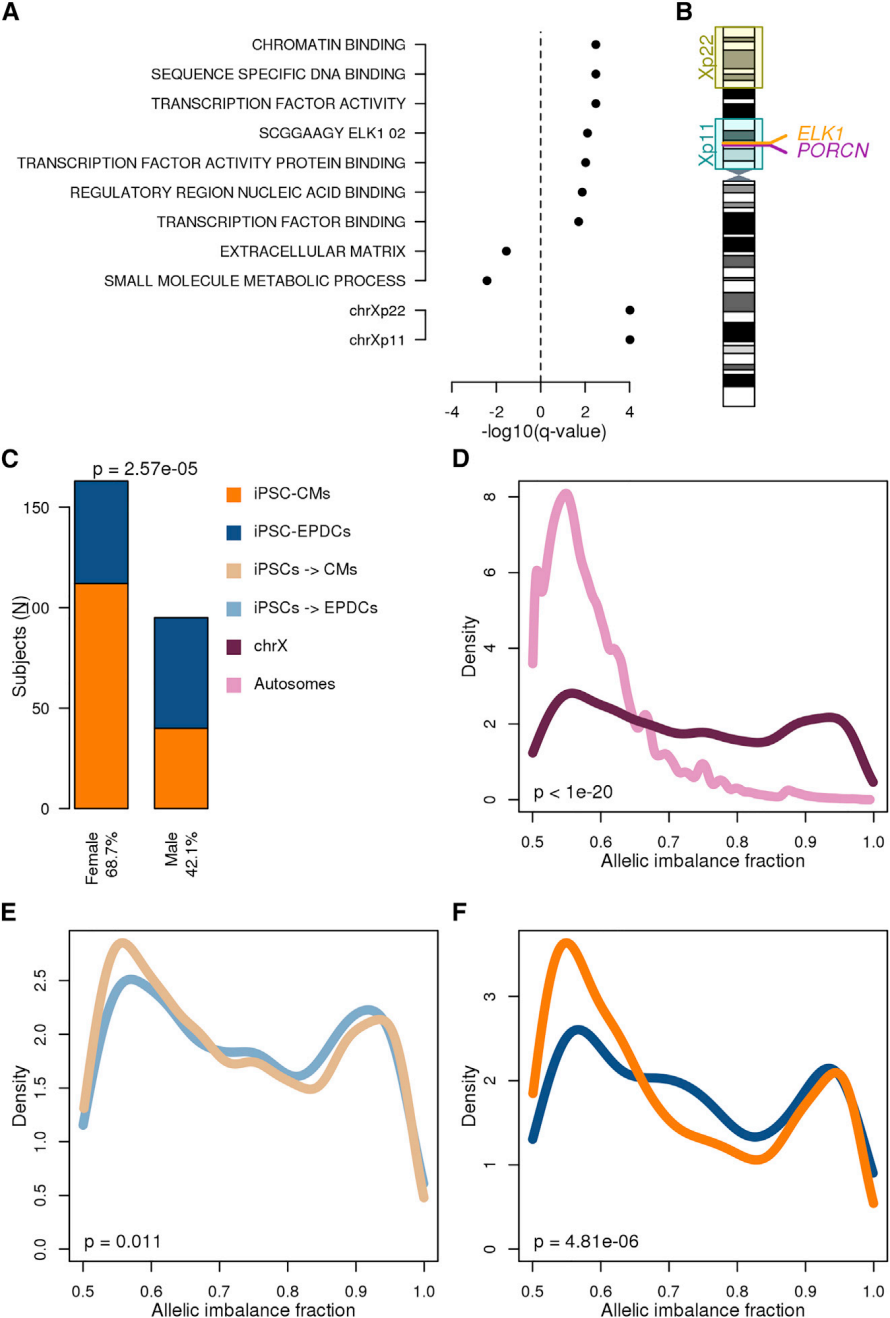
X Chromosome Gene Dosage Plays a Role in Cardiac Differentiation Fate (A) GSEA results. For each gene set, −log_10_(q value) is shown. Positive values correspond to gene sets enriched in CM-fated iPSCs, whereas negative values correspond to EPDC-fated iPSCs. For autosomes all iPSCs were included (top), for the chromosome X only the 113 female iPSCs were analyzed (bottom). Storey q value was used to adjust for multiple testing hypothesis; q values <0.05 were considered significant. (B) Cartoon showing the positions of differentially expressed loci on chromosome X and of *ELK1* and *PORCN*. (C–F) Bar plot (C) showing the associations between sex and differentiation outcome (orange: iPSC-CVPC samples with CM fraction >30%; blue: with EPDC fraction >70%). p values were calculated using *Z* test. Density plots showing the differences in allelic imbalance fraction between: (D) autosomal genes (pink) and chrX genes outside of the pseudoautosomal region (maroon) in female iPSCs; (E) chrX genes in female CM-fated (light orange) and EPDC-fated (light blue) iPSCs; (F) chrX genes in female day 25 iPSC-CVPC samples with CM fraction >30% (orange) and EPDC fraction >70% (blue). p values in (D) to (F) were calculated using the Mann-Whitney U test. See also Figure S6.

To understand whether the transcriptomic differences between CM-fated and EPDC-fated iPSCs were associated with alterations in specific pathways or cellular function, we performed a gene-set enrichment analysis (GSEA) on 9,808 MSigDB gene sets (Subramanian et al., 2005) using the 15,228 expressed autosomal genes in the 184 iPSCs. We identified 22 gene sets that were significantly associated with iPSC fate, including enrichment in the 59 EPDC-fated iPSCs for extracellular matrix (Figure 4A and Table S4A) and in the 125 CM-fated iPSCs for transcription factor activity and ELK1 targets. To capture gene sets associated with expression differences on the X chromosome, we performed differential expression and GSEA on 113 female iPSC lines (87 CM-fated and 26 EPDC-fated). The two most significant gene sets were loci located within chrXp11 and chrXp22 (Figure 4B). Notably, the chrXp11 locus encodes both *ELK1* and *PORCN*, whose protein product (Porcupine) is targeted for WNT inhibition in CM differentiation protocols but not EPDC differentiation protocols (Mo et al., 2013, Wang et al., 2013) (Figure 4B). The chrXp22 locus includes the majority of genes (52/99, 52.5%) that are known to escape chromosome X inactivation (Tukiainen et al., 2017), and thus may potentially have varying X-linked gene dosage across female iPSCs. Overall, GSEA shows that genes differentially expressed between CM-fated and EPDC-fated iPSCs are involved in a variety of pathways, including ELK targets, and are potentially associated with the X chromosome activation status.

### Sex Is Associated with iPSC Differentiation Outcome

To identify other iPSC factors potentially associated with differentiation outcome, we examined three characteristics of the 181 subjects in our study (sex, ethnicity, and age) and passage of the iPSCs at day 0. Analyzing the 125 CM-fated and 59 EPDC-fated iPSC lines with a general linear model, we found no association between differentiation outcome and age or ethnicity (p > 0.8; generalized linear model [GLM], *Z* test; Figure S6 and Table S4B), but observed a significant association with sex (p = 2.57 × 10^−5^, GLM, *Z* test; Figure 4C) and a trend for iPSC passage at day 0 (p = 0.069, GLM, *Z* test; Figure S6). These data suggest that iPSCs derived from female subjects and iPSCs with higher passages at day 0 had an increased predisposition for the CM fate. Furthermore, considering only the 191 completed differentiations (day-25 iPSC-CVPC samples), we found that iPSC-CVPC samples derived from female subjects compared with those derived from males had significantly higher %cTnT values (mean = 83.0% and 77.7%, respectively, for females and males; p = 6.0 × 10^−4^, Mann-Whitney U test; Figure S6) and a higher fraction of CMs (p = 6.46 × 10^−4^, Mann-Whitney U test). These results indicate that iPSCs derived from female subjects and, to a lesser extent, iPSCs that have spent more time in cell culture have a greater inherent predisposition to differentiate toward the CM lineage.

### Female iPSCs with X Chromosome Reactivation Associated with CM Fate

Given the observation that female iPSCs have a greater potential to differentiate to CMs and that differential expression of chrXp11 genes were associated with differentiation outcome, we asked whether variation in X chromosome inactivation (Xi) and activation (Xa) state across female iPSC lines was associated with CM or EPDC fate. Using RNA-seq data generated from the 113 female iPSCs, we evaluated allele-specific effects (ASE) of X chromosome and autosomal genes (Table S4C). We defined the strength of ASE for each gene as the fraction of RNA transcripts that were estimated to originate from the allele with higher expression (hereafter referred to as “allelic imbalance fraction” [AIF]). We observed that AIF in autosomal genes was close to 0.5, indicating that both alleles were equally expressed (Figure 4D), while AIF on the X chromosome in iPSCs tended to be bimodal, with some genes showing monoallelic expression (AIF ∼1.0; XaXi) and others showing biallelic expression (AIF ∼0.5; XaXa). We observed that AIF was less in the 87 CM-fated female iPSCs compared with the 26 EPDC-fated female iPSCs (p = 0.011, Mann-Whitney U test; Figure 4E) and that this difference in AIF became even more pronounced in the corresponding derived iPSC-CVPC samples (p = 4.81 × 10^−6^, Mann-Whitney U test; Figures 4F and S7). These findings show that in iPSCs, differential chromosome XaXi status as well as altered gene expression in chrXp22 and chrXp11 contributes to differences in cardiac-fate differentiation outcome.

Since we observed differences in X chromosome reactivation state between CM-fated and EPDC-fated female iPSCs, we next asked whether the two GSEA X chromosome-associated intervals (chrXp22 and chrXp11; Figure 4A) showed corresponding allelic imbalance trends. We plotted AIF differences, whereby a positive AIF difference indicates X chromosome reactivation in the 26 EPDC-fated iPSCs and a negative effect in the 87 CM-fated iPSCs (Figure S6). We observed that distinct regions across the X chromosome were differentially eroded in the EPDC-fated versus CM-fated iPSCs. In particular, chrXp22 showed X reactivation in CM-fated iPSCs (p = 6.31 × 10^−3^, Mann-Whitney U test), with both escape (p = 0.020, Mann-Whitney U test) and non-escape genes (p = 0.023, Mann-Whitney U test) showing evidence of reactivation (Figure S6). As chrXp22 contains more than half of the escape genes on the X chromosome, this observation confirms that increased X reactivation in CM-fated iPSCs results in increased expression of both escape and non-escape genes. As GSEA identified genes on chrXp11 to be overexpressed in CM-fated iPSCs, the lack of X reactivation in this interval (p = 0.28, Mann-Whitney U test) suggests that alternative regulatory mechanisms may also alter gene-expression levels on the X chromosome. Overall, these results suggest that differential X chromosome reactivation as well as other mechanisms underlying altered regulation of X chromosome and autosomal genes contribute to iPSC cardiac lineage fate determination.

### Independent iPSC-CM Derivation Study Validates Findings

**Figure 5:**
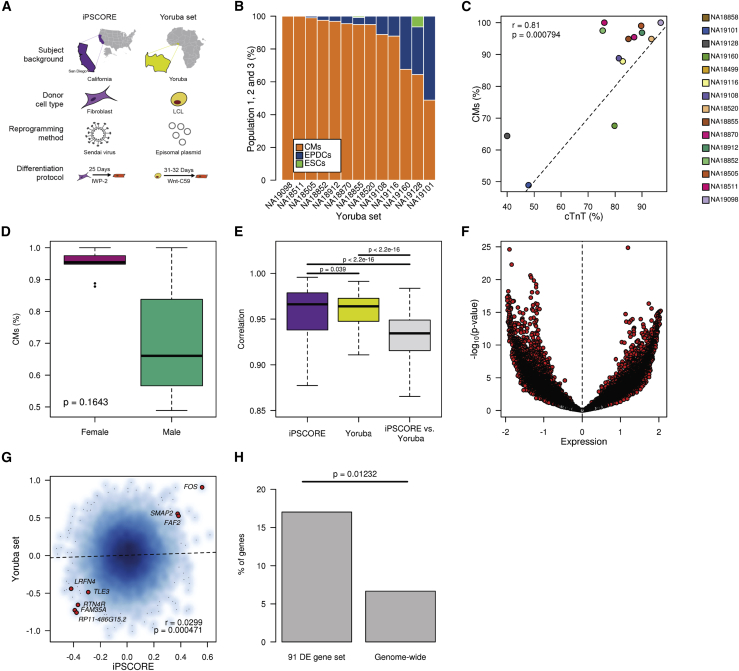
Validation of Association between iPSC Gene Signatures, Sex, and Differentiation Outcome (A) Schematic depicting differences between the iPSCORE and Yoruba iPSC samples. (B) Estimated fractions of CMs and EPDCs for 13 Yoruba iPSC-CM samples from RNA-seq using CIBERSORT (two iPSC-CMs did not have RNA-seq). (C) Scatterplot showing the correlation between %cTnT and the fraction of cells in population 1 for 13 Yoruba iPSC-CM samples. (D) Box plots showing the distribution of estimated fraction of cells in population 1 in females and males. (E) Box plots showing correlation of gene expression in all 184 iPSCORE iPSCs with RNA-seq (purple), 34 Yoruba iPSCs with RNA-seq used for differentiation, and the pairwise comparison of the Yoruba iPSCs against the iPSCORE iPSCs (gray). (F) Volcano plot showing mean difference in expression levels for all autosomal genes between 14 Yoruba iPSC lines that were successfully differentiated and 125 iPSCORE iPSC-CM-fated lines and p value (y axis, t test). Significant genes are indicated in red. (G) Smooth color density scatterplot showing gene-expression differences between iPSCs with different fates in 184 iPSCORE iPSCs to the expression differences between iPSCs with different outcomes in Yoruba iPSCs (14 successful versus 20 terminated) (y axis). A positive difference indicates shared overexpression of genes between CM-fated iPSC in iPSCORE and successfully differentiated iPSC in the Yoruba set, whereas a negative difference indicates shared overexpression of genes between EPDC-fated iPSC in iPSCORE and terminated iPSC in the Yoruba set. Of the 91 signature genes that were differentially expressed in the iPSCORE iPSCs based on cell fate, eight had nominally significant expression differences in the same direction in the Yoruba iPSC set (shown in red). (H) Bar plot showing that the eight iPSCORE differentially expressed genes in (G) with nominal significant expression differences in the same direction (e.g., overexpressed or down regulated) in the Yoruba iPSCs are greater than random expectation. See also Figure S7.

To assess the generalizability of our findings, we examined an independent collection of 39 iPSCs (Banovich et al., 2018) reprogrammed using an episomal plasmid from Yoruba lymphoblastoid cell lines (Figure 5A and Table S5). Differentiation of these lines resulted in the successful derivation of 13 iPSC-CMs (%cTnT range at day 32: 40–96.9), whereas 24 were terminated on or before day 10 due to the fact that they did not form a beating syncytium. To examine whether the successfully derived Yoruba iPSC-CMs showed the presence of EPDCs, we used RNA-seq data and CIBERSORT to estimate cellular compositions and observed variable relative distributions of CM and EPDC populations (Figure 5B). Consistent with our iPSCORE iPSC-CVPC samples, the estimated CM population fractions were significantly correlated with %cTnT values (r = 0.81, p = 7.94 × 10^−4^, t test; Figure 5C). To understand whether the CMs and EPDCs appear at the same time during differentiation, we analyzed data generated the from the Yoruba lines at four time points (Strober et al., 2019) and observed that both cardiac lineages are typically present by day 5 and that the ratio of these two cardiac cell types remains relatively stable past day 10 (Figure S7). Finally, Yoruba iPSC-CMs derived from females tended to have an increased percentage of CMs compared with those derived from males (Figure 5D). These observations show that the Yoruba iPSCs and derived cardiac cells could be used to investigate the generalizability of the associations that we had observed between transcriptomic differences in iPSCs and cardiac-fate differentiation outcome.

As several factors (Figure 5A) were different between the iPSCORE iPSC and Yoruba iPSC sets (i.e., different reprogramming method, genetic backgrounds, and donor cell types), we expected that there would be significant differences between their transcriptional profiles. We initially analyzed how correlated gene expression was: (1) within iPSCORE iPSCs; (2) within Yoruba iPSCs; and (3) between all pairwise comparisons of the iPSCs in these two different collections (Figure 5E). We observed high correlations of gene expression across iPSCs within each collection; however, the correlation between samples from different studies was significantly decreased, indicating that the two sets have significant genome-wide gene-expression differences. We next examined differential gene expression between the CM-fated iPSCORE iPSC and Yoruba iPSCs that successfully differentiated into iPSC-CMs (Figure 5F), and observed that the majority of genes (69.6% with q value <0.10) were significantly differentially expressed between the two iPSC sets. These results show that there are strong batch effects on gene expression between the iPSCORE and Yoruba iPSC lines.

We investigated whether, despite the strong batch effects on gene expression between iPSCORE and Yoruba iPSCs, we could detect inherent transcriptional differences affecting cardiac-fate determination that were shared between the iPSC sets. Given the relatively small size of the Yoruba study, there was insufficient power to detect transcriptional differences between the lines with different differentiation outcomes (successfully completed versus terminated). Therefore, for each gene, we compared the mean expression differences between iPSCs with different cardiac-fate outcomes in iPSCORE (CM fate minus EPDC fate) to the expression differences between iPSCs with different differentiation outcomes in the Yoruba set (successfully completed minus terminated; Figure 5G). We observed a small but significant correlation (r = 0.0299, p = 4.71 × 10^−4^, t test) between genes that were differentially expressed in the iPSCORE iPSCs and those that were differentially expressed in the Yoruba iPSCs. Furthermore, we specifically examined the 91 signature genes significantly associated with iPSCORE iPSC cardiac-fate outcome and found eight with nominally significant expression differences in the same direction (e.g., overexpressed or downregulated) in the two sets of iPSCs (Figure 5G), which is 2.5 times more than random expectation (p = 0.012, Fisher's exact test; Figure 5H). These data suggest that the iPSCORE iPSCs and Yoruba iPSCs shared transcriptional differences that affected cardiac-fate differentiation outcome.

## Discussion

While previous directed cardiac differentiation studies have observed the emergence of both cardiomyocytes and a non-contractile cell population, the origin of these non-contractile cells, and whether the same or different non-myocyte cell types are present across iPSC-CVPC samples, has not previously been addressed. We showed that two distinct cell types were present in 154 iPSC-CVPC samples derived from iPSCs in iPSCORE. One of the derived cell types were CMs, characterized by high expression levels of cardiac-specific genes, and the other derived cell type was EPDCs, characterized by high expression of marker genes for EMT, smooth muscle, and fibroblasts. We found the same two cardiac cell types present in iPSC-CMs derived from an independent collection of 39 Yoruba iPSCs, both of which were typically present by day 5, and their ratios remained relatively stable past day 10 (Banovich et al., 2018, Strober et al., 2019). A recent study showed that adding human ESC-derived epicardial cells to cardiomyocyte grafts *in vivo* improves transplantation efficacy, as it increases contractility, myofibril structure, and calcium handling and decreases tissue stiffness (Bargehr et al., 2019). Our findings suggest that the generation of EPDCs during iPSC-CM differentiation may enhance the structure of the derived CMs and that to efficiently use iPSC-CVPCs in a clinical setting, future studies may need to optimize the relative proportions of CMs and EPDCs that maximize their transplantation efficiency.

**Figure 6:**
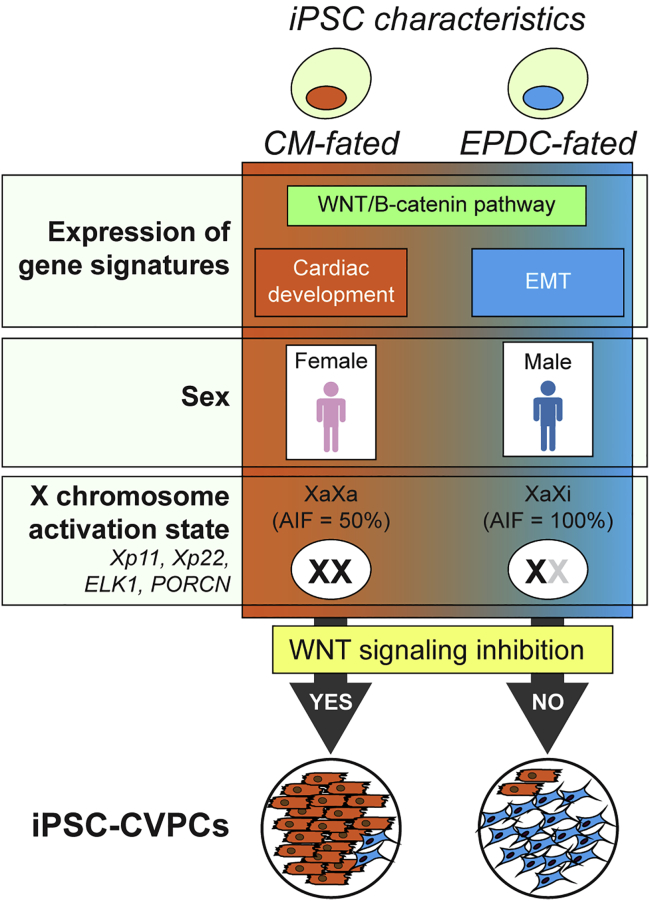
iPSC Characteristics that Influence Their Cardiac-Fate Determination Cartoon showing iPSC characteristics that influence their cardiac-fate determination, including: (1) the expression levels of 91 genes grouped into three gene signature classes (WNT/B-catenin pathway, cardiac development genes, and genes involved in EMT); (2) sex: female iPSCs are more likely to differentiate to CMs than males; and (3) X chromosome activation state: female iPSCs that have activated both X chromosomes (XaXa) are more likely to differentiate to CMs.

The scale of our study, 232 attempted differentiations of 191 iPSC lines into the cardiac lineage, provided the power to develop a framework to identify non-genetic transcriptional differences in iPSCs that influence their cardiac differentiation outcome. To minimize the factors that might influence differentiation outcome, such as the optimal cell confluence at which to start differentiation, we attempted to standardize all steps in the differentiation protocol in order to remove subjective decisions and diminish experimental differences between samples. We identified 91 signature genes whose differential expression was associated with differentiation outcome and showed that many of these genes are involved in cardiac development, including the Wnt/β-catenin pathway, muscle differentiation or cardiac-related functions, and the transition of epicardial cells to EPDCs by EMT (Figure 6). Many of the transcriptomic differences between iPSCORE iPSCs with CM fates versus those with EPDC fates may be due to aberrant epigenetic landscapes resulting from a combination of the reprogramming method (Sendai virus) and cell of origin (fibroblasts). However, given that the Yoruba iPSCs were reprogrammed using a different method (episomal plasmid) and cell of origin (lymphoblastoid cell lines [LCLs]) and yet the iPSCORE and Yoruba iPSCs shared gene-expression differences associated with cardiac lineage outcome, it is likely that our findings will likely be generalizable to other collections of iPSCs. We hypothesize that the signature genes associated with cardiac lineage outcome will vary across iPSC collections and depend on the reprogramming method and cell type of origin, but will largely be involved in the same pathways identified in this study.

We observed that variability across iPSCs on X chromosome gene dosage (XaXa versus XaXi versus XY) played a role in cardiac lineage fate (Figure 6). While human iPSCs are known to have only partial XaXa (Barakat et al., 2015, Kim et al., 2014), we identified two loci (chrXp11 and chrXp22) encoding genes whose expression levels are associated with two distinct cardiac differentiation trajectories (CMs versus EPDCs). The higher expression of chrXp11 genes in CM-fated iPSCs may at least in part be due to fact that *ELK1* and *PORCN* are both encoded in this interval, as the protein product of *PORCN* (Porcupine) is inhibited by IWP-2 during CM differentiation (Mo et al., 2013) but not during EPDC differentiation (Bao et al., 2016, Iyer et al., 2015, Witty et al., 2014) (some EPDC protocols inhibit Porcupine but then reactivate the WNT pathway at a later time point [Guadix et al., 2017, Paik and Wu, 2017, Zhao et al., 2017]). Furthermore, we found that ELK1 targets are overexpressed in CM-fated iPSCs, which is consistent with previous studies showing that knockdown of *ELK1* in immortalized human bronchial epithelial cells, small airway epithelial cells, and luminal breast cancer cell line (MCF-7) is associated with increased EMT (Desai et al., 2017, Tatler et al., 2016). Also consistent with ELK1 playing a role in the association between X chromosome dosage and differentiation outcome is a previous study showing that *ELK1* overexpression or downregulation, respectively, mimics the phenotypes of XaXa or XaXi PSCs (Bruck et al., 2013). Of note, atrioventricular septal defects occur in ∼20% of individuals with Down Syndrome (DS), and have a higher prevalence in female DS patients (Diogenes et al., 2017). Given that EPDCs play an essential role in septal formation (Gittenberger-de Groot et al., 2000), our study suggests that future work should investigate the extent to which X chromosome gene-expression levels are altered in cardiomyocytes from individuals with DS, and whether this is associated with the formation of fewer EPDCs.

Overall, our study suggests that expression differences of 91 signature and X chromosome genes result in the iPSCORE iPSC lines having differential propensities to respond to WNT inhibition during differentiation, and consequently are fated to produce iPSC-CVPC samples with different proportions of CMs and EPDCs. As iPSCs in the iPSCORE collection have passed standard quality checks to confirm their pluripotency and genomic integrity (Banovich et al., 2018, Panopoulos et al., 2017a), these transcriptomic expression differences associated with cardiac lineage outcome are not detected using current quality metrics. In conclusion, our findings suggest that to derive human iPSC lines that respond similarly in differentiation protocols, it may be necessary to improve reprogramming methods such that the transcriptome and X chromosome activation state is fully reset to the naive state, and incorporate inactivation of one of the X chromosomes in female lines as an early step in differentiation protocols.

## Experimental Procedures

Please refer to Supplemental Experimental Procedures for detailed methods.

### Subject Information and Whole-Genome Sequencing

Individuals (108 females and 73 males) were recruited as part of the iPSCORE project (Panopoulos et al., 2017a) and included 7 MZ twin pairs, members of 32 families (2–10 members/family), and 71 singletons and were of diverse ancestries. Subject descriptions including subject sex, age, family, ethnicity, and cardiac diseases were collected during recruitment. As previously described (DeBoever et al., 2017), we generated whole-genome sequences from the blood or skin fibroblasts of the 181 subjects on the HiSeqX (Illumina; 150-bp paired end). The recruitment of these individuals was approved by the Institutional Review Boards of the University of California, San Diego and The Salk Institute (project no. 110776ZF).

### iPSC Derivation and Somatic Mutation Analysis

As previously described, we reprogrammed fibroblast samples using non-integrative Cytotune Sendai virus (Life Technologies), and the 191 iPSCs (seven subjects had two or more clones each) were shown to be pluripotent and to have high genomic integrity with no or low numbers of somatic copy-number variants (CNVs) (D'Antonio et al., 2018, Panopoulos et al., 2017a).

### Large-Scale Derivation of iPSC-CVPC Samples

To generate iPSC-derived cardiovascular progenitors (iPSC-CVPCs), we used a small-molecule cardiac differentiation protocol (Lian et al., 2013). The 25-day differentiation protocol consisted of five phases (Figure S1A); the optimizations for each step are described in the Supplemental Experimental Procedures.

### Flow Cytometry

On day 25 of differentiation, iPSC-CVPCs were stained with cTnT antibody, acquired using fluorescence-activated cell sorting and analyzed using FlowJo V10.2.

### Immunofluorescence Analysis of iPSC-CVPCs

Immunofluorescence was assessed in five iPSC-CVPC lines. Live frozen iPSC-CVPC harvested on day 25 were thawed, plated for 5 days, fixed, permeabilized, and incubated with antibodies (Table S1D).

### Generation of RNA-Seq Data

For gene-expression profiling of iPSCs, we used RNA-seq data from 184 samples (cell lysates collected between passages 12 and 40). For gene-expression profiling of iPSC-CVPCs, we generated RNA-seq data from 180 samples at day 25 of differentiation. All RNA-seq samples were generated and analyzed using the same pipeline to obtain transcripts per million base pairs (TPM) (DeBoever et al., 2017).

### Generation of scRNA-Seq Data

To capture the full spectrum of heterogeneity among the iPSC-CVPCs, we selected eight samples with variable percentage of cTnT (42.2%–95.8%). After removing proliferating cells and doublets, we obtained 34,905 cells.

### CIBERSORT

Expression levels of the top 50 genes overexpressed in each of the three cell populations (total 150 genes) were used as input for CIBERSORT (Newman et al., 2015) to calculate the relative distribution of the three cell populations for the 180 iPSC-CVPC samples at day 25.

### Characterizing Transcriptional Similarities of iPSCs, iPSC-CVPCs, and GTEx Adult Tissues

We performed PCA of RNA-seq on 184 iPSCs, 180 iPSC-CVPCs, and 1,072 RNA-seq samples from GTEx.

### Determining Optimal CM/EPDC Ratio Estimates from CIBERSORT to Define iPSC Cardiac Fates

To obtain the optimal threshold, we conducted a series of differential expression analyses on 15,228 autosomal genes in the 184 iPSC lines (147 completed and 37 terminated) considering the ratio of population frequencies at ten thresholds. The 30:70 (CM/EPDC) ratio resulted in the highest number of differentially expressed genes (84 genes with Storey q value <0.1, t test), which is substantially greater than random expectation. Thus, we grouped the 184 iPSC lines into: (1) those that have CM fates, i.e., produced iPSC-CVPC with ≥30% population 1; and (2) those that have EPDC fates, i.e., produced iPSC-CVPC with >70% population 2.

### Comparing the Number of Differentially Expressed Genes with Random Expectation

To determine whether the number of significantly differentially expressed genes was higher than expected by chance, we shuffled the assignments of the 184 iPSC RNA-seq samples to differentiation fate (125 CM and 59 EPDC) 100 times.

### Contribution of 91 Signature Genes in iPSCs to Determination of Cardiac Fate

For each of the 91 signature genes, we built a GLM with the expression of the gene as input and the differentiation outcome (e.g., percentage of population 1) as output using a logit link function. To understand the cumulative contribution of all 91 signature genes on cardiac differentiation fate, we built a GLM with an L1 norm penalty using the expression of all 91 genes as input and the differentiation outcome as output. To avoid overfitting the model, we used a 10-fold cross-validation.

### Detecting Associations between Genetic Background and Differentiation Outcome

We obtained genotypes for 8,620,159 biallelic SNPs and short indels with allelic frequency >5% in the iPSCORE collection. Genotypes were obtained for each SNP in all individuals using *bcftools view* (Li, 2011). Linear regression was used to calculate the associations between the genotype of each variant and differentiation outcome (percent CM population in the iPSC-CVPCs), using passage at monolayer and sex as covariates.

### GSEA Using the MSigDB Collection

We performed GSEA using the R *gage* package (Luo et al., 2009) on all MSigDB gene sets (Subramanian et al., 2005). False discovery rate correction was performed independently for each collection. The normalized mean expression difference between iPSCs that differentiated to CMs and iPSCs that differentiated to EPDCs was used as input for GSEA.

### Associations between iPSC and Subject Features and Differentiation Outcome

A GLM was built in R using age, sex, ethnicity, age, and passage of the iPSCs at day 0 of differentiation as input and differentiation outcome as output (0 = EPDCs; 1 = CMs).

### Identifying X Chromosome Inactivation in Female iPSCs and iPSC-CVPCs

To analyze X chromosome inactivation, we used 113 female iPSCs, of which 87 where CM-fated and 26 were EPDC-fated. We called ASE in RNA-seq from iPSC and iPSC-CVPCs as previously described (DeBoever et al., 2017). Genes lying in X chromosome pseudoautosomal (PAR) regions (PAR1: 60,001–2,699,520; PAR2: 154,931,044–155,260,560) were removed from analysis. We defined the strength of ASE for each gene as the fraction of RNA transcripts that were estimated to originate from the allele with higher expression (referred to as AIF).

### Validation of Findings in Yoruba iPSC Set

The Yoruba iPSCs (Banovich et al., 2018) were generated from LCLs using episomal reprogramming. Differentiation was performed using a small-molecular method and iPSC-CMs were harvested on days 31 or 32. Fifteen lines successfully generated iPSC-CMs and 24 were terminated on or before day 10. We downloaded RNA-seq for 34 of the Yoruba iPSC and 13 iPSC-CM samples from the Gene Expression Omnibus (GEO: GSE89895) as well as 297 samples from 19 distinct iPSCs in a time-course experiment (days 0–15) performed on the same Yoruba iPSC samples (Strober et al., 2019). RNA-seq was aligned using STAR, and gene expression was quantified using the RSEM package and normalized to TPM. The RNA-seq for the 13 Yoruba iPSC-CMs and from all time-course time points were analyzed using CIBERSORT, similar to the iPSCORE samples.

## Author Contributions

K.A.F., A.D.-C., M.K.R.D., and M.D. conceived the study. A.D.-C. and K.F. performed iPSC-CVPC differentiations. A.D.-C. and F.C. generated molecular data. S.H. generated immunofluorescence images. M.C.W. generated Yoruba iPSC-CMs. F.S. and A.D.-C. generated scRNA-seq data. M.K.R.D., W.W.G., H.M., E.N.S., J.P.N., and M.D. performed data processing and computational analyses. M.P., E.A., and K.A.F. oversaw the study. M.K.R.D., M.D., and K.A.F. prepared the manuscript.

## Accession Numbers

Accession numbers for the RNA-seq data, scRNA-seq, and WGS genotypes are dbGaP: phs000924 (https://www.ncbi.nlm.nih.gov/projects/gap/cgi-bin/study.cgi?study_id=phs000924) and phs001325 (https://www.ncbi.nlm.nih.gov/projects/gap/cgi-bin/study.cgi?study_id=phs001325). The 191 iPSC lines are available through WiCell Research Institute: https://www.wicell.org/; NHLBI Next Gen Collection.

## Supplemental Information

Document S1. Supplemental Experimental Procedures, Figures S1–S7 and Detailed Supplemental Table Legends

Table S1. Characterization of Cellular Heterogeneity in iPSC-CVPC Samples, Related to Figure 1 (A) Subject information for participants in iPSCORE for which iPSCs were used for iPSC-CVPC differentiation. (B) Table linking identifiers for iPSCORE participants with iPSC-CVPC differentiations and metrics of differentiation outcome. (C) Table describing the number of lines and subjects for each attempted differentiation. (D) Antibodies used for fluorescence-activated cell sorting and immunofluorescence. (E) Table linking identifiers for iPSC and iPSC-CVPC genomic data. (F) Generated molecular data. (G) scRNA-seq features for each sequenced single cell. (H) Table describing observed beat scores and structure scores for iPSC-CVPC differentiations.

Table S2. Overexpressed Genes in Each scRNA-Seq Population, Related to Figure 2

Table S3. iPSC Gene Signatures Associated with Cardiac Differentiation Fate, Related to Figure 3 (A and B) Differential expression between iPSCs differentiated to CMs and iPSCs differentiated to EPDCs using multiple thresholds. (C) Differential expression analysis using 30:70 CM/EPDC ratio as threshold. (D) Description and supporting literature of 91 signature genes in iPSCs. (E) Regression estimates showing the associations between signature genes and %CM populations. (F) Associations between genetic variation and differentiation outcome.

Table S4. X Chromosome Gene Dosage Plays a Role in Cardiac Differentiation Fate, Related to Figures 4 and 5 (A) GSEA showing functional enrichment of genes differentially expressed between CM-fated and EPDC-fated iPSCs. (B) Table describing results of linear regression analysis to predict factors influencing differentiation potential of iPSCs toward CM or EPDC fates, related to Figure 4. (C) Allelic imbalance fraction of genes on the X chromosome not in pseudoautosomal regions in females from iPSC samples and from iPSC-CVPC samples, related to Figure 4.

Table S5. Table Describing Differentiation Outcomes and Molecular Data ID References from the Yoruba Set, Related to Figure 5

Document S2. Article plus Supplemental Information
