# Supplementary material for: Association of Human iPSC Gene Signatures and X Chromosome Dosage with Two Distinct Cardiac Differentiation Trajectories
Source: Stem Cell Reports. 2019 Oct 24;13(5):924–38. doi: 10.1016/j.stemcr.2019.09.011 (PMC6895695; doi:10.1016/j.stemcr.2019.09.011)
Supplement: Document S1. Supplemental Experimental Procedures, Figures S1–S7 and Detailed Supplemental Table Legends [file mmc1.pdf]

Stem Cell Reports, Volume *13*

Supplemental Information

Association of Human iPSC Gene Signatures and X Chromosome Dosage with Two Distinct Cardiac Differentiation Trajectories

Agnieszka D'Antonio-Chronowska, Margaret K.R. Donovan, William W. Young Greenwald, Jennifer Phuong Nguyen, Kyohei Fujita, Sherin Hashem, Hiroko Matsui, Francesca Soncin, Mana Parast, Michelle C. Ward, Florence Coulet, Erin N. Smith, Eric Adler, Matteo D'Antonio, and Kelly A. Frazer

SUPPLEMENTAL MATERIAL

TABLE OF CONTENTS

SUPPLEMENTAL FIGURES 2-14

Figure S1. Optimization of iPSC-CVPC differentiation protocol 2-4

Figure S2: Distribution of single cells across the three clusters 5-6

Figure S3. Differentially expressed genes at ten CM:EPDC thresholds 7-8

Figure S4. Expression of 91 signature genes in 184 iPSCs 9-10

Figure S5. Associations between genetic background and differentiation outcome 11

Figure S6. X chromosome inactivation in iPSCs 12-13

Figure S7. Cell populations at 15 time points during iPSC-CM differentiations 14

TABLE LEGENDS 15-20

SUPPLEMENTAL EXPERIMENTAL PROCEDURES 20-29

REFERENCES 30-31

Figure S1: Optimization of iPSC-CVPC differentiation protocol. Related to Figure 1.

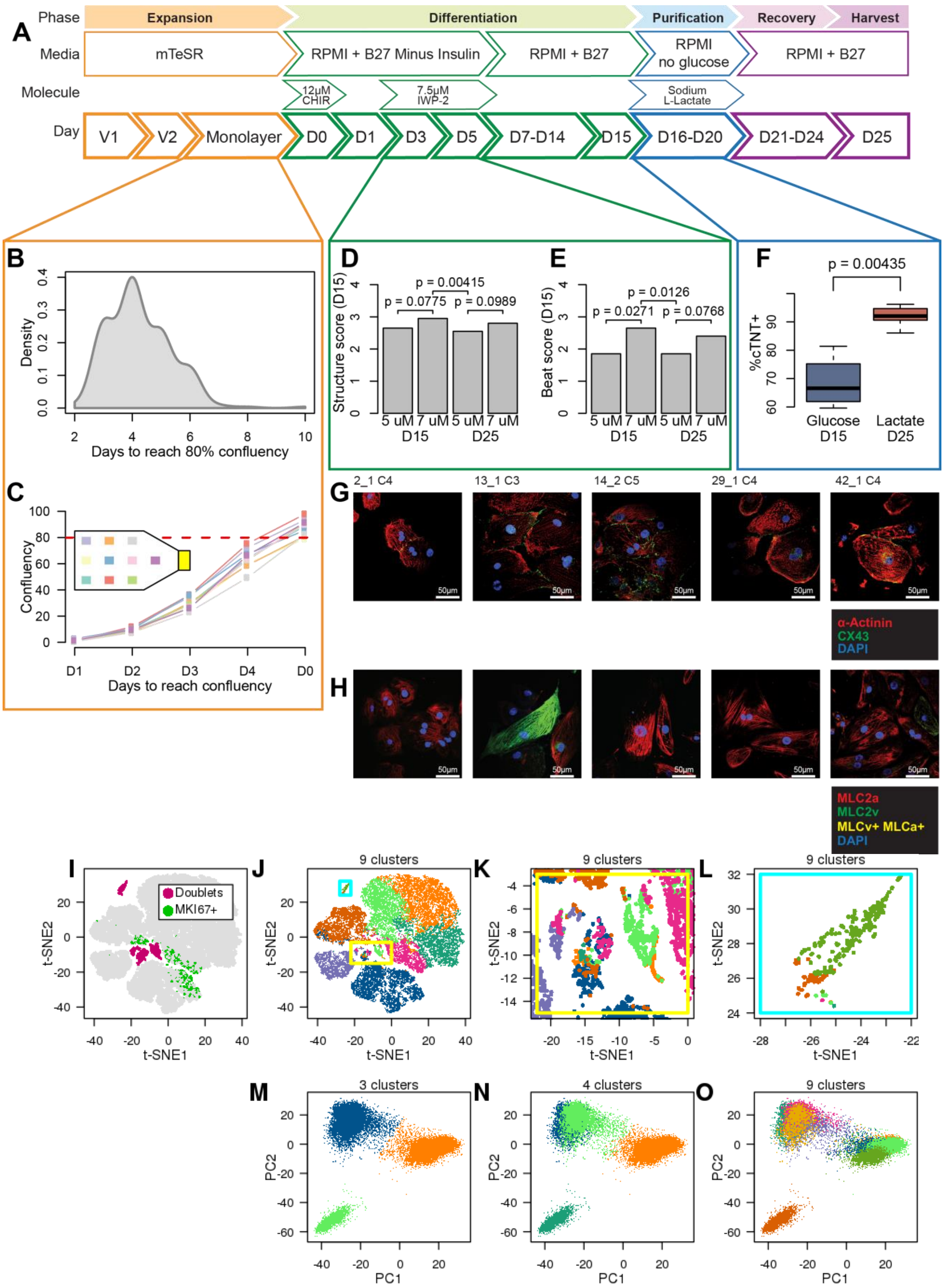

(A) Schematic of differentiation protocol. To achieve large-scale derivation of iPSC-derived cardiovascular progenitor cells (iPSC-CVPCs), we optimized existing small molecule protocols to increase throughput and efficiency. We optimized several steps of the protocol, including automating detection of iPSC monolayer confluency (orange box), optimizing the IWP-2 concentration (green box), and incorporating lactate selection (blue box).

(B) Density plot showing distribution of days recorded from 253 iPSC samples to reach 80% confluency. It was observed that 75-85% iPSC monolayer confluence at day 0 (D0), which marks the initiation of differentiation by WNT activation, yields the most efficient differentiations (Burridge et al., 2014; Lian et al., 2013); however, iPSCs have variable growth rates, and therefore it would be difficult to consistently achieve this confluency across hundreds of lines.

(C) Confluency levels from one line (2_3) measured from ten sections of T150 flask. Due to observed variability in iPSC growth rates, we developed ccEstimate, an automated tool that determines confluency by processing images from multiple locations in three T150 flasks over a period of at least 72 hours, and then estimates when a particular iPSC line will reach an average confluency of 80% based on its growth rate (Figure S2). Circles represent measured values. The points at D0 were obtained based on the ccEstimate algorithm's predictions.

(D, E) Effects of IWP-2 concentration (5.0µM or 7.5µM) given on D3 or D3 and D4 on (D) structure score and (E) beat score (Table S1H). We optimized WNT inhibition at D3, which is required for robust iPSC-CVPC differentiations by testing two concentrations of IWP-2 (5µM and 7.5µM) both with and without a media change between D3 and D4. We differentiated one iPSC line (iPSCORE_2_3_iPSC_C5_P13) under each of the four IWP-2 conditions, and observed that 7.5 µM IWP-2 without a media change between D3 and D4 resulted in iPSC-CVPCs with the thickest structures and strongest beating (structure score: $p = 0.00415$, beat score: $p = 0.0126$; Paired t test) (Table S1H). P-values were calculated using paired t test.

(F) Effects of metabolic purification of iPSC-CVPCs by lactate and glucose. To examine the efficacy of using lactate for iPSC-CVPC metabolic purification (Burridge et al., 2014; Kadari et al., 2015; Tohyama et al., 2013), we tested lactate and glucose at D16 in three different iPSC-CVPC lines (2_3, 8_2, and 3_2), and found that lactate resulted in significantly purer iPSC-CVPC populations at D25 (93.95% vs. 68.55%; $p = 0.00435$). P-values were calculated using Mann-Whitney U test.

(G) Immunofluorescence staining of five iPSC-CVPC lines at D30 with IF markers DAPI (blue), ACTN1 (red), and CX43 (green).

(H) Immunofluorescence staining of five iPSC-CVPC lines at D30 with IF markers DAPI (blue), MLC2a+ (red), and MLC2v+ (green), and MLC2v+ MLC2a+ (yellow).

(I) Filtering doublets from and selection of k-means clustering k in scRNA-seq: t-SNE plot of gene expression from 36,839 cells from 8 iPSC-CVPC and 1 ESC. We removed 1,934 cells from the scRNA-seq analysis (Figure 1), including cells that were visually identified as being doublets (pink) and actively dividing cells with (>2 UMI MK167, green).

(J) Cells are colored by k-means clusters with k = 9 cluster assignment. Cells that clustered together in the t-SNE plot, but were assigned to multiple different clusters were considered as doublets. Doublets are highlighted in the yellow and cyan box.

(K) Zoomed in region of the yellow box.

(L) Zoomed in region of the cyan box.

(M-O) PCA of gene expression from 36,839 cells from 8 iPSC-CVPC and 1 ESC colored by k-means clustering: (M) k = 3, (N) k=4; and (O) k = 9. The PCA shows that three cell populations are present and thus we used three clusters (k = 3) for all scRNA-seq analysis. In summary, we analyzed 34,905 single cells assigned to three cell populations.

Figure S2: Distribution of single cells across the three clusters. Related to Figure 1.

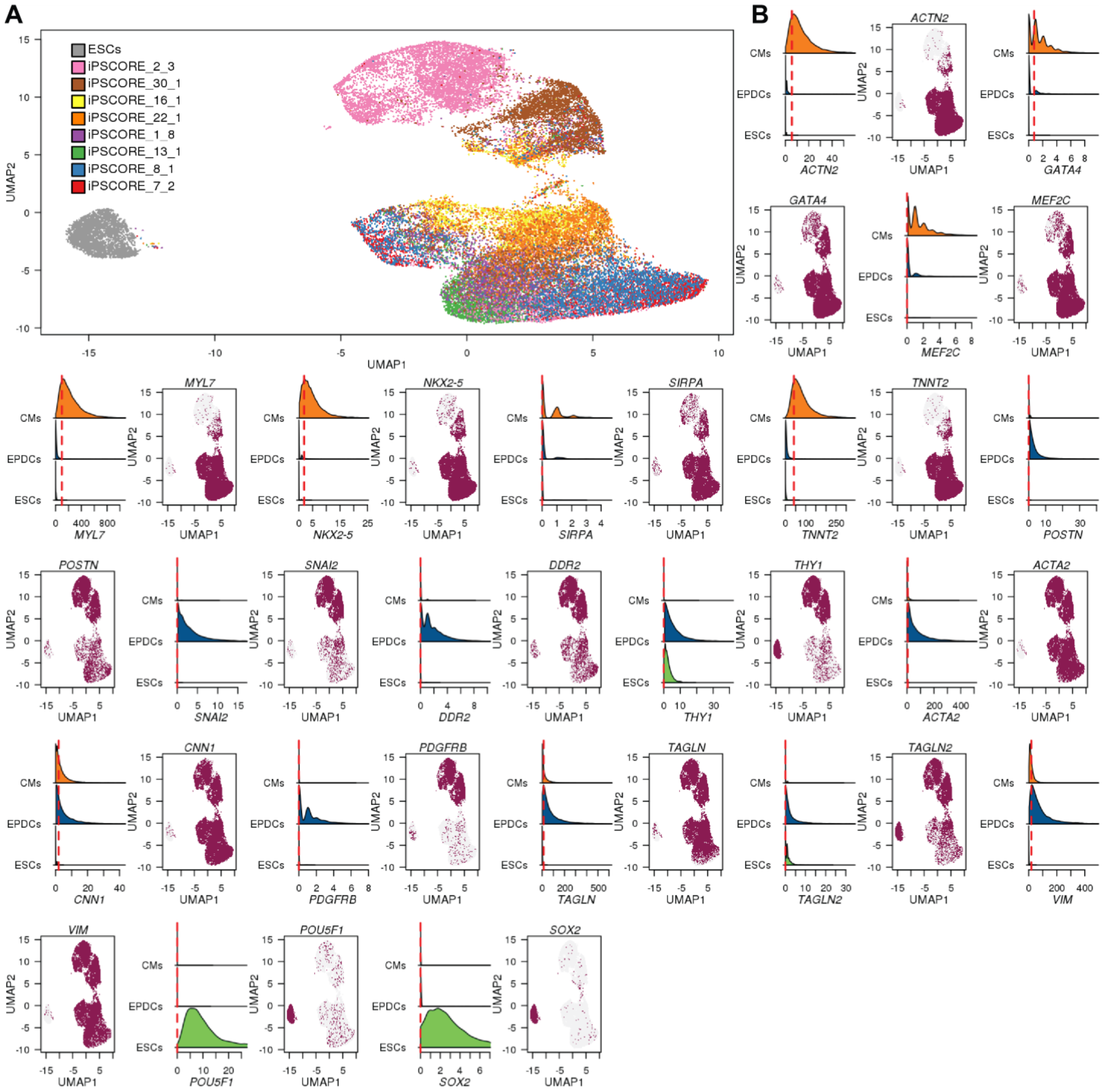

(A) Distribution of single cells across the three cell populations for the nine analyzed samples: scRNA-seq UMAP plots from 34,905 single cells showing their distributions across the three different clusters for the nine analyzed samples (8 iPSC-CVPCs lines and one ESC line). Each of the nine samples have a different color.

(B) Expression levels for marker genes: For each gene in Figure 1J, density plots show the gene expression distribution across all cells associated with each cell population (Population 1 = orange; Population 2 = blue;

Population 3 = green.). Red dashed line represents the median. UMAP plots from 34,905 cells show in maroon all the cells expressing the indicated marker gene higher than its median expression across the three populations.

Figure S3: Differentially expressed genes at ten CM:EPDC thresholds. Related to Figure 3.

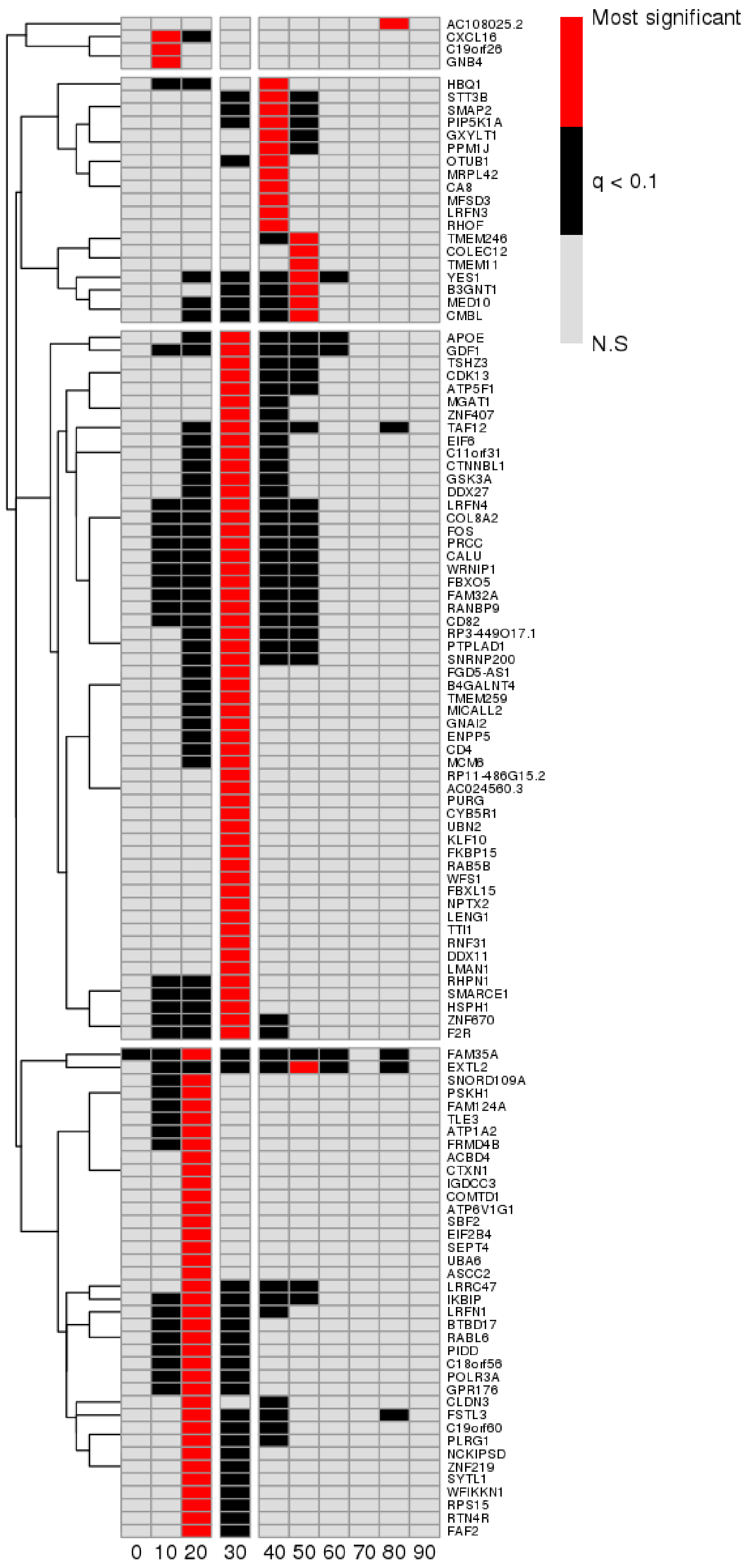

Analysis to determine the optimal CM:EPDC ratio to group the iPSC lines into those that were CM-fated and those that were EPDC-fated. We tested ten different CM:EPDC ratios and found 116 autosomal genes that were differentially expressed at one or more of these ratios (Storey q-value < 0.1, t-test Figure 3A,B, Table S3A-B). Heatmap showing at each threshold whether each of the 116 differentially expressed genes is significant (red or black = q-value < 0.1; gray: q-value > 0.1). Red squares indicate the threshold at which each gene is most significant. The heatmap shows that the 30:70 (CM:EPDC) threshold is where the most genes have their highest significance. For the vast majority of genes, the threshold at which they are most significantly differentially expressed is between 20% and 40%, confirming that the 30% threshold is optimal to distinguish between CM-fated and EPDC-fated iPSCs.

Figure S4: Expression of 91 signature genes in 184 iPSCs. Related to Figure 3.

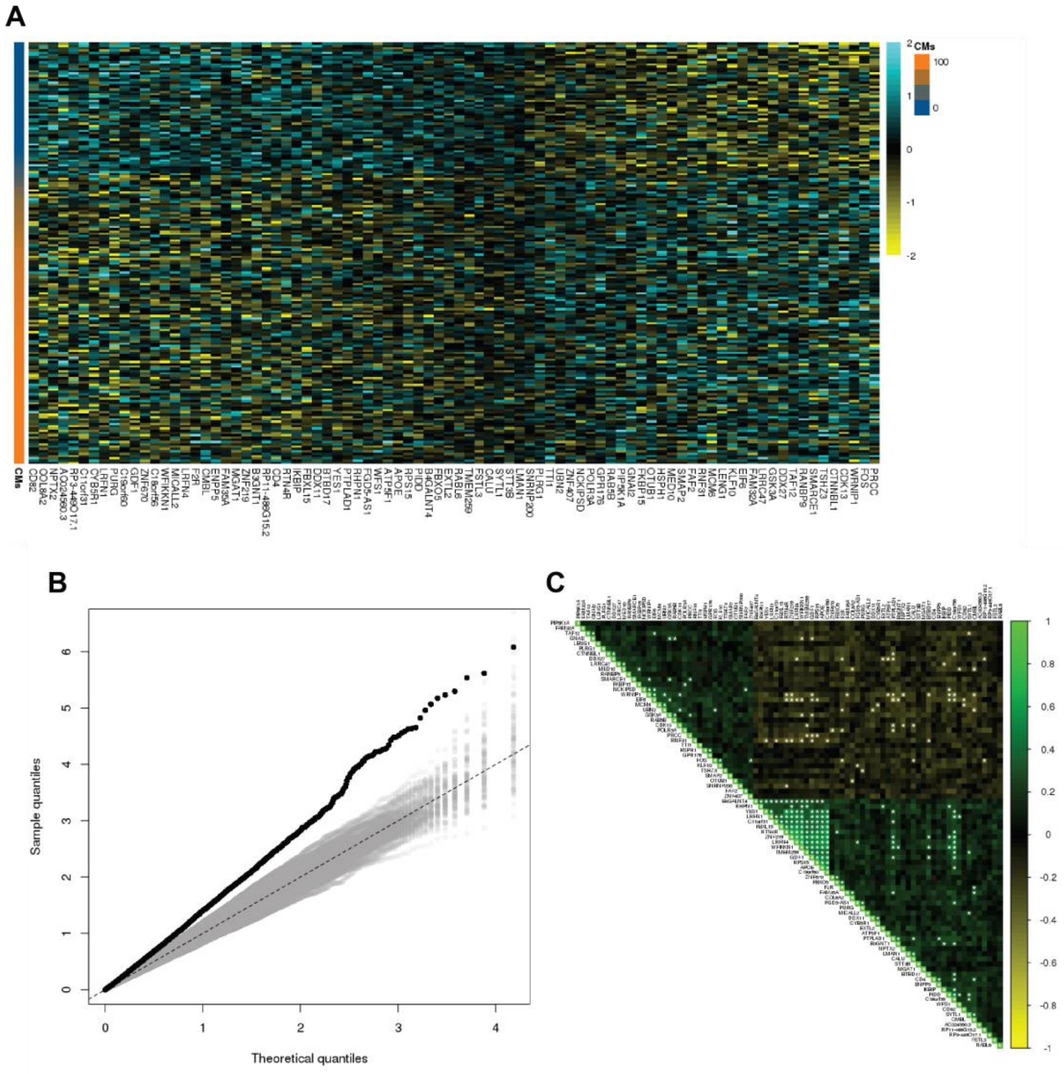

(A) Heatmap showing the expression levels of the 91 signature genes differentially expressed between CM-fated and EPDC-fated iPSCs. Each row represents an iPSC sample. The "CMs" scale represents the %CM population (Population 1) in the associated iPSC-CVPC samples for each iPSC.

(B) Comparison between the observed number of signature genes and random expectation: A QQ plot showing that the observed p-value distribution (black) was substantially different than random expectation. To determine if the identification of 84 signature genes that were significantly differentially expressed between CM-fated and EPDC-fated iPSCs was higher than random expectation, we shuffled the assignments of the 184 iPSC RNA-seq samples to differentiation fates (125 CM and 59 EPDC) 100 times. For each shuffle, we performed differential expression analysis and obtained the number of genes that were significantly differentially expressed (gray).

(C) Correlation between the 91 signature genes differentially expressed between CM-fated and EPDC-fated iPSCs: Heatmap showing the correlation of expression levels in the 125 CM-fated iPSCs versus the 59 EPDC-fated iPSCs for the 91 signature genes. White stars show significant correlations (Bonferroni p-value < 0.05).

Figure S5: Associations between genetic background and differentiation outcome

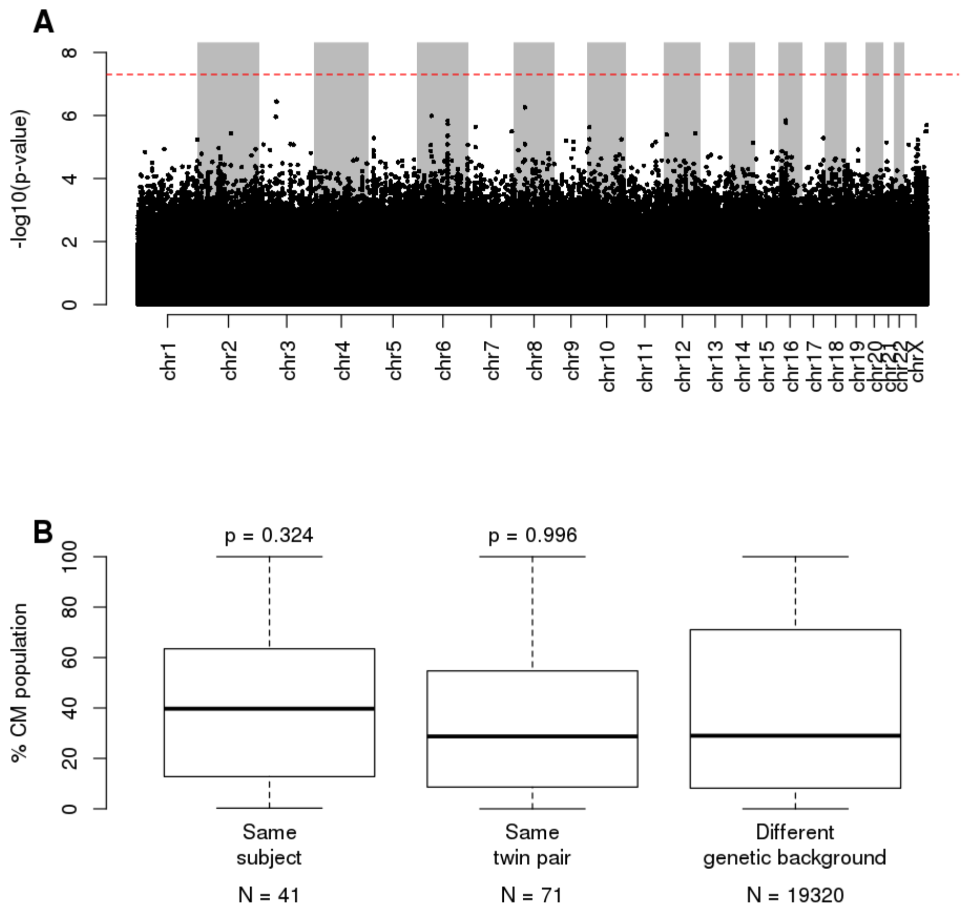

(A) Manhattan plot showing the association between genetic variation and differentiation outcome (measured as % CM population in iPSC-CVPCs). Red dashed line shows p-value = 0.05 adjusted using Bonferroni's method ($p = 5 \times 10^{-8}$).

(B) Boxplots showing distributions of the differences in the %CM population between differentiations of different iPSC clones from the same subject, from the same twin pair, and from individuals with different genetic backgrounds. P-values were calculated using Mann-Whitney U test.

Figure S6: X chromosome inactivation in iPSCs. Related to Figure 4.

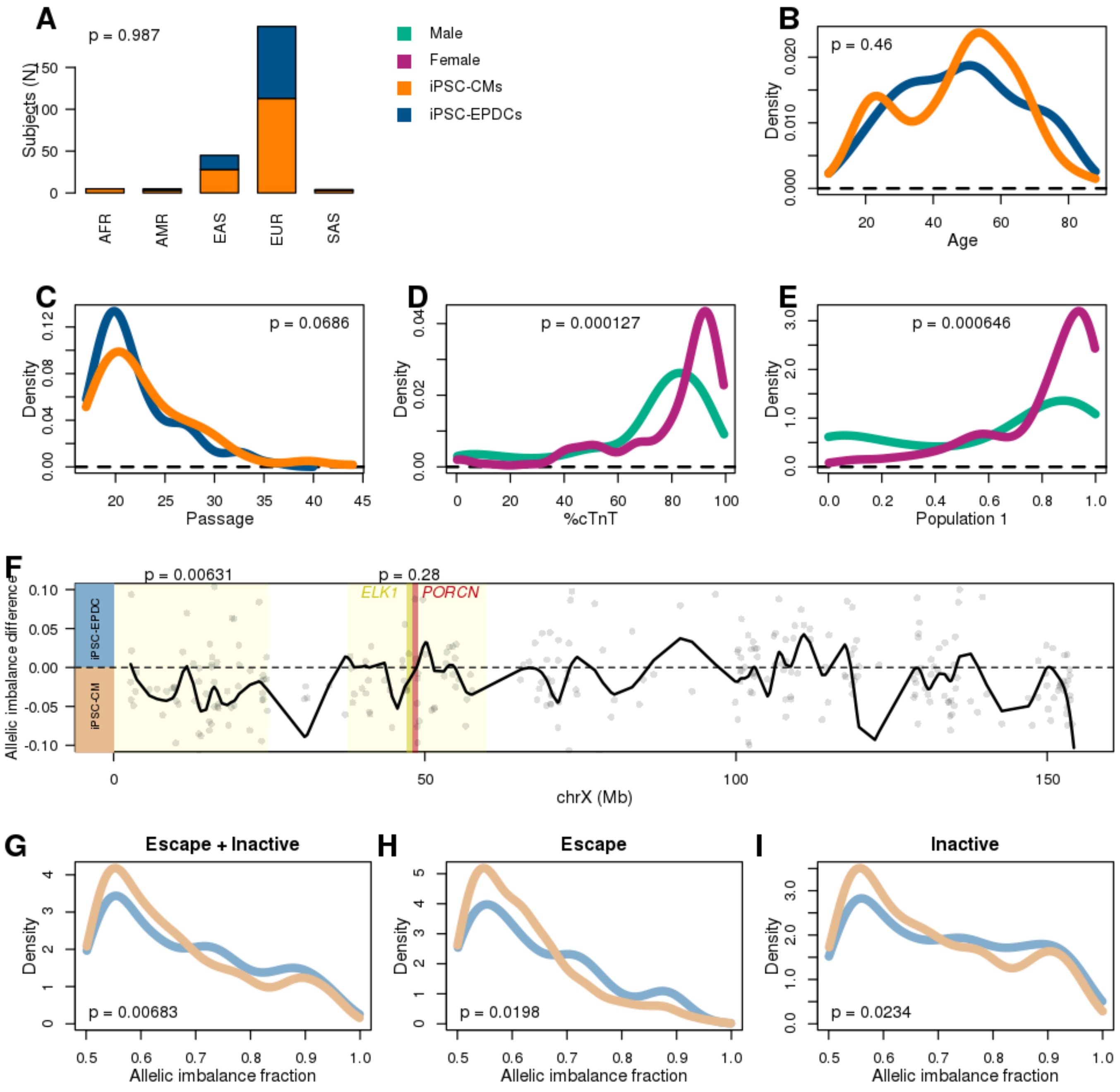

(A-C) Associations between differentiation outcome (orange: iPSC-CVPC samples with CM fraction > 30%; blue: with EPDC fraction > 70%) and (A) ethnicity (most similar superpopulation from the 1000 Genomes Project), (B) age at enrollment, and (C) passage at monolayer (D0). (A) is shown as barplots; (B,C) are shown as density plots. P-values were calculated using Z-test (glm function in R).

(D-E) Density plots showing the association between sex (teal: males; magenta: females) and (D) %cTnT, and (E) fraction of CM population for 191 iPSC lines. P-values were calculated using Mann-Whitney U test.

(F) Allelic imbalance difference between CM-fated and EPDC-fated iPSCs. The dots represent each gene on chrX, while the black solid line corresponds to the smoothed interpolation of differences for all the genes. The locations of the Xp22 and Xp11 loci on the chrX G-banding ideogram are highlighted in yellow, as well as *ELK1* (yellow) and *PORCN* (red). P-values above each locus indicate the difference in allelic imbalance between CM-fated and EPDC-fated iPSCs in each locus (Mann Whitney U test).

(G-I) Allelic imbalance fraction from inactive and escape genes in Xp22: Density plots showing the allelic imbalance differences in chrX genes on the Xp22 loci in female samples between iPSC lines with CM-fate (light blue) and EPDC-fate (light orange) differentiations. Allelic balances compared in Xp22 are from all genes in the region (G), escape genes (H), and inactive genes (I). P-values were calculated using Mann Whitney U test.

Figure S7: Cell populations at 15 time points during iPSC-CM differentiations

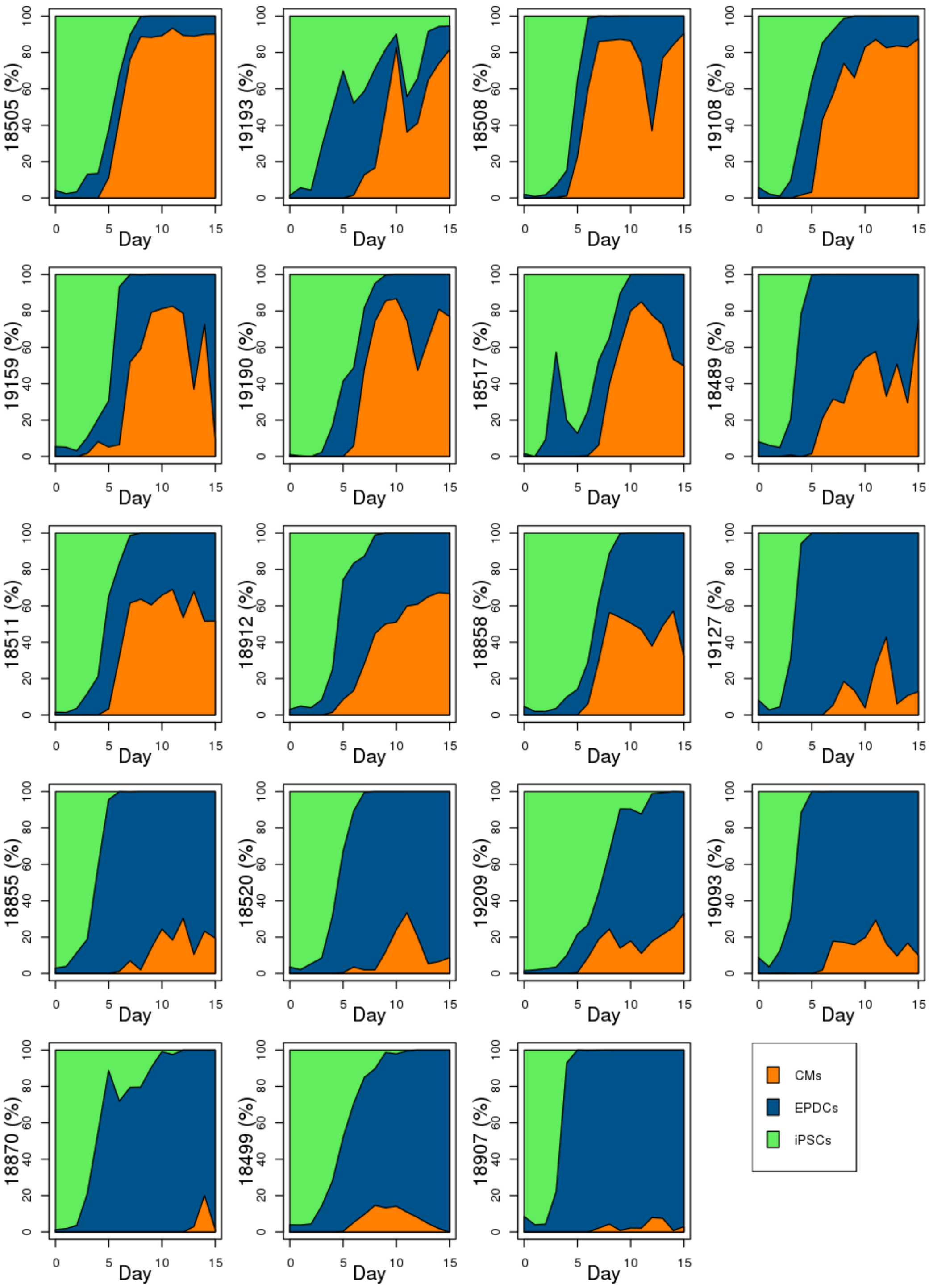

For each of 19 samples in Strober et al. (Strober et al., 2019), at each day during differentiation the relative distributions of iPSCs, CMs, EPDCs are shown. **Related to Figure 5.**

TABLE LEGENDS

Table S1: Characterization of cellular heterogeneity in iPSC-CVPC samples. Related to Figure 1.

Table S1A: Subject information for participants in iPSCORE for which iPSCs were used for iPSC-CVPC differentiation.

iPSCORE_ID indicates family and individual number (e.g. iPSCORE_family#_individual#). Subject_UUID is an assigned Universal Unique Identifier (UUID) for the subject. Family_ID classifies the subject by family to identify related family members. Columns D-G represent the twin and parent information for each subject, as included in dbGaP (phs001325.v1.p1; phs000924.v1.p1) as part of the iPSCORE Resource: Twin_ID_dbgap identifies the dbGaP id if the subject is a twin; Twin_type_dbGap indicates the type of twin (MZ = monozygotic; DZ = dizygotic) if the subject is a twin; Father_subject_ID_dbGap indicates the subject_UUID of the father of the subject if part of the iPSCORE resource; Mother_subject_ID_dbGap indicates the subject_UUID of the mother of the subject if part of the iPSCORE resource. Sex and Age_at_enrollment of the subject are shown. Ethnicities (Self-reported race/ethnicity, Recorded_Ethnicity_Grouping, and Most_similar_1KGP_population) are recorded as described by Panopoulos et al. (Panopoulos et al., 2017). Column M represents cardiac phenotypes.

Table S1B: Table linking identifiers for iPSCORE participants with iPSC-CVPC differentiations and metrics of differentiation outcome.

Unique Differentiation IDentifier (UDID) is a unique digit assigned for each attempted iPSC-CVPC differentiation. iPSCORE_ID indicates family and individual number (e.g. iPSCORE_family#_individual#). Subject_UUID is an assigned Universal Unique IDentifier (UUID) for the subject. iPSC_iPSCORE_ID is the iPSC line identifier submitted to dbGap (phs001325.v1.p1), which indicates clone and passage of iPSC. iPSCORE_resource indicates by TRUE or FALSE if this line is one of the 222 lines described by Panopoulos et al.(Panopoulos et al., 2017). iPSC_ID is the iPSC line identifier. iPSC_passage_at_monolayer (D0) is reported. D_to_D0 describe how many days the iPSC line was cultured to achieve 80% confluency before initiation of differentiation. If the UDID was harvested on D25 (Column I), the harvest density (Column J), number of cryovials frozen (Column K), and measured %cTNT+ by FACS (Column L) is reported. Successful_iPSC_CM_differentiation indicates if the iPSC-CVPC sample was harvested at D25 (e.g. not prematurely terminated). Population_1 indicates the estimated composition of population 1 (cardiomyocyte population) for each sample with RNA-seq (column M, see Table S1E) and the estimated_cell_type (Column N)

indicates iPSC-CVPC samples with ≥30% population 1 as CM and iPSC-CVPC samples with <30% population 1 as EPDC.

Table S1C: Table describing the number of lines and subjects for each attempted differentiation.

For each cell type: iPSC and derived iPSC-CVPCs (both terminated prior to D25 and D25), the number of differentiations performed (Column B) are given. For these differentiations, the number of unique lines used (Column C) from the number of unique subjects used (Column D) is provided.

Table S1D: Antibodies used for FACS and immunofluorescence.

This table describes the antibodies (Column A) and clone (Column B) used for FACS and immunofluorescence experiments. Catalog numbers (Column C), brand (Column D), dilution (Column E), time of staining in minutes (Column F), and temperature of staining (Column G) is indicated for each antibody.

Table S1E. Table linking identifiers for iPSC and iPSC-CVPC genomic data.

UDID is given if the iPSC or iPSC-CVPC genomic data were collected during an attempted differentiation, indicated by UDID (Column A). Subject_UUID (Column B) is an assigned Universal Unique Identifier (UUID) for the subject. Cell (Column C) indicates the stage for which the genomic data was generated (iPSC or iPSC-CVPC). Genomic data UUIDs are given in Columns D-E, including rna_assay_uuid (bulk RNA-seq; Column D), scrna_assay_uuid (scRNA-seq; Column E). Estimated cellular composition from CIBERSORT of populations 1-3 is given in Columns F-H.

Table S1F. Generated molecular data.

For each cell type (iPSC and iPSC-CVPC) (Column A) and for each assay for which molecular data was generated (RNA-seq and scRNA-seq), the number of data samples (Column C), from the number of unique lines (Column D), and from the number of unique subjects (Column E) are given.

Table S1G: scRNA-seq features for each sequenced single cell.

For each of the 34,905 cells with scRNA-seq data, the table shows iPSCORE subject ID (Column A) and UUID (Column B), barcode (Column C), associated population (Column D), and coordinates on the t-SNE plot (Columns E, F). For the H9 ESC line sample, which is not included in iPSCORE, iPSCORE ID and Subject UUID are labeled as “ESCs”. This table is ordered on population (e.g. clusters 1, 2, 3).

Table S1H: Table describing observed beat scores and structure scores for iPSC-CVPC differentiations.

UDID (Column A) for each differentiation measured is given. Beat.Score (Column B) indicates the estimated beat score for the differentiation and Structure.Score indicates the observed structure score for the sample (Column C).

Table S2: Overexpressed genes in each scRNA-seq population. Related to Figure 2.

For each of 34,528 genes (Columns A, B) with at least one transcript detected in the scRNA-seq samples, the mean UMI counts, log2 fold change, and FDR-adjusted p-value is shown for each population. The last column indicates the 150 genes used as input for CIBERSORT.

Table S3: iPSC gene signatures associated with cardiac differentiation fate. Related to Figure 3.

Table S3A-B: Differential expression between iPSCs differentiated to CMs and iPSCs differentiated to EPDCs using multiple thresholds

We used 10 different thresholds to divide iPSCs based on their %CM population detected using CIBERSORT (Figure S7). For each threshold (Columns B-M) (>0%, 10%, 20%, 30%, 40%, 50%, 60%, 70%, 80%, 90%; males and females; EPDC fate vs. Terminated), differential expression between the samples passing and not passing the threshold was calculated using t-test. Table S3A and Table S3B respectively show nominal p-values and Storey q-values. P-values of differentially expressed genes calculated by t-test are shown for all 15,228 human genes (Column A) expressed in iPSC-CVPCs. We examined genes that were differentially expressed in the iPSCs generated from females versus males (column L), and removed 242 genes that were differentially expressed from downstream analyses (Storey q-values < 0.1). Across the ten CM:EPDC ratios there were 116 genes that were differentially expressed at one or more ratio. At the 30% threshold, there were the greatest number of significantly differentially expressed genes. EPDC-fated iPSCs consisted of those with completed iPSC-CVPC differentiations (i.e. reached D25) with >70% Population 2 and iPSCs with differentiations that were terminated before D25. We did not observe significant expression differences between the 22 iPSCs that differentiated to EPDCs (>70% Population 2) and the 37 iPSCs whose differentiations were terminated before D25 (column M). For further analyses, we used the 30:70 (CM:EPDC) threshold, because it maximized the differences between CMs and EPDCs, i.e. the largest number of differentially expressed genes (84).

Table S3C: Differential expression analysis using 30:70 CM:EPDC ratio as threshold.

In this table, we report the differential expression analysis performed between the 125 iPSC samples defined as CM-fated and 59 iPSC samples defined as EPDC-fated (15,228 autosomal genes, labeled as "All iPSC samples" in column H). We also report the differential expression analysis performed between the 87 female iPSC samples defined as CM-fated and the 26 female iPSC samples defined as EPDC-fated (15,398 expressed genes located on both autosomes and on the chromosome X, labeled as "Female samples" in column H). For each gene (Columns A, B), shown are: 1) the mean normalized expression across the CM-fated iPSCs (column C), 2) the mean normalized expression across the EPDC-fated iPSCs (column D), 3) the difference between mean normalized expressions (column E), 4) the p-value (t-test) (Column F), and 5) Storey q-value (Column G). A positive difference between mean normalized expressions indicate CM-fated over-expression, whereas a negative difference between mean normalized expressions indicate EPDC-fated over-expression.

Table S3D: Description and supporting literature of 91 signature genes in iPSC.

In this table, we describe the known functions of the 91 signature genes identified as differentially expressed between CM-fated and EDPC-fated iPSCs. For each gene, gene name (Column A), ensemble gene id (Column B), Chromosome (column C), gene start (Column D) and end (Column E), the difference between mean normalized expressions (column F), the p-value (t-test) (Column G), and Storey q-value (Column H) are given. Additionally, for each gene functional descriptions (Column J) and PMIDs for supporting literature (Column I) are provided. This table is ordered on the difference between mean normalized expressions.

Table S3E: Regression estimates showing the associations between signature genes and %CM populations.

For each of the 91 signature genes (Columns A, B), linear regression estimate (Column C), standard error (Column D), p-values (Column E, calculated in R as 2*pnorm(estimate / standard error)) and R^2 (Column F) are shown. Columns G-J show the 35 signature genes that L1 norm identified as having significant contribution to cell fate determination: LASSO regression coefficient (Column G), median TPM (Column H), median contribution (Column I), and absolute value of the median contribution to the model (Column J) are shown. Column K shows the seven ELK1 targets as identified in the MSigDB gene set SCGGAAGY_ELK1_02 (Xie et al., 2005).

Table S3F: Associations between genetic variation and differentiation outcome.

For each variant with a GWAS p-value > 10^{-5}, shown are their chromosome (Column A), coordinates (Column B), reference and alternative allele (Columns C, D), dbSNP ID (Column E), allele frequency in iPSCORE (Column F), regression estimate (Column G), standard error (Column H) and p-value (Column I). Regression

estimate, standard error and p-value were calculated using the glm(CM population ~ genotype, family = "quasibinomial") function in R.

Table S4: X chromosome gene dosage plays a role in cardiac differentiation fate. Related to Figures 4 & 5.

Table S4A: GSEA showing functional enrichment of genes differentially expressed between CM-fated and EPDC-fated iPSCs

For each of 9,808 MSigDB gene sets (Column A), GSEA enrichment (Column B), and p-value (Column C) calculated using the R gage package are shown. Storey q-value was used to adjust for multiple testing hypothesis, q-values < 0.05 were considered significant. The analysis (Column E) shows whether the test was performed on all 184 iPSCs ("All iPSC-CVPC samples") or just on the 113 female samples ("Female samples"). Positive GSEA enrichment indicate enrichment for CM-fated iPSCs, whereas negative GSEA enrichment indicate enrichment for EPDC-fated iPSCs.

Table S4B: Table describing results of linear regression analysis to predict factors influencing differentiation potential of iPSC towards CM or EPDC fates. Related to Figure 4.

Factors (Column A) input into the linear regression model. Columns B-E describe the results of the model, including estimate (Column B), standard error (Column C), z-value (Column D), and p-value (Column E).

Table S4C: Allelic imbalance fraction of genes on the X chromosome not in pseudoautosomal regions in females from iPSC samples and from iPSC-CVPC samples. Related to Figure 4.

Gene_id indicates the ensemble gene id (Column A) for X chromosomes genes not in pseaudoautomsomal regions. Columns B-HR show the rna_assay_uuid (Table S1E) of each of the female iPSC (Figure 4D,E) and iPSC-CVPC samples (Figure 4F) for which the allelic imbalance fraction was calculated for each gene.

Table S5: Table describing differentiation outcomes and molecular data ID references from the Yoruba set. Related to Figure 5.

Data from 39 Yoruba iPSC samples (Banovich et al., 2018) (Column A) and their sex (Column B) are given. Outcome (Column C) indicates if the iPSC-CM differentiation was completed or terminated before completion. %cTnT values (Column E), GEO iPSC RNA-seq sample IDs (Column F), and GEO iPSC-CM sample IDs

(Column G) (GEO; GSE89895) are given. Five of the Yoruba iPSCs and two of the iPSC-CM samples did not have RNA-seq data.

SUPPLEMENTARY EXPERIMENTAL PROCEDURES

iPSCORE subject information

Fibroblasts obtained by skin biopsies from the181consented individuals (108 female and 73 male) used in this study were recruited as part of the iPSCORE project (Panopoulos et al., 2017). These individuals included seven monozygotic (MZ) twin pairs, members of 32 families (2-10 members/family) and 71 singletons (i.e. not related with any other individual in this study) and were of diverse ancestries: European (118), Asian (27), Hispanic (12), African American (4), Indian (3), Middle Eastern (2) and mix ethnicity (15). The recruitment of these individuals was approved by the Institutional Review Boards of the University of California, San Diego and The Salk Institute (Project no. 110776ZF). Subject descriptions including subject sex, age, family, ethnicity and cardiac diseases were collected during recruitment (Table S1). While individuals in the iPSCORE Resource were not selected for carrying specific diseases, six individuals had prolonged QT (due to dominant mutations in *KCNQ1* or *KCNH2*), and two members of the same family had Danon disease (due to mutations in *LAMP2*). In addition to fibroblast collection for iPSC reprogramming and differentiation, whole blood samples were obtained for whole genome sequencing.

Whole genome sequencing

As previously described (DeBoever et al., 2017), we generated whole genome sequences from the 181 subjects used for iPSC derivation. Genomic DNA was isolated from whole blood using DNEasy Blood & Tissue Kit (Qiagen) and Qubit quantified. DNA was then sheared using Covaris KE220 instrument and normalized to 1µg, where WGS libraries were prepared using TruSeq Nano DNA HT kit (Illumina) and normalized to 2 - 3.5nM in 6-samples pools. Pooled libraries were clustered and sequenced on the HiSeqX (Illumina; 150 base paired-end) at Human Longevity, Inc. (HLI).

iPSC derivation and somatic mutation analysis

As previously described (Panopoulos et al., 2017), we reprogrammed fibroblast samples from the 181 individuals in this study using non-integrative Cytotune Sendai virus (Life Technologies) (Ban et al., 2011) following the manufacturer's protocol. The 191 iPSCs used in this study (7 subjects had 2 or more clones each; Table S1B) were generated and shown to be pluripotent by analysis of RNA-seq by PluriTest (Muller et al., 2008) and for a subset based on >95% positive double staining for Tra-1-81and SEEA-4 (Panopoulos et al., 2017). The iPSCORE lines have been examined using SNP arrays and shown to have high genomic integrity with no or low numbers of somatic copy-number variants (CNVs) (Panopoulos et al., 2017). Eighteen iPSCORE lines have been analyzed using whole genome sequencing and the mutational profiles shown to

be stable (i.e., not evolving) between passage 12 and later passages, and throughout differentiation into iPSC-CVPCs (D'Antonio et al., 2018).

Large-scale derivation of iPSC-CVPC samples

To generate iPSC-derived cardiovascular progenitors (iPSC-CVPCs) we used a small molecule cardiac differentiation protocol (Lian et al., 2013). The 25-day differentiation protocol consisted of five phases (Figure S1A), the optimizations for each step are described in detail below: 1) *expansion*: we developed the ccEstimate algorithm (Figure S2) to automate the detection of 80% confluency for iPSCs in T150 flasks (Figure S1B,C); 2) *differentiation*: we tested whether increasing the dosage of IWP-2 to induce to inhibit the WNT pathway improved differentiation efficiency and found that 7.5 µM at D3 of the differentiation provided in a single dose for 48 hours results in the most efficient differentiation (Figure S1D, E, Table S1H); 3) *purification*: since fetal cardiomyocytes use lactate as primary energy source and have a higher capacity for lactate uptake than other cell types (Fisher et al., 1981; Werner and Sicard, 1987), we incorporated lactate metabolic selection for five days to improve iPSC-CVPC purity (Tohyama et al., 2013) (Figure S1F); 4) *recovery*: after metabolic selection, iPSC-CVPCs were maintained in cell culture for five days; and 5) *harvest*: we collected iPSC-CVPCs at D25 for downstream molecular assays and cryopreserved live cells.

The 232 attempted differentiations of the 191 iPSC lines (Table S1B) were performed as follows:

Expansion of iPSC: One vial of each iPSC line was thawed into mTeSR1 medium containing 10 µM ROCK Inhibitor (Sigma) and plated on one well of a 6-well plate coated overnight with matrigel. During the expansion phase, all iPSC passaging was performed in mTeSR1 medium containing 5 µM ROCK inhibitor, when cells were visually estimated to be at 80% confluency. The iPSCs were passaged using Versene (Lonza) from one well into three wells of a 6-well plate. Next, the iPSCs were passaged using Versene onto three 10 cm dishes at $2.54x10^4$ per cm^2 density. The iPSCs molonalyer was plated onto three T150 flasks at the density of 3.66 x 10^4 per cm^2 using Accutase (Innovative Cell Technologies Inc.). Prior to expansion with Versene, after thaw iPSCs were passaged 1-2 times using Dispase II (20mg/ml; Gibco/Life technologies). iPSCs were at passage 22.7 ± 4.8 (range 17 to 44) at the monolayer stage (i.e., initiation of differentiation; Table S1B).

Differentiation: At 80% iPSC confluency (measured using ccEstimate, see section below "Estimation of optimal time for initiation of iPSC-CVPCs differentiation using ccEstimate") cell lysates were collected from 32 lines for RNA-seq data generation, where these iPSC and subsequent generated molecular data are referred to as D0 iPSC (Table S1E). After reaching 80% confluency (usually within 4-5 days), differentiation was initiated with the addition of the medium containing RPMI 1960 (gibco-life technologies) with Penicillin – Streptomycin (Gibco/Life Technologies) and B-27 Minus Insulin (Gibco/Life Technologies) (hereafter referred to as RPMI Minus supplemented with 12µM CHIR-99021 (D0). After 24h of exposure to CHIR-99021, medium was changed to RPMI Minus (D1). On D3 medium was changed to 1:1 mix of spent and fresh RPMI Minus supplemented with 7.5µM IWP-2 (Tocris). On D5, after 48h of exposure to IWP-2, the medium was change to RPMI Minus. On D7, medium was changed to RPMI 1960 with Penicillin – Streptomycin (Gibco/Life

Technologies) and B-27 Supplement 50X (hereafter referred to as RPMI Plus) (Gibco/Life Technologies). Between D7 and D13, RPMI Plus medium was changed every 48h.

Purification: On D15 the cells were collected from the flask using Accutase and plated onto fresh T150 flasks at confluency 1-1.3 x 10^6 per cm^2. On D16, cells were washed with PBS without Ca^{2+} and Mg^{2+} (Gibco/Life Technologies) and medium was changed for RPMI 1960 no glucose (Gibco/Life Technologies) supplemented with Non-Essential Amino Acids (Gibco/Life Technologies), L-Glutamine (Gibco/Life Technologies), Penicillin-Streptomycin 10,000U (Gibco/Life Technologies) and 4mM Sodium L-Lactate (Sigma) in 1M HEPES (Gibco/Life Technologies). Medium supplemented with lactate was changed on D17 and D19.

Recovery: On D21 cells were washed with PBS and medium was changed for RPMI Plus. On D23 medium was again changed for RPMI Plus. The first beating cells were usually observed between D7 and D9 and as early as D7 (immediately after the media change) and robust beating was usually observed between D8 and D11. During the lactate selection iPSC-CVPC were beating robustly less than 16 hours after reseeding. For all successfully derived iPSC-CVPCs on D25, total-cell lysate material was collected and frozen for downstream RNA-seq assays.

Harvest: On D25 cells were collected using Accutase and processed for the following molecular material for downstream assays: 1) cell lysates (RNA-Seq); 2) permeabilized cells (ATAC-Seq); 3) live frozen cells (scRNA-seq); 4) cross-linked cells (ChIP-Seq, median number of vials/iPSC line = 3; ~1.0 x 10^7 cells/vial), and 5) dry cell pellets (methylation and protein). RNA-seq was generated from 180 iPSC-CVPC differentiations (149 lines from 139 subjects) that successfully reached D25 (Table S1E).

Estimation of optimal time for initiation of iPSC-CVPCs differentiation using ccEstimate

Heterogeneity of growth rates across different iPSC lines could result in different confluency at the monolayer stage (i.e., faster growing lines will be more confluent) and hence impact differentiation outcome. To reduce the effects of the iPSC lines having different growth rates, we developed an automatic pipeline that analyzes images of monolayer-grown cells, determines their confluency and predicts when cells reach 80% confluency to initiate the differentiation protocol (Figures S1C, S2). Cell confluency estimates (ccEstimate) are performed by first dividing each T150 flask into 10 sections (Figure S1C) and acquiring images for each section every 24 hours after cells are plated as a monolayer. The final image is acquired immediately after treatment with CHIR, which occurs when their confluence is at least 80% (Day 0). The time required for cells to reach 80% confluence is estimated on the basis of the confluence curve derived for each section in each flask. To digitally measure iPSC confluency, ccEstimate performs image analysis using the EBImage package in R (Pau et al., 2010). Images are read using the readImage function.

Confluency measurement data is collected for at least the first three days after plating as monolayer to train a generalized linear model (GLM) using the function glm in R to estimate when cells must be treated with CHIR. Estimation is performed

separately for each flask section and CHIR is added to all three flasks associated to a given line when at least 75% of sections have confluence 80% (Figure S1C).

Using this method, we could start differentiation at the same confluency level for each iPSC sample, thereby reducing or neutralizing the effects of different growth rates. On average, each sample required 4.23 ± 1.12 days to reach 80% confluency (Table S1E). The correlation between the number of days required to reach 80% confluency and the %CM population was -0.05, suggesting that iPSC growth rate does not affect differentiation outcome.

Optimization of IWP-2 concentration by visual estimation of iPSC-CVPCs structure and beating quality

To optimize the IWP-2 concentration, one iPSC line (2_3) was differentiated under four different IWP-2 conditions (Figure S1D, E): 1) 5µM IWP-2 added on D3, 2) 7.5µM IWP-2 added on D3, 3) 5µM IWP-2 added on D3 and D4, or 4) 7.5µM IWP-2 added on D3 and D4. In all four conditions cells were exposed to IWP-2 for 48 hours. At D15 of differentiation, the quality of generated iPSC-CVPC structures and beating were estimated by visual evaluation using two metrics that we established in the lab: 1) structure score; and 2) beat score. Both structure score and beat score were evaluated at 10 spots on each 150T flask that had also been used for digital measurement of cell confluency (Table S1H). Structure score and beat score had 4-point scales where 0 was the lowest and 3 was the highest grade. For structure score 0 = less than 10% of cells were cardiomyocyte-like with thick structures; 1 = 10-25% of cells were cardiomyocyte-like with thick structures; 2 = over 50% of cells were cardiomyocyte-like with thick structures; 3 = over 90% of cells were cardiomyocyte-like with thick structures. For beat score 0 = less than 10% of cells were cardiomyocyte-like beating robustly as a sheet; 1= 10-25% of cells were cardiomyocyte-like beating robustly; 2 = over 50 of cells were cardiomyocyte-like beating robustly; 3 = over 90% of cells were cardiomyocyte-like beating robustly. In cases of uncertainty or intermediate results, cells were assigned a lower grade. Grade 3 was assigned only for the iPSCs with thick, robustly beating sheets of cells.

Comparison of lactate and glucose treated iPSC-CVPCs

To examine the effects of lactate purification, three iPSC-CVPC lines derived from unrelated individuals (2_3, 8_2, and 3_2) were differentiated to D15 (Figure S1F). At D16, medium supplemented with either 4mM Sodium L-Lactate (Sigma) or 2mg/mL D-glucose (Gibco/Life Technologies). Medium was changed on D17 and D19. On D21 cells were washed with PBS and medium was changed for RPMI Plus. Lactate and glucose treated cells were harvested on D25.

Flow cytometry

On D25 of differentiation, $5x10^5$iPSC-CVPCs were permeabilized and blocked in 0.5% BSA, 0.2% TX-100 and 5% goat serum in PBS for 30 minutes at room temperature. Cells were stained with Troponin T, Cardiac Isoform Ab-1, Mouse Monoclonal Antibody (Thermo Scientific, MS-295-P0) at 4°C for 45 minutes, followed by Alexa Fluor 488 secondary antibody (Life Technologies, A11001). Stained cells were acquired using BD FACSCanto II system (BD Biosciences) and analyzed using FlowJo V10.2.

Immunofluorescence analysis of iPSC-CVPCs

Immunofluorescence (IF) was assessed in 5 iPSC-CVPC lines (13_1, 14_2, 29_1, 2_1, and 42_1). Cells for IF were obtained by thawing live frozen iPSC-CVPC harvested on D25 and plating them directly on 0.1% gelatin-coated glass-bottom plates for five days (D30). Cells were then fixed using 4% paraformaldehyde (PFA) in PBS or 20 min at room temperature (RT). Fixed cells were permeabilized for 8 min at RT with 0.1% Triton X-100 in PBS, blocked in 5% bovine serum albumin for 30 min at RT and incubated overnight at 4°C with a primary antibody. Cells were incubated with rabbit polyclonal anti-connexin 43 (Cx43) antibody (Invitrogen, 710700) and with mouse monoclonal anti-sarcomeric alpha-actinin antibody (Sigma, A7811), or with rabbit polyclonal anti-MLC2V (Proteintech, 10906-1-AP) and/or mouse monoclonal anti-MLC2A (Synaptic Systems, 311011). All antibodies are described in Table S1D.

After overnight incubation cells were washed three times with PBS and incubated with appropriate secondary antibodies: donkey anti-rabbit Alexa Fluor 488(Invitrogen, A-21206) and goat anti-mouse Alexa Fluor 568 (Invitrogen, A-11004**)** secondary antibodies for 45 minutes at RT. Cells were washed three times with PBS and nuclei were counterstained with DAPI and mounted. Slides were imaged using Olympus FluoView FV1000 confocal microscope at UCSD Microscopy Core.

Generation of RNA-seq data

For gene expression profiling of iPSCs, we used RNA-seq data from 184 samples (cell lysates were collected between passages 12 to 40, Table S1, dbGaP: phs000924) (DeBoever et al., 2017). For gene expression profiling of iPSC-CVPCs, we generated RNA-seq data from 180 samples at D25 differentiation (Table S1F, dbGaP: phs000924). All RNA-seq samples were generated and analyzed using the same pipeline (DeBoever et al., 2017). Briefly, we isolated total RNA from total-cell lysates using the Quick-RNA™ MiniPrep Kit (Zymo Research) from frozen total-cell lysate, including on-column DNAse treatment steps and eluted in 48 µl RNAse-free water. RNA elutions were run on a Bioanalyzer (Agilent) to determine integrity and all samples had RNA integrity number (RIN) values greater than 9. Illumina Truseq Stranded mRNA libraries were prepared and sequenced on HiSeq4000, to an average of 28 M 125 bp paired-end reads per sample. RNA-Seq reads were aligned using STAR (Dobin et al., 2013) with a splice junction database built from the Gencode v19 gene annotation. RNA-Seq data with percent uniquely mapped reads greater than 70% and percent duplication less than 50% were considered to be good quality. Transcript and gene-based expression values were quantified using the RSEM package (1.2.20) (Li and Dewey, 2011) and normalized to transcript per million bp (TPM).

Generation of scRNA-seq data

Rationale: To capture the full spectrum of heterogeneity among the iPSC-CVPCs, we selected eight samples with variable %cTnT (42.2 to 95.8%). Given the high correlation that we observed between %cTnT and %CM populations in these eight samples, as well as the high correlation between %cTnT and deconvoluted %CM population across all samples with bulk

RNA-seq, we concluded that eight samples were sufficient to capture the full diversity of heterogeneity among the 191 iPSC lines that were differentiated.

Generation: For eight iPSC-CVPCs sample and one H9 ESC line, single cells were captured using the 10x Chromium controller (10x Genomics) according to the manufacturer's specifications and manual (Manual CG00052, Rev C). Cells for each sample were loaded on the individual lane of a Chromium Single Cell A Chip. Libraries were generated using Chromium Single Cell 3' Library Gel Bead Kit v2 (10xGenomics) following manufactures manual. Libraries were sequenced using a custom program (26-8-98 Pair End) on HiSeq 4000. Each library was sequenced on an individual lane. In total we captured 36,839 cells. We retrieved FASTQ files and used CellRanger V2.1 (https://support.10xgenomics.com/) with default parameters using Gencode V19 gene annotation to generate single-cell gene counts for each individual sample.

Processing: To combine the scRNA-seq from each individual sample, we used *cellranger aggr* and obtained a total of 36,839 cells from 8 iPSC-CVPCs and 1 ESC sample. We removed 1,934 cells because they were not in G0 phase, as they expressed the proliferation marker MKI67 (Scholzen and Gerdes, 2000) at high levels (UMI > 2, Figure S4A-D). We also removed doublets (i.e. sequenced droplets containing more than one cell)(Kang et al., 2018)by visual inspection of the t-SNE plots (Figure S4). There were 34,905 cells remaining after proliferating cells and doublets were removed. K-means clustering was performed on the 34,905 cells using k values 3, 4, and 9 (FigureS4E-G). k = 3 was determined to be the most suitable value, as visual inspection of the principal component analysis showed 3 distinct clusters (Figure S4E). The clustering shown both in the heatmap and in the UMAP plots (Figure 1G, 1H, 1J) was performed on the top 10 principal components calculated based on the expression levels of each single cell, according to the CellRanger pipeline.

Differential expression: Differential expression across the three scRNA-seq clusters was performed by comparing the distribution of unique molecular identifiers (UMI) for a given gene from all the cells specific to one cluster (k-means; k = 3) with all the cells specific to the other two clusters using edgeR asymptotic beta test (Robinson and Smyth, 2008) (Table S2). Differentially genes that had a total UMI ≥ 1 and FDR < 0.05 were considered to be significantly overexpressed in a given cluster. For visualization of gene expression in the t-SNE plots, transcript levels for each gene were normalized using the *calcNormFactors* function in edgeR (Robinson et al., 2010).

CIBERSORT

The expression levels of the top 50 genes overexpressed in each of the three cell populations (total 150 genes), with nominal p-value < 1.0×10^{-13} and mean UMI > 1 (Table S1G), were used as input for CIBERSORT (Newman et al., 2015) to calculate the relative distribution of the three cell populations for all the 180 iPSC-CVPC samples at D25. CIBERSORT (https://cibersort.stanford.edu/) was run with default parameters using the TPM values for the 150 genes in all 180 iPSC-CVPC samples.

Characterizing transcriptional similarities of iPSCs, iPSC-CVPCs and GTEx adult tissues by principal component analysis

We performed principal component analysis (PCA) on RNA-seq using R prcomp function on 184 iPSCs, 180 iPSC-CVPCs and 1,072 RNA-seq samples from GTEx, including 303 left ventricle samples, 297 atrial appendage samples, 173 coronary artery samples and 299 aorta samples.

Determining optimal CM:EPDC ratio estimates from CIBERSORT to define iPSCs cardiac fates

For each iPSC line that had more than one iPSC-CVPC differentiation, we used the sample with the highest Population 1 fraction. To obtain the optimal threshold, we used the RNA-seq data to conduct a series of differential expression analyses on 15,228 autosomal genes in the 184 iPSC lines (147 completed and 37 terminated) with RNA-seq data considering the ratio of population frequencies in the corresponding derived iPSC-CVPCs (0:100, 10:90, 20:80, 30:70, 40:60, 50:50, 60:40, 70:30, 80:20 and 90:10, Table S3A-B). For example, for the 90:10 ratio we compared gene expression in the 60 iPSCs that differentiated into iPSC-CVPCs with >= 90% Population 1 to 124 iPSCs that differentiated into iPSC-CVPCs with less than 90% Population 1. iPSC lines that that had terminated differentiations were assigned a CM:EPDC ratio of 0:100. To calculate differential expression at each threshold between CM-fated iPSCs and EPDC-fated iPSCs, we first retained all genes with TPM ≥ 2 in at least 10 samples and then transformed the RNA-seq TPM data to standard normal distributions by quantile normalization using the function normalize.quantiles from R package preprocessCore (Bolstad et al., 2003). Quantile normalized expression levels were then corrected for the first 10 factors calculated by PEER (Stegle et al., 2012). To remove any biases resulting from the fact that the ratio of male to female iPSCs was 71:113, we identified 242 genes that were significantly differentially expressed between female and male iPSCs (Storey q-value < 0.1, t-test) and removed them from further analyses (Table S3A-B). Across the ten thresholds, there were 116 differentially expressed autosomal genes (t-test, Table S3A-B). Considering these 116 genes, the 30:70 (CM:EPDC) ratio resulted in the highest number of differentially expressed genes (84 genes with Storey q-value < 0.1, t-test, Figure 3, Table S3C), which is substantially greater than random expectation (Table S9). Thus, we grouped the 184 iPSC lines into: 1) those that have CM fates, i.e. produced iPSC-CVPC with >= 30% Population 1 (125 lines), and 2) those that have EPDC fates, i.e. produced iPSC-CVPC with > 70% Population 2 (22 lines differentiated to D25 and 37 terminated lines). We did not observe significant expression differences between the 22 iPSCs that were designated EPDC-fated because their corresponding iPSC-CVPCs had >70% Population 2 and the 37 iPSCs designated EPDC-fated because their differentiations were terminated before D25 (Table S3A-B).

Comparing the number of differentially expressed genes with random expectation

To determine if the number of significantly differentially expressed genes was higher than expected by chance, we shuffled the assignments of the 184 iPSC RNA-seq samples to differentiation fate (125 CM and 59 EPDC) 100 times. For each shuffle, we performed differential expression analysis and obtained the number of genes that were significantly differentially

expressed. In Figure S9 we show a QQ plot that demonstrates that the observed p-value distribution was substantially different than random expectation.

Contribution of 91 signature genes in iPSCs to determination of cardiac fate

Individual contributions: For each of the 91 signature genes, we built a generalized linear model (GLM) with the expression of the gene as input and the differentiation outcome (e.g. % Population 1) as output using the LinearRegression function from sklearn. To model the continuous property of the % Population 1 distributions, but maintain their boundary from 0-100, we used a logit link function to transform measurements of % cardiomyocyte to ln(OR) of % Population 1, calculated as ln(% Population 1/ (1 - % Population 1) and capped the percentages at 0.99 and 0.01 to avoid infinite or undefined odds ratios. For each gene, the percent of variance explained is defined as the model's R^2.

Cumulative impact: To understand the cumulative contribution of all 91 signature genes on cardiac differentiation fate, we built a generalized linear model (GLM) with an L1 norm penalty (ie LASSO) using the expression of all 91 genes as input and the differentiation outcome (e.g. % Population 1) as output using the LassoLarsCV function from sci-it learn. v0.19.1 To model the continuous property of the % Population 1 distributions, but maintain their boundary from 0-100, we used a logit link function to transform measurements of % cardiomyocyte to ln(OR) of % CM population, calculated as ln(% CM/ (1 - % CM) and capped the percentages at 0.99 and 0.01 to avoid infinite or undefined odds ratios. To avoid overfitting the model, we used a 10-fold cross validation implemented in sci-kit learn v0.19.1 with 10,000 max iterations (Pedregosa et al., 2011). The average R^2, as reported by sci-it learn, is calculated by finding the R^2 for each of the individual folds (i.e., 10 R^2s), and averaging these values to find how well the model performs across different data subsets.

Detecting associations between genetic background and differentiation outcome

We obtained genotypes for 8,620,159 biallelic SNPs and short indels with allelic frequency >5% in the iPSCORE collection (Panopoulos et al., 2017). Genotypes were obtained for each SNP in all individuals using *bcftools view* (Li, 2011). Linear regression was used to calculate the associations between the genotype of each variant and differentiation outcome (% CM population in the iPSC-CVPCs), using passage at monolayer and sex as covariates.

To test if differentiations of different iPSC clones from the same individual or same twin pair were more likely to produce similar outcomes than iPSC clones from individuals with different genetic backgrounds, we first calculated the absolute difference in %CM between each pair of 180 iPSC-CVPCs. Next, we tested if the distributions between the three groups were different using Mann-Whitney U test (Figure S11B).

Gene set enrichment analysis using the MSigDB collection

We performed gene set enrichment analysis (GSEA) using the R *gage* package (V 2.20.1)(Luo et al., 2009) on all MSigDB gene sets (Liberzon et al., 2011; Subramanian et al., 2005) from 8 collections, including Hallmark gene sets (H), positional

gene sets (C1), curated gene sets (C2), motif gene sets (C3), computational gene sets (C4), Gene Ontology (GO, C5), oncogenic signatures (C6), and immunologic signatures (C7). FDR correction was performed independently for each collection. The normalized mean expression difference between iPSCs that differentiated to CMs and iPSCs that differentiated to EPDCs (Table S3C) was used as input for GSEA. Gene lists that were significant after multiple testing correction (Storey q-value < 0.05) were considered significant.

Associations between iPSC and subject features and differentiation outcome

A generalized linear model (GLM) was built in R using age, sex, ethnicity, age, and passage of the iPSCs at D0 of differentiation as input and differentiation outcome as output (0 = EPDCs; and 1 = CMs). The model was built using the function glm (outcome ~ age + sex + ethnicity + passage, family=binomial(link='logit')).

Identifying X chromosome inactivation in female iPSCs and iPSC-CVPCs

To analyze X chromosome inactivation, we used 113 female iPSCs, of which 87 where CM-fated and 26 were EPDC-fated. To call allele specific effects (ASE) in RNA-Seq from iPSC and iPSC-CVPCs, we used the method previously described in DeBoever *et al*.(DeBoever et al., 2017). Genes lying in X chromosome pseudoautosomal (PAR) regions (PAR1: 60001-2699520, PAR2: 154931044 – 155260560) were removed from the analysis. We defined the strength of ASE for each gene as the fraction of RNA transcripts that were estimated to originate from the allele with higher expression (referred to as allelic imbalance fraction, AIF).

Validation of findings in Yoruba iPSC set

Generation of iPSCs: The Yoruba iPSCs in the Banovich*et al.* study (Banovich et al., 2018) were generated from lymphoblastoid cell lines (LCLs) using an episomal reprogramming strategy. Briefly, this included transfecting LCLs with the episomal plasmids and then culturing for seven days in hESC media (DMEM/F12 supplemented with 20% KOSR, 0.1 mM NEAA, 2mM GlutaMAX, 1% Pen/Strep, 0.1# 2-Mercaptoethanol, 25ng/µl of bFGF, and 0.5mM NaB). On day eight, the transfected cells were plated in a 6-well plates. After four days, NaB was removed from the hESC media. Colonies were observed within 21 days and passaging continued for an additional 10 weeks (1 passage / week), where cells were collected for cryopreservation. Material collected for RNA-seq of the iPSC were collected after an additional minimum of three passages.

Differentiation protocol: The Yoruba iPSC-CM derivation (Banovich et al., 2018) was performed using a small molecular method similar to iPSCORE iPSC differentiation protocol (see above: Large-scale iPSC-CVPC deviation). Briefly, 39 iPSCs were expanded until 70-100% confluency (three to five days). On D0, differentiation was initiated by the supplementation of media with 12µM of GSK3 inhibitor CHIR-99021 for WNT pathway activation. On D3 of differentiation, 2µM of Wnt-C59 was added (PORCN inhibitor). On D5 of differentiation, Wnt-C59 was removed from culturing media and differentiating cells were grown with regular media exchanges from D5 to D14. On D14, D16, and D18

cultures were exposed to 5mM Sodium L-lactate for cardiomyocyte purification. On D20-D25, differentiating cells were exposed to 1.7 mg/mL galactose daily to force aerobic metabolism and thus aid in cardiomyocyte maturation. On D25-D27, cells were incubated at physiological oxygen levels (10%). On D27 cells were electrically stimulated with 6.6 V/cm, 2ms and 1Hz for further aid in cardiomyocyte maturation. Finally, iPSC-CMs were harvested on D31 or D32. Purity of iPSC-CM Yoruba lines were measured by cTnT marker and flow cytometry. Out of the 39 iPSCs for which differentiation was attempted, 15 lines successfully generated iPSC-CMs and 24 were terminated on or before day 10 due to the fact that they did not form a beating syncytium (Table S5).

RNA-seq: We downloaded RNA-seq for 34 of the Yoruba iPSC (14 successful iPSC and 20 terminated iPSC, five iPSCs did not have RNA-seq) and 13 iPSC-CM samples (two iPSC-CMs did not have RNA-seq) from Gene Expression Omnibus (GEO; GSE89895) (Banovich et al., 2018), as well as 297 samples from 19 distinct iPSCs in a timecourse experiment (day 0-15) performed on the same Yoruba iPSC samples (Strober et al., 2019). These Yoruba RNA-seq data were generated from Illumina TrueSeq prepared libraries and sequenced at 50 bp single-end reads on an Illumina 2500. As iPSCORE RNA-seq was 125 bp paired-end reads, for comparative analyses, we trimmed all iPSCORE iPSC and iPSC-CM data to 50 bp and treated the paired-end reads as single-end reads. Both iPSCORE and Yoruba 50 bp RNA-seq was then processed as described above (Methods: Generation of RNA-seq data). Briefly, RNA-seq was aligned using STAR, then gene expression was quantified using the RSEM package and normalized to TPM.

Estimation of cellular composition: The RNA-seq for the 13 Yoruba iPSC-CMs and from all timecourse time points were analyzed using CIBERSORT similar to the iPSCORE samples (see CIBERSORT section above). Briefly, the TPM values of the 150 overexpressed genes (50 from each of the three single cell populations; Table S2) were used as input to CIBERSORT to calculate the relative distribution of the three populations.

Testing if iPSCORE differentially expressed genes with nominal significant expression differences in the same direction (e.g. over-expressed or down regulated) in the Yoruba iPSCs is greater than random expectation: Of 13,704 genes expressed both in the iPSCORE and Yoruba iPSCs, we obtained 6,909 for which the average normalized expression differences had either the same positive (CM fate/successful differentiation) or negative (EPDC fate/terminated differentiation) direction. The 6,909 genes included 47 of the 91 iPSCORE signature genes. We found that 466 (6.7%) of the 6,909 genes were nominally significant for being differentially expressed between the 14 successful and 20 terminated differentiations in the Yoruba samples, while 8 of the 47 iPSCORE differentially expressed genes (17.0%) had a nominal $p < 0.05$. This analysis shows that the 91 iPSCORE signature genes are 2.5 times more likely than expected (17.0% vs. 6.7%, $p = 0.012$, Fisher's exact test) to be differentially expressed in the Yoruba samples based on cardiac differentiation fate.

REFERENCES

Ban, H., Nishishita, N., Fusaki, N., Tabata, T., Saeki, K., Shikamura, M., Takada, N., Inoue, M., Hasegawa, M., Kawamata, S., *et al.* (2011). Efficient generation of transgene-free human induced pluripotent stem cells (iPSCs) by temperature-sensitive Sendai virus vectors. Proc Natl Acad Sci U S A *108*, 14234-14239.

Banovich, N.E., Li, Y.I., Raj, A., Ward, M.C., Greenside, P., Calderon, D., Tung, P.Y., Burnett, J.E., Myrthil, M., Thomas, S.M., *et al.* (2018). Impact of regulatory variation across human iPSCs and differentiated cells. Genome Res *28*, 122-131.

Bolstad, B.M., Irizarry, R.A., Astrand, M., and Speed, T.P. (2003). A comparison of normalization methods for high density oligonucleotide array data based on variance and bias. Bioinformatics *19*, 185-193.

Burridge, P.W., Matsa, E., Shukla, P., Lin, Z.C., Churko, J.M., Ebert, A.D., Lan, F., Diecke, S., Huber, B., Mordwinkin, N.M., *et al.* (2014). Chemically defined generation of human cardiomyocytes. Nat Methods *11*, 855-860.

D'Antonio, M., Benaglio, P., Jakubosky, D., Greenwald, W.W., Matsui, H., Donovan, M.K.R., Li, H., Smith, E.N., D'Antonio-Chronowska, A., and Frazer, K.A. (2018). Insights into the Mutational Burden of Human Induced Pluripotent Stem Cells from an Integrative Multi-Omics Approach. Cell Rep *24*, 883-894.

DeBoever, C., Li, H., Jakubosky, D., Benaglio, P., Reyna, J., Olson, K.M., Huang, H., Biggs, W., Sandoval, E., D'Antonio, M., *et al.* (2017). Large-Scale Profiling Reveals the Influence of Genetic Variation on Gene Expression in Human Induced Pluripotent Stem Cells. Cell Stem Cell *20*, 533-546 e537.

Dobin, A., Davis, C.A., Schlesinger, F., Drenkow, J., Zaleski, C., Jha, S., Batut, P., Chaisson, M., and Gingeras, T.R. (2013). STAR: ultrafast universal RNA-seq aligner. Bioinformatics *29*, 15-21.

Fisher, D.J., Heymann, M.A., and Rudolph, A.M. (1981). Myocardial consumption of oxygen and carbohydrates in newborn sheep. Pediatr Res *15*, 843-846.

Kadari, A., Mekala, S., Wagner, N., Malan, D., Koth, J., Doll, K., Stappert, L., Eckert, D., Peitz, M., Matthes, J., *et al.* (2015). Robust Generation of Cardiomyocytes from Human iPS Cells Requires Precise Modulation of BMP and WNT Signaling. Stem Cell Rev *11*, 560-569.

Kang, H.M., Subramaniam, M., Targ, S., Nguyen, M., Maliskova, L., McCarthy, E., Wan, E., Wong, S., Byrnes, L., Lanata, C.M., *et al.* (2018). Multiplexed droplet single-cell RNA-sequencing using natural genetic variation. Nat Biotechnol *36*, 89-94.

Li, B., and Dewey, C.N. (2011). RSEM: accurate transcript quantification from RNA-Seq data with or without a reference genome. BMC Bioinformatics *12*, 323.

Li, H. (2011). A statistical framework for SNP calling, mutation discovery, association mapping and population genetical parameter estimation from sequencing data. Bioinformatics *27*, 2987-2993.

Lian, X., Zhang, J., Azarin, S.M., Zhu, K., Hazeltine, L.B., Bao, X., Hsiao, C., Kamp, T.J., and Palecek, S.P. (2013). Directed cardiomyocyte differentiation from human pluripotent stem cells by modulating Wnt/beta-catenin signaling under fully defined conditions. Nat Protoc *8*, 162-175.

Liberzon, A., Subramanian, A., Pinchback, R., Thorvaldsdottir, H., Tamayo, P., and Mesirov, J.P. (2011). Molecular signatures database (MSigDB) 3.0. Bioinformatics *27*, 1739-1740.

Luo, W., Friedman, M.S., Shedden, K., Hankenson, K.D., and Woolf, P.J. (2009). GAGE: generally applicable gene set enrichment for pathway analysis. BMC Bioinformatics *10*, 161.

Muller, F.J., Brandl, B., and Loring, J.F. (2008). Assessment of human pluripotent stem cells with PluriTest. In StemBook (Cambridge (MA)).

Newman, A.M., Liu, C.L., Green, M.R., Gentles, A.J., Feng, W., Xu, Y., Hoang, C.D., Diehn, M., and Alizadeh, A.A. (2015). Robust enumeration of cell subsets from tissue expression profiles. Nat Methods *12*, 453-457.

Panopoulos, A.D., D'Antonio, M., Benaglio, P., Williams, R., Hashem, S.I., Schuldt, B.M., DeBoever, C., Arias, A.D., Garcia, M., Nelson, B.C., *et al.* (2017). iPSCORE: A Resource of 222 iPSC Lines Enabling Functional Characterization of Genetic Variation across a Variety of Cell Types. Stem Cell Reports *8*, 1086-1100.

Pau, G., Fuchs, F., Sklyar, O., Boutros, M., and Huber, W. (2010). EBImage--an R package for image processing with applications to cellular phenotypes. Bioinformatics *26*, 979-981.

Pedregosa, F., Varoquaux, G., Gramfort, A., Michel, V., Thirion, B., Grisel, O., Blondel, M., Prettenhofer, P., Weiss, R., Dubourg, V., *et al.* (2011). Scikit-learn: Machine Learning in Python. J Mach Learn Res *12,* 2825–2830.

Robinson, M.D., McCarthy, D.J., and Smyth, G.K. (2010). edgeR: a Bioconductor package for differential expression analysis of digital gene expression data. Bioinformatics *26*, 139-140.

Robinson, M.D., and Smyth, G.K. (2008). Small-sample estimation of negative binomial dispersion, with applications to SAGE data. Biostatistics *9*, 321-332.

Scholzen, T., and Gerdes, J. (2000). The Ki-67 protein: from the known and the unknown. J Cell Physiol *182*, 311-322.
Stegle, O., Parts, L., Piipari, M., Winn, J., and Durbin, R. (2012). Using probabilistic estimation of expression residuals (PEER) to obtain increased power and interpretability of gene expression analyses. Nat Protoc *7*, 500-507.
Strober, B.J., Elorbany, R., Rhodes, K., Krishnan, N., Tayeb, K., Battle, A., and Gilad, Y. (2019). Dynamic genetic regulation of gene expression during cellular differentiation. Science *364*, 1287-1290.
Subramanian, A., Tamayo, P., Mootha, V.K., Mukherjee, S., Ebert, B.L., Gillette, M.A., Paulovich, A., Pomeroy, S.L., Golub, T.R., Lander, E.S., *et al.* (2005). Gene set enrichment analysis: a knowledge-based approach for interpreting genome-wide expression profiles. Proc Natl Acad Sci U S A *102*, 15545-15550.
Tohyama, S., Hattori, F., Sano, M., Hishiki, T., Nagahata, Y., Matsuura, T., Hashimoto, H., Suzuki, T., Yamashita, H., Satoh, Y., *et al.* (2013). Distinct metabolic flow enables large-scale purification of mouse and human pluripotent stem cell-derived cardiomyocytes. Cell Stem Cell *12*, 127-137.
Werner, J.C., and Sicard, R.E. (1987). Lactate metabolism of isolated, perfused fetal, and newborn pig hearts. Pediatr Res *22*, 552-556.
Xie, X., Lu, J., Kulbokas, E.J., Golub, T.R., Mootha, V., Lindblad-Toh, K., Lander, E.S., and Kellis, M. (2005). Systematic discovery of regulatory motifs in human promoters and 3' UTRs by comparison of several mammals. Nature *434*, 338-345.
