# Supplementary material for: Association of Human iPSC Gene Signatures and X Chromosome Dosage with Two Distinct Cardiac Differentiation Trajectories
Source: Stem Cell Reports. 2019 Oct 24;13(5):924–38. doi: 10.1016/j.stemcr.2019.09.011 (PMC6895695; doi:10.1016/j.stemcr.2019.09.011)
Supplement: Document S2. Article plus Supplemental Information [file mmc7.pdf]

Stem Cell Reports
Article

ISSCR

OPEN ACCESS

Association of Human iPSC Gene Signatures and X Chromosome Dosage with Two Distinct Cardiac Differentiation Trajectories

Agnieszka D'Antonio-Chronowska,[1,6] Margaret K.R. Donovan,[2,6] William W. Young Greenwald,[2] Jennifer Phuong Nguyen,[2] Kyohei Fujita,[1] Sherin Hashem,[3] Hiroko Matsui,[1] Francesca Soncin,[4] Mana Parast,[4] Michelle C. Ward,[5] Florence Coulet,[1] Erin N. Smith,[1] Eric Adler,[3] Matteo D'Antonio,[1,*] and Kelly A. Frazer[1,*]

[1]Department of Pediatrics, UC San Diego, La Jolla, CA 92093, USA
[2]Bioinformatics and Systems Biology Graduate Program, UC San Diego, La Jolla, CA 92093, USA
[3]Division of Cardiology, Department of Medicine, UC San Diego, La Jolla, CA 92093, USA
[4]Department of Pathology, UC San Diego, La Jolla, CA 92093, USA
[5]Department of Medicine, University of Chicago, Chicago, IL 60637, USA
[6]Co-first author
*Correspondence: mdantonio@ucsd.edu (M.D.), kafrazer@ucsd.edu (K.A.F.)
https://doi.org/10.1016/j.stemcr.2019.09.011

SUMMARY

Despite the importance of understanding how variability across induced pluripotent stem cell (iPSC) lines due to non-genetic factors (clone and passage) influences their differentiation outcome, large-scale studies capable of addressing this question have not yet been conducted. Here, we differentiated 191 iPSC lines to generate iPSC-derived cardiovascular progenitor cells (iPSC-CVPCs). We observed cellular heterogeneity across the iPSC-CVPC samples due to varying fractions of two cell types: cardiomyocytes (CMs) and epicardium-derived cells (EPDCs). Comparing the transcriptomes of CM-fated and EPDC-fated iPSCs, we discovered that 91 signature genes and X chromosome dosage differences are associated with these two distinct cardiac developmental trajectories. In an independent set of 39 iPSCs differentiated into CMs, we confirmed that sex and transcriptional differences affect cardiac-fate outcome. Our study provides novel insights into how iPSC transcriptional and X chromosome gene dosage differences influence their response to differentiation stimuli and, hence, cardiac cell fate.

INTRODUCTION

Variability in human induced pluripotent stem cell (iPSC) lines compromises their utility for regenerative medicine and as a model system for genetic studies. This variability affects iPSC differentiation outcome and, despite using standardized differentiation protocols, results in the generation of samples with cellular heterogeneity (i.e., multiple cell types are present within a given sample and the proportions of cell types vary across samples). Previous large-scale quantitative trait loci studies in iPSCs (DeBoever et al., 2017; Kilpinen et al., 2017) have shown that genetic variation accounts for the majority of expression differences between iPSC lines, but non-genetic (i.e., clonality and passage) factors also contribute to these differences (Panopoulos et al., 2017b). Understanding how non-genetic transcriptional differences between iPSC lines affect their differentiation outcome is necessary to improve the ability to generate cell types of interest.

Well-established small-molecule protocols for generating iPSC-derived cardiovascular progenitor cells (iPSC-CVPCs) (Lian et al., 2013) produce fetal-like cardiomyocytes, which can undergo further specification as cells mature in culture into various cardiac subtypes (atrial, ventricular, or nodal) (Burridge et al., 2014). Based on variable cardiac troponin T (cTnT) staining, the derived samples are known to display cellular heterogeneity (Dubois et al., 2011; Witty et al., 2014), but the origin of the cTnT-negative non-myocyte cells, and whether the same or different non-myocyte cell types are consistently derived alongside cTnT-positive myocytes across samples, have not previously been investigated. The differentiation protocol is dependent on manipulation of WNT signaling, initially through activation of the pathway by GSK3 inhibition, followed by inhibition of the pathway by Porcupine (*PORCN*) inhibition (Mo et al., 2013; Wang et al., 2013). An in-depth analysis of the outcomes of independent differentiations of hundreds of iPSC lines with different genetic backgrounds could provide insights into the origins of the non-myocyte cells, as well as the extent to which non-genetic transcriptional differences between iPSC lines contribute to the iPSC-CVPC cellular heterogeneity.

Here, we used a highly standardized and systematic approach to conduct 232 directed differentiations of 191 iPSC lines into iPSC-CVPCs. We characterized the cellular heterogeneity of the iPSC-CVPC samples and showed that only two distinct cell types were present, cardiomyocytes (CMs) and epicardium-derived cells (EPDCs), which varied in proportion across samples. As differentiation protocols to derive iPSC-CMs and iPSC-EPDCs primarily differ by a step involving WNT inhibition to derive the former but not the latter (Bao et al., 2016), we hypothesized that

This is an open access article under the CC BY license (http://creativecommons.org/licenses/by/4.0/).

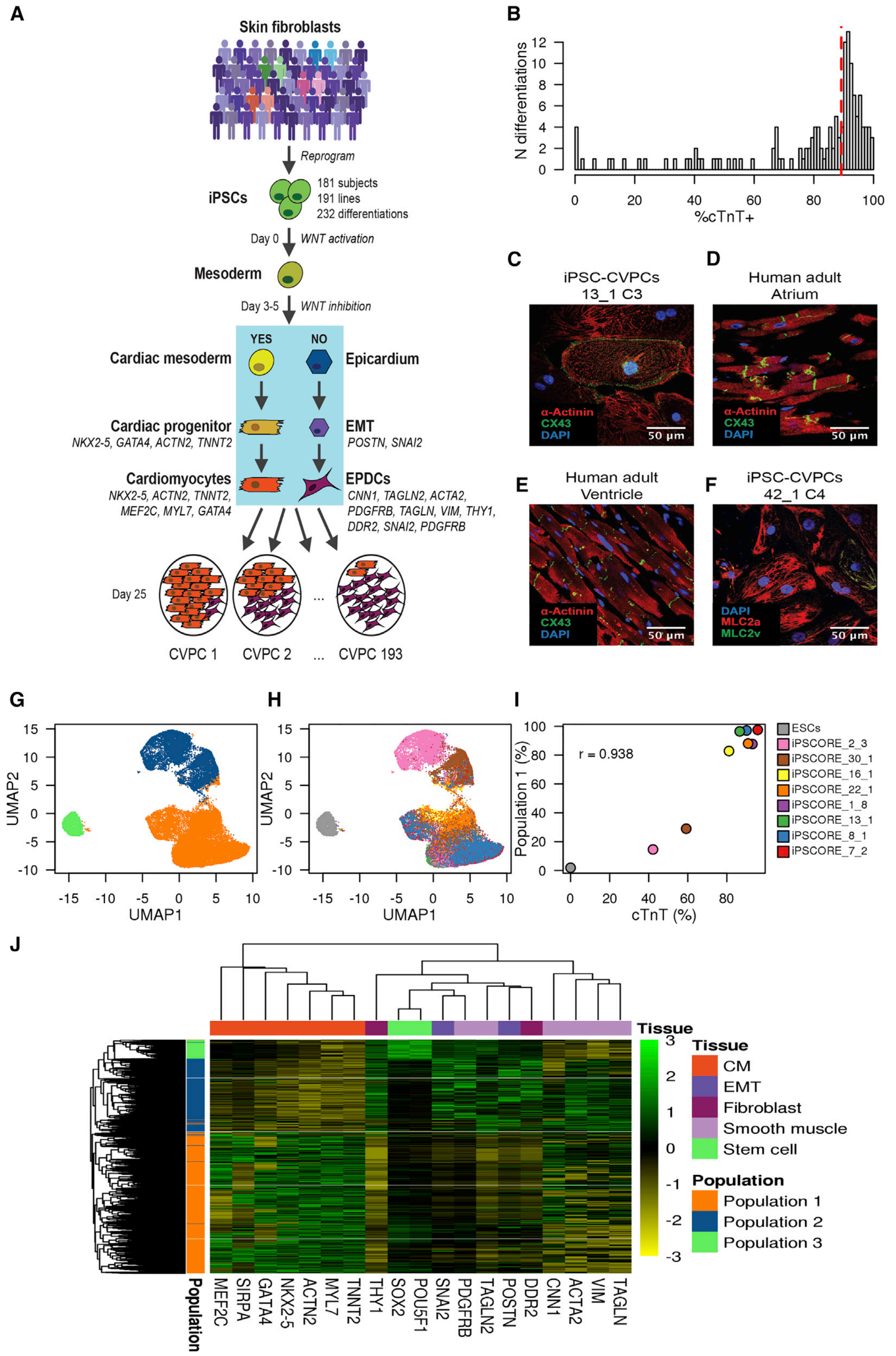

(legend on next page)

the observed cellular heterogeneity could result from suboptimal WNT inhibition in subsets of cells across iPSC lines. To test this hypothesis, we analyzed transcriptional differences between iPSC lines that differentiated into CMs and those that differentiated into EPDCs (e.g., iPSCs with a CM fate or EPDC fate) and discovered 91 signature genes associated with these two distinct cardiac differentiation trajectories. These signature genes are involved in differentiation, including the Wnt/β-catenin pathway, muscle differentiation or cardiac-related functions, and the transition of epicardial cells to EPDCs by epithelial-mesenchymal transition (EMT). While the proportion of variance explained by each of the signature genes varied over three orders of magnitude, altogether they captured approximately half of the total variance underlying iPSC fate determination. Additionally, we show that variability in X chromosome gene dosage ($X_{active}X_{active}$ versus X_{active} $X_{inactive}$ versus XY) across iPSCs plays a role in cardiac-fate determination. The association with X chromosome gene dosage could in part be due to higher expression in CM-fated iPSCs of chrXp11 genes, which encodes *ELK1* and *PORCN*. Transcriptomic analysis of an independent set of 39 iPSCs differentiated to the cardiac lineage using a similar small-molecule protocol (Banovich et al., 2018) confirmed our findings.

RESULTS

iPSC-CVPCs Show Cellular Heterogeneity across Samples

To gain insights into molecular mechanisms that could influence variability in human iPSC differentiation outcome, we employed a highly systematic approach (Figure S1) to differentiate 191 pluripotent lines from 181 iPSCORE individuals (Figure 1A and Table S1A) into iPSC-derived cardiovascular progenitor cells (iPSC-CVPCs). We used a small-molecule cardiac differentiation protocol used to derive cardiomyocytes (Lian et al., 2015) followed on day 15 by lactate selection to obtain pure cardiac cells (Tohyama et al., 2013). In total, we conducted 232 differentiations, of which 193 (83.2%, from 154 lines derived from 144 subjects) were completed, i.e., reached day 25 of differentiation, while 39 (from 37 lines derived from 37 subjects) were terminated prior to day 25 because they did not form a syncytial beating monolayer (Tables S1B and S1C). The completed iPSC-CVPCs at day 25 on average had a high fraction of cells that stained positive for cTnT (%cTnT, median = 89.2%; Figure 1B) and were positive by immunofluorescence for cardiac markers (Figures 1C–1F and S1); however, 15 lines had %cTnT <40%, indicating that despite lactate selection, there was substantial cellular heterogeneity within and across samples.

Subset of Cells Shows Differential Response to WNT Inhibition during Differentiation

To examine the cellular heterogeneity in the iPSC-CVPCs, we performed single-cell RNA sequencing (scRNA-seq) on eight samples with varying %cTnT values (42.2%–95.8%, Tables S1E and S1F) and combined these data with scRNA-seq from the H9 embryonic stem cell (ESC) line (total of 34,905 cells). We detected three distinct cell populations: (1) population 1, 21,056 cells (60.3%); (2) population 2, 11,044 cells (31.6%); and (3) population 3, 2,805 cells (8.1%, Figures 1G and S1; Table S1G). While populations 1 and 2 comprised the eight iPSC-derived samples, population 3 almost exclusively included ESCs (97.7% of the 2,870 ESCs, Figures 1H and S2). The relative proportions of cells that each of the iPSC-CVPC samples contributed to population 1 versus population 2 was strongly correlated with its % cTnT value ($r = 0.938$, $p = 1.89 \times 10^{-4}$, t test; Figure 1I), suggesting that population 1 was cardiomyocytes (CMs).

As CMs and epicardium lineage cells could both survive lactate purification (Iyer et al., 2015; Tohyama et al., 2013), we investigated whether the non-myocyte cells composing population 2 were iPSC-EPDCs. We examined the expression levels of 17 marker genes (Figure 1J) specific for either CMs or EPDCs (including smooth muscle, fibroblasts, and genes involved in EMT) and two marker genes for stem cells. Consistent with having a high number of

Figure 1. Characterization of Cellular Heterogeneity in iPSC-CVPC Samples

(A) Overview of the study design. Skin fibroblasts from 181 subjects were reprogrammed to iPSCs and differentiated to iPSC-CVPCs (191 lines, 232 differentiations). After WNT pathway activation at day 0 and its inactivation by IWP-2 at days 3–5, cells differentiate to CMs if WNT signaling is successfully inhibited. If WNT signaling is not sufficiently inhibited, cells differentiate to EPDCs. Of the 232 differentiations, 193 were completed (day 25), and we observed that different CVPC samples had different proportions of CMs and EPDCs.

(B) Distribution of %cTnT. Dashed red line represents the median value.

(C–E) Immunofluorescence staining of (C) iPSC-CVPCs, (D) human atrium, and (E) ventricle with markers DAPI, ACTN1, and CX43.

(F–H) Immunofluorescence staining of iPSC-CVPCs with markers DAPI, MLC2a^{+} and MLC2v^{+}, and MLC2v^{+}MLC2a^{+} (F). scRNA-seq UMAP plots showing (G) the presence of three populations: CMs (orange), EPDCs (blue), and ESCs (green), and (H) the distribution of the nine analyzed samples (eight iPSC-CVPC lines and one ESC line) across the three clusters.

(I) Scatterplot showing the correlation between the %cTnT and the fraction of cells in population 1 (CMs) for each of the nine samples.

(J) Heatmap showing across all 34,905 single cells the expression markers for stem cells, CMs, EMT, fibroblasts, and smooth muscle.

See also Figures S1 and S2.

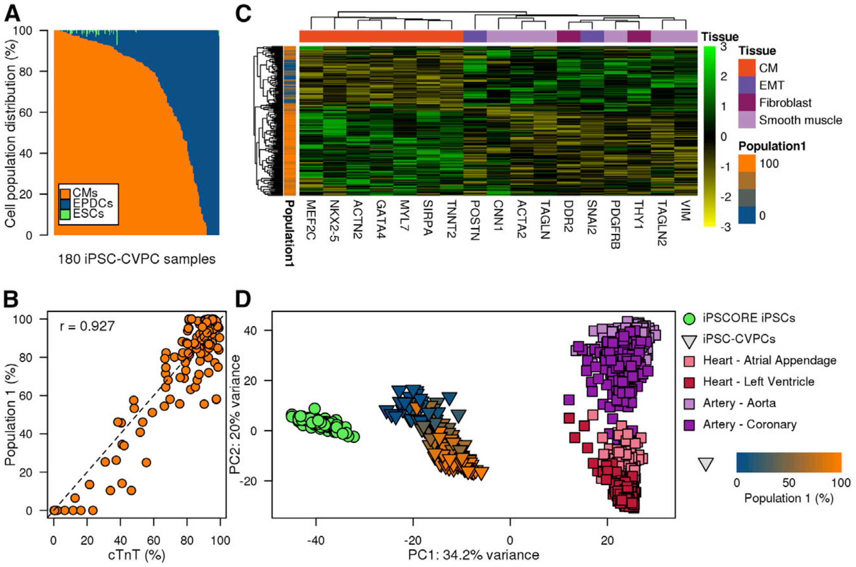

Figure 2. Transcriptomic Features of 180 iPSC-CVPC Samples

(A) Relative distributions of cell populations estimated using CIBERSORT across 180 iPSC-CVPC samples.

(B) Scatterplot showing the correlation between %cTnT (x axis) and the fraction of population 1 in the iPSC-CVPCs calculated using CIBERSORT (y axis).

(C) Heatmap showing the expression levels of CM and EPDC marker genes (Figure 1J) in 180 iPSC-CVPC samples. Samples are colored based on their fraction of population 1.

(D) PCA of the 1,000 genes with highest variability from 184 iPSC samples, 180 iPSC-CVPCs (triangles colored according to their percentage of population 1), and samples from GTEx (squares—left ventricle, right ventricle, coronary artery, and aorta).

cTnT-positive cells, population 1 expressed high levels of CM-specific genes while population 2 expressed high levels of EPDC-specific genes, and population 3 expressed high levels of the stem cell markers *POU5F1* and *SOX2* (Figure S2). Of note, *TNNT2* was expressed in some of the cells in population 2, which is consistent with the strong, but not absolute correlation between %cTnT value and fraction of population 1 (Figure 1I), and previous studies showing that some EPDCs express *TNNT2* (Witty et al., 2014). These results show that the small-molecule differentiation protocol followed by lactate purification resulted in the absence of undifferentiated cells at day 25 and in the derivation of two distinct cell populations, one of which expresses high levels of CM markers, including *TNNT2*, *NKX2-5*, and *MEF2C* (population 1), and the other which expresses EPDC markers, including *SNAI2*, *DDR2*, *VIM*, and *ACTA2* (population 2). Of note, the protocols for generating iPSC-derived cardiomyocytes (iPSC-CMs) and iPSC-EPDCs both involve activating the WNT signaling pathway (Bao et al., 2016; Iyer et al., 2015) and have a shared intermediate mesoderm progenitor, but subsequent WNT inhibition directs differentiating cells to iPSC-CMs and endogenous levels of WNT signaling direct differentiating cells to iPSC-EPDCs (Witty et al., 2014) (Figure 1A). Therefore, our results suggest that iPSC-CVPC cellular heterogeneity results from suboptimal WNT inhibition in a subset of cells during differentiation, which then give rise to EPDCs.

iPSC-CVPCs Are Composed of Immature CMs and EPDCs

To estimate the relative abundances of CM and EPDC cells across our collection of iPSC-CVPC samples, we selected the top 50 significantly overexpressed genes in each of the three scRNA-seq populations (150 genes in total, $p < 10^{-13}$, edgeR, Table S2), obtained their expression levels in bulk RNA-seq from 180 iPSC-CVPCs, and inputted these values into CIBERSORT (Newman et al., 2015). We observed that the proportions of each cell type varied across the samples, although the iPSC-CVPCs tended to have a greater fraction of CMs (84.8% ± 31.8%, Figure 2A) than EPDCs (14.7% ± 32.0%), and essentially no stem cells (0% ± 0.8%). Due to lactate selection, the small number (67) of cells predicted to be ESCs may represent a distinct differentiated cell type that is more similar to stem cells than either CMs or EPDCs. The estimated fraction of CMs and EPDCs in the iPSC-CVPCs was highly correlated with %cTnT values ($r = 0.927$, $p \approx 0$, t test; Figure 2B), similar to that observed in the analysis of the scRNA-seq data (Figure 1J). Finally, we showed that the iPSC-CVPCs with high estimated CM or EPDC cellular fractions, respectively, showed higher expression of CM markers (*MEF2C*, *NKX2-5*, and *ACTN2*) and EPDC markers (*ACTA2*, *TAGLN*, *DDR2*, and *SNAI2*, Figure 2C). These results indicate that cellular heterogeneity across iPSC-CVPC samples largely reflects different proportions of CMs and EPDCs.

To characterize the similarities between the iPSC-CVPC transcriptomes and those of adult heart and artery samples, we performed a principal-component analysis (PCA) using the transcriptomes of 184 iPSCORE iPSCs, 180 iPSC-CVPCs, and the 1,072 GTEx samples, including left ventricle, atrial appendage, coronary artery, and aorta (GTEx Consortium et al., 2017). We found that principal component 1 (PC1) showed that iPSC-CVPCs correspond to an intermediate state between the iPSCs and adult samples, suggesting that the derived CMs and EPDCs are similar to immature cardiac cells (Figure 2D). PC2 divided

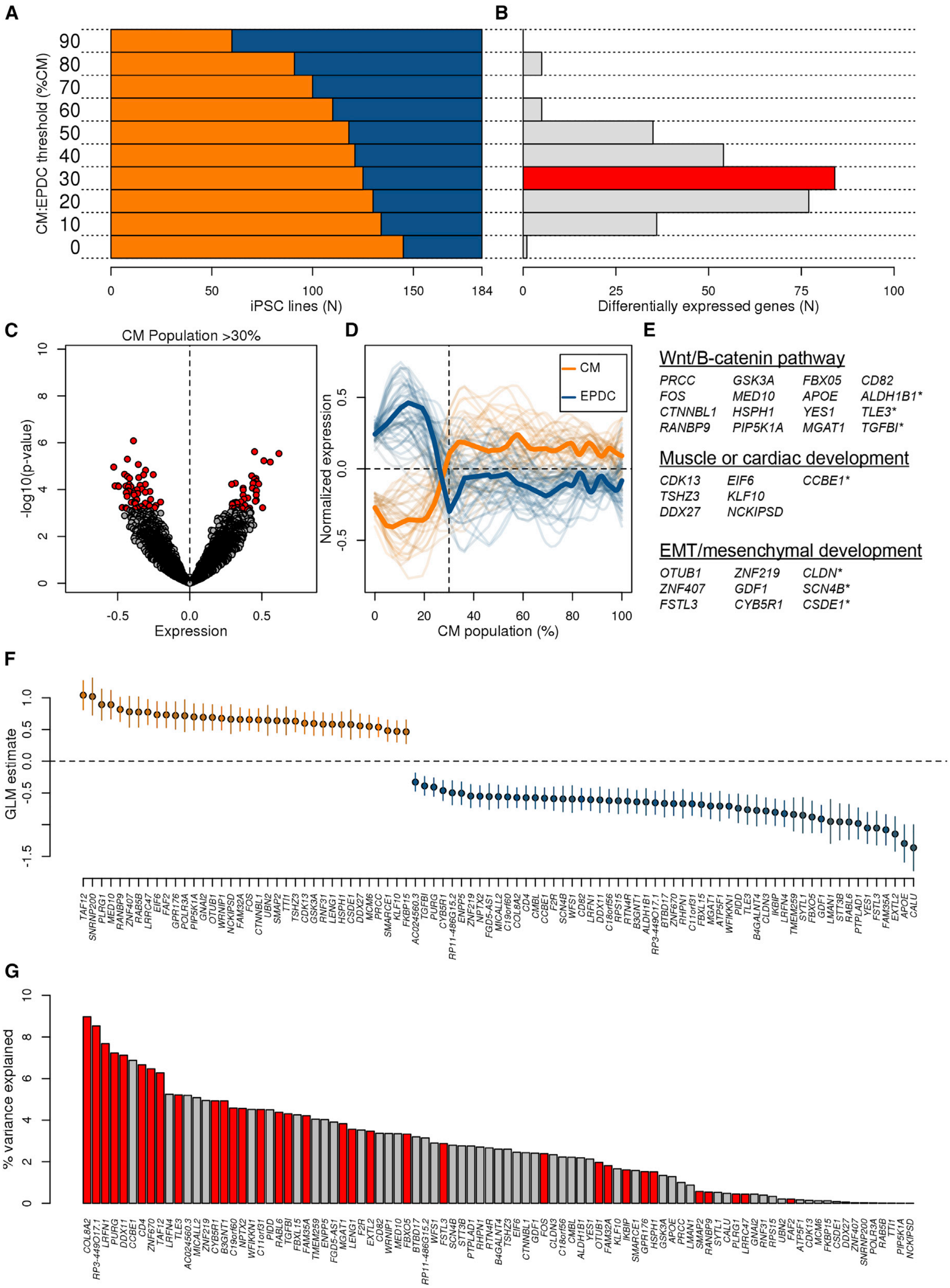

(legend on next page)

the samples based on cardiac lineage, namely the myocardium (left ventricles and atrial appendages) and epicardium (coronaries and aorta) (Perez-Pomares et al., 2016). This analysis shows that derived iPSC-CMs and iPSC-EPDCs lie on different cardiac developmental trajectories, with the CMs corresponding to immature myocardium and the EPDCs to immature epicardium.

iPSC Expression Signatures Affect Cardiac-Fate Differentiation

Although all iPSCORE iPSCs have previously been shown to be pluripotent (Panopoulos et al., 2017a), we sought to determine whether transcriptomic differences existed between the iPSC lines that derived CVPCs containing CMs versus those that gave rise to EPDCs (Figure 3A). Given that all 180 iPSC-CVPCs contain both CMs and EPDCs but at different ratios, we initially had to determine the optimal CM/EPDC ratio to group the iPSC lines into those that were CM-fated and those that were EPDC-fated. Thresholds for 193 iPSC-CVPCs that completed differentiation (harvested on day 25) were defined by the ratio of CM/EPDC estimates from CIBERSORT (estimated %CM/estimated %EPDC), while the 39 iPSC-CVPC differentiations terminated prior to day 25 for not forming a beating syncytium were assigned a CM/EPDC ratio of 0:100 (0% CM/100% EPDC). We tested ten different CM/EPDC ratios and found 116 autosomal genes that were differentially expressed at one or more of these ratios (Storey q value <0.1, t test; Figures 3A and 3B; Table S3A). We observed that the maximum number of the 116 genes (84, 72.5%) was differentially expressed at the 30:70 (CM/EPDC) threshold and 55 of them (47.4%) had their strongest p value at this ratio (Figure S3). For this reason, we determined that the 30:70 threshold was optimal, and grouped the iPSCs into 125 that were CM-fated (produced ≥30% CMs) and 59 that were EPDC-fated (produced >70% EPDCs; Figures 3B and S4; Table S3B).

Of the 84 autosomal differentially expressed genes at the 30:70 (CM/EPDC) threshold, 35 were overexpressed in the CM-fated iPSC lines and 49 were overexpressed in the EPDC-fated iPSCs (Figures 3B–3D). These genes have functions associated with three differentiation signatures: (1) Wnt/β-catenin pathway (13 genes); (2) muscle and/or cardiac differentiation (six genes); and (3) EMT and/or mesenchymal tissue development (six genes; Figure 3E and Table S3C). We noted that seven borderline significant autosomal genes were also involved in one of the three represented signatures, and therefore added them to the final list of differentially expressed genes. We investigated the associations between the expression levels of the final list of 91 signature genes in the 184 iPSCs and the fraction of CMs in the resulting iPSC-CVPCs using linear regression, and found significant associations for all genes (Figure 3F and Table S3D). These results show that, independently of the 30:70 (CM/EPDC) threshold used in the initial differential expression analysis, the expression levels of these signature genes in the 184 iPSCs were significantly associated with differentiation outcome (e.g., CM or EPDC fate).

Signature Genes Capture a Large Fraction of the Variance Underlying iPSC Fate Outcome

While the signature genes likely affected cardiac-fate determination, we did not expect each gene to contribute equally. To explore the impact of each gene individually on differentiation outcome, we calculated how much the 91 genes explained the variability underlying iPSC cell fate. To quantify the percent of variance explained by each gene (R^2), we fit a generalized linear regression model with a logit link function to each gene individually. We found that the percentage of variance explained by each

Figure 3. iPSC Gene Signatures Associated with Cardiac Differentiation Fate

(A) Testing of ten CM/EPDC ratios (0:100 to 90:10, with 10% increments) to determine the optimal threshold for defining an iPSC as CM-fated or EPDC-fated. For each threshold, the number of iPSC lines defined as CM-fated (orange) or EPDC-fated (blue) is shown.

(B) At the same thresholds indicated in (A), shown are the numbers of differentially expressed autosomal genes between the iPSC lines defined as CM-fated and EPDC-fated. The 30:70 threshold has the maximum number of differentially expressed genes.

(C) Volcano plot showing mean difference in expression levels for all autosomal genes between CM-fated iPSC lines and EPDC-fated iPSC lines (x axis) and p value (y axis, t test). A positive difference indicates overexpression in CM-fated iPSCs, whereas a negative difference indicates overexpression in EPDC-fated iPSCs. Significant genes are indicated in red.

(D) Expression levels of the 91 signature genes in iPSCs as a function of the %CM population in their corresponding iPSC-CVPC samples. Thick lines represent the average for 36 genes overexpressed in CM-fated iPSCs (orange) and for 55 genes overexpressed in EPDC-fated iPSCs (blue).

(E) WNT/β-catenin pathway, muscle/cardiac related, or EMT/mesenchymal development signature genes (those differentially expressed with nominal p values [$p < 0.0015$] indicated with an asterisk).

(F) GLM estimate (%CM population/expression) calculated for each signature gene. Mean and 95% confidence interval are shown.

(G) Bar plot showing the percentage of variability in iPSC fate that is explained by each of the 91 signature genes. Bars highlighted in red show the 35 signature genes identified by L1 normalization that independently contributed to variance. Due to the fact that the 91 genes do not have independent expression, the total sum of the percent variance explained is >1.

See also Figures S3–S5.

individual gene varied over three orders of magnitude ($1.73 \times 10^{-3} < R^2 < 8.97\%$; Figure 3G).

We next asked how these signature genes altogether captured variability in differentiation fate. As several of the signature genes had correlated expression levels (Figure S4), to reduce overfitting in the regression analysis we included an L1 norm penalty (i.e., LASSO regression) and used 10-fold cross-validation. We identified 35 genes that independently contributed to variance and whose expression levels collectively explained more than half of the variability in differentiation outcome across iPSC lines (average R^2 from the 10-fold cross-validation = 0.512). Together these data show that, while the proportion of variance explained by each of the signature genes varied widely, altogether they captured approximately half of the total variance underlying differential iPSC fate outcome.

Inherited Genetic Variation Does Not Influence Differentiation Outcome

We investigated whether genetic variation associated with the expression of any of the signature genes contributed to the differentiation outcome of iPSCs. We assessed the genotypes of 8,620,159 variants in each iPSC line and performed a genome-wide association study (GWAS) to investigate the association between genotype and the fraction of CMs in the corresponding iPSC-CVPCs. We found that none of these variants was associated with differentiation outcome at genome-wide significance ($p < 5 \times 10^{-8}$; Table S3E and Figure S5). To further examine the association between genetic background and differentiation outcome, we tested whether differentiations of different iPSC clones from the same individual, and from members of the same twin pair, were more likely to yield similar outcomes compared with differentiations of iPSC clones from individuals with different genetic backgrounds, and observed similar distributions (Figure S5). While our power to perform a GWAS was limited, this analysis shows that the genetic background did not contribute to the variance underlying iPSC differentiation outcome, indicating that non-genetic (i.e., clonality and passage) factors played a role in determining whether an iPSC line differentiated to CMs or EPDCs.

GSEA Implicates ELK1 Targets and Genes on the X Chromosome

To understand whether the transcriptomic differences between CM-fated and EPDC-fated iPSCs were associated with alterations in specific pathways or cellular function, we performed a gene-set enrichment analysis (GSEA) on 9,808 MSigDB gene sets (Subramanian et al., 2005) using the 15,228 expressed autosomal genes in the 184 iPSCs. We identified 22 gene sets that were significantly associated with iPSC fate, including enrichment in the 59 EPDC-fated iPSCs for extracellular matrix (Figure 4A and Table S4A) and in the 125 CM-fated iPSCs for transcription factor activity and ELK1 targets. To capture gene sets associated with expression differences on the X chromosome, we performed differential expression and GSEA on 113 female iPSC lines (87 CM-fated and 26 EPDC-fated). The two most significant gene sets were loci located within chrXp11 and chrXp22 (Figure 4B). Notably, the chrXp11 locus encodes both *ELK1* and *PORCN*, whose protein product (Porcupine) is targeted for WNT inhibition in CM differentiation protocols but not EPDC differentiation protocols (Mo et al., 2013; Wang et al., 2013) (Figure 4B). The chrXp22 locus includes the majority of genes (52/99, 52.5%) that are known to escape chromosome X inactivation (Tukiainen et al., 2017), and thus may potentially have varying X-linked gene dosage across female iPSCs. Overall, GSEA shows that genes differentially expressed between CM-fated and EPDC-fated iPSCs are involved in a variety of pathways, including ELK targets, and are potentially associated with the X chromosome activation status.

Sex Is Associated with iPSC Differentiation Outcome

To identify other iPSC factors potentially associated with differentiation outcome, we examined three characteristics of the 181 subjects in our study (sex, ethnicity, and age) and passage of the iPSCs at day 0. Analyzing the 125 CM-fated and 59 EPDC-fated iPSC lines with a general linear model, we found no association between differentiation outcome and age or ethnicity ($p > 0.8$; generalized linear model [GLM], *Z* test; Figure S6 and Table S4B), but observed a significant association with sex ($p = 2.57 \times 10^{-5}$, GLM, *Z* test; Figure 4C) and a trend for iPSC passage at day 0 ($p = 0.069$, GLM, *Z* test; Figure S6). These data suggest that iPSCs derived from female subjects and iPSCs with higher passages at day 0 had an increased predisposition for the CM fate. Furthermore, considering only the 191 completed differentiations (day-25 iPSC-CVPC samples), we found that iPSC-CVPC samples derived from female subjects compared with those derived from males had significantly higher %cTnT values (mean = 83.0% and 77.7%, respectively, for females and males; $p = 6.0 \times 10^{-4}$, Mann-Whitney U test; Figure S6) and a higher fraction of CMs ($p = 6.46 \times 10^{-4}$, Mann-Whitney U test). These results indicate that iPSCs derived from female subjects and, to a lesser extent, iPSCs that have spent more time in cell culture have a greater inherent predisposition to differentiate toward the CM lineage.

Female iPSCs with X Chromosome Reactivation Associated with CM Fate

Given the observation that female iPSCs have a greater potential to differentiate to CMs and that differential expression of chrXp11 genes were associated with differentiation

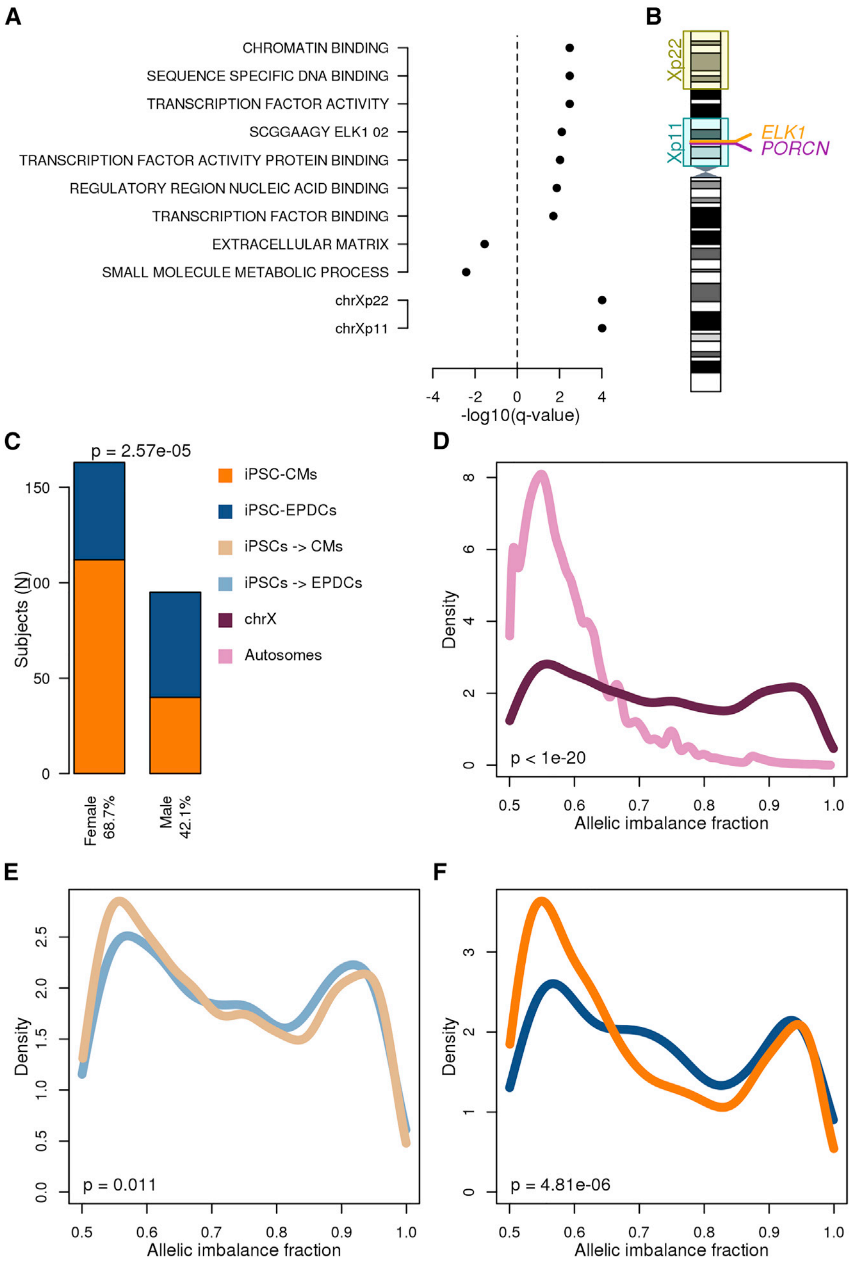

Figure 4. X Chromosome Gene Dosage Plays a Role in Cardiac Differentiation Fate

(A) GSEA results. For each gene set, $-\log_{10}$(q value) is shown. Positive values correspond to gene sets enriched in CM-fated iPSCs, whereas negative values correspond to EPDC-fated iPSCs. For autosomes all iPSCs were included (top), for the chromosome X only the 113 female iPSCs were analyzed (bottom). Storey q value was used to adjust for multiple testing hypothesis; q values <0.05 were considered significant.

(B) Cartoon showing the positions of differentially expressed loci on chromosome X and of *ELK1* and *PORCN*.

(C–F) Bar plot (C) showing the associations between sex and differentiation outcome (orange: iPSC-CVPC samples with CM fraction >30%; blue: with EPDC fraction >70%). p values were calculated using *Z* test. Density plots showing the differences in allelic imbalance fraction between: (D) autosomal genes (pink) and chrX genes outside of the pseudoautosomal region (maroon) in female iPSCs; (E) chrX genes in female CM-fated (light orange) and EPDC-fated (light blue) iPSCs; (F) chrX genes in female day 25 iPSC-CVPC samples with CM fraction >30% (orange) and EPDC fraction >70% (blue). p values in (D) to (F) were calculated using the Mann-Whitney U test.

See also Figure S6.

outcome, we asked whether variation in X chromosome inactivation (Xi) and activation (Xa) state across female iPSC lines was associated with CM or EPDC fate. Using RNA-seq data generated from the 113 female iPSCs, we evaluated allele-specific effects (ASE) of X chromosome and autosomal genes (Table S4C). We defined the strength of ASE for each gene as the fraction of RNA transcripts that were estimated to originate from the allele with higher expression (hereafter referred to as "allelic imbalance fraction" [AIF]). We observed that AIF in autosomal genes was close to 0.5, indicating that both alleles were equally expressed (Figure 4D), while AIF on the X chromosome in iPSCs tended to be bimodal, with some genes showing monoallelic expression (AIF ~1.0; XaXi) and others showing biallelic expression (AIF ~0.5; XaXa). We observed that AIF was less in the 87 CM-fated female iPSCs compared with the 26 EPDC-fated female iPSCs ($p = 0.011$, Mann-Whitney U test; Figure 4E) and that this difference in AIF became even more pronounced in the corresponding derived iPSC-CVPC samples ($p = 4.81 \times 10^{-6}$, Mann-Whitney U test; Figures 4F and S7). These findings show that in iPSCs, differential chromosome XaXi status as well as

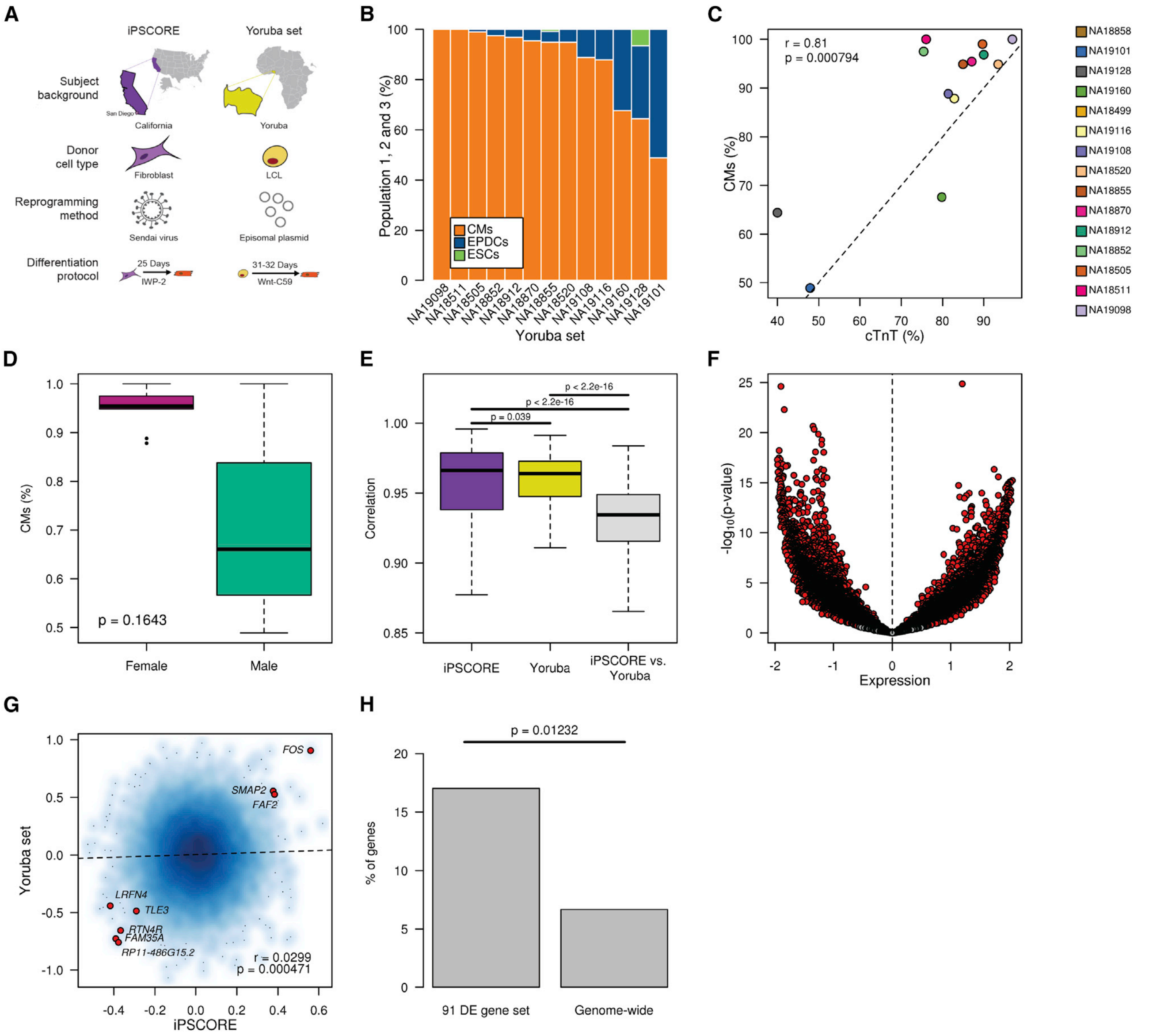

Figure 5. Validation of Association between iPSC Gene Signatures, Sex, and Differentiation Outcome

(A) Schematic depicting differences between the iPSCORE and Yoruba iPSC samples.

(B) Estimated fractions of CMs and EPDCs for 13 Yoruba iPSC-CM samples from RNA-seq using CIBERSORT (two iPSC-CMs did not have RNA-seq).

(C) Scatterplot showing the correlation between %cTnT and the fraction of cells in population 1 for 13 Yoruba iPSC-CM samples.

(D) Box plots showing the distribution of estimated fraction of cells in population 1 in females and males.

(E) Box plots showing correlation of gene expression in all 184 iPSCORE iPSCs with RNA-seq (purple), 34 Yoruba iPSCs with RNA-seq used for differentiation, and the pairwise comparison of the Yoruba iPSCs against the iPSCORE iPSCs (gray).

(F) Volcano plot showing mean difference in expression levels for all autosomal genes between 14 Yoruba iPSC lines that were successfully differentiated and 125 iPSCORE iPSC-CM-fated lines and p value (y axis, t test). Significant genes are indicated in red.

(G) Smooth color density scatterplot showing gene-expression differences between iPSCs with different fates in 184 iPSCORE iPSCs to the expression differences between iPSCs with different outcomes in Yoruba iPSCs (14 successful versus 20 terminated) (y axis). A positive difference indicates shared overexpression of genes between CM-fated iPSC in iPSCORE and successfully differentiated iPSC in the Yoruba set, whereas a negative difference indicates shared overexpression of genes between EPDC-fated iPSC in iPSCORE and terminated iPSC in

(legend continued on next page)

altered gene expression in chrXp22 and chrXp11 contributes to differences in cardiac-fate differentiation outcome.

Since we observed differences in X chromosome reactivation state between CM-fated and EPDC-fated female iPSCs, we next asked whether the two GSEA X chromosome-associated intervals (chrXp22 and chrXp11; Figure 4A) showed corresponding allelic imbalance trends. We plotted AIF differences, whereby a positive AIF difference indicates X chromosome reactivation in the 26 EPDC-fated iPSCs and a negative effect in the 87 CM-fated iPSCs (Figure S6). We observed that distinct regions across the X chromosome were differentially eroded in the EPDC-fated versus CM-fated iPSCs. In particular, chrXp22 showed X reactivation in CM-fated iPSCs ($p = 6.31 \times 10^{-3}$, Mann-Whitney U test), with both escape ($p = 0.020$, Mann-Whitney U test) and non-escape genes ($p = 0.023$, Mann-Whitney U test) showing evidence of reactivation (Figure S6). As chrXp22 contains more than half of the escape genes on the X chromosome, this observation confirms that increased X reactivation in CM-fated iPSCs results in increased expression of both escape and non-escape genes. As GSEA identified genes on chrXp11 to be overexpressed in CM-fated iPSCs, the lack of X reactivation in this interval ($p = 0.28$, Mann-Whitney U test) suggests that alternative regulatory mechanisms may also alter gene-expression levels on the X chromosome. Overall, these results suggest that differential X chromosome reactivation as well as other mechanisms underlying altered regulation of X chromosome and autosomal genes contribute to iPSC cardiac lineage fate determination.

Independent iPSC-CM Derivation Study Validates Findings

To assess the generalizability of our findings, we examined an independent collection of 39 iPSCs (Banovich et al., 2018) reprogrammed using an episomal plasmid from Yoruba lymphoblastoid cell lines (Figure 5A and Table S5). Differentiation of these lines resulted in the successful derivation of 13 iPSC-CMs (%cTnT range at day 32: 40–96.9), whereas 24 were terminated on or before day 10 due to the fact that they did not form a beating syncytium. To examine whether the successfully derived Yoruba iPSC-CMs showed the presence of EPDCs, we used RNA-seq data and CIBERSORT to estimate cellular compositions and observed variable relative distributions of CM and EPDC populations (Figure 5B). Consistent with our iPSCORE iPSC-CVPC samples, the estimated CM population fractions were significantly correlated with %cTnT values ($r = 0.81$, $p = 7.94 \times 10^{-4}$, t test; Figure 5C). To understand whether the CMs and EPDCs appear at the same time during differentiation, we analyzed data generated the from the Yoruba lines at four time points (Strober et al., 2019) and observed that both cardiac lineages are typically present by day 5 and that the ratio of these two cardiac cell types remains relatively stable past day 10 (Figure S7). Finally, Yoruba iPSC-CMs derived from females tended to have an increased percentage of CMs compared with those derived from males (Figure 5D). These observations show that the Yoruba iPSCs and derived cardiac cells could be used to investigate the generalizability of the associations that we had observed between transcriptomic differences in iPSCs and cardiac-fate differentiation outcome.

As several factors (Figure 5A) were different between the iPSCORE iPSC and Yoruba iPSC sets (i.e., different reprogramming method, genetic backgrounds, and donor cell types), we expected that there would be significant differences between their transcriptional profiles. We initially analyzed how correlated gene expression was: (1) within iPSCORE iPSCs; (2) within Yoruba iPSCs; and (3) between all pairwise comparisons of the iPSCs in these two different collections (Figure 5E). We observed high correlations of gene expression across iPSCs within each collection; however, the correlation between samples from different studies was significantly decreased, indicating that the two sets have significant genome-wide gene-expression differences. We next examined differential gene expression between the CM-fated iPSCORE iPSC and Yoruba iPSCs that successfully differentiated into iPSC-CMs (Figure 5F), and observed that the majority of genes (69.6% with q value <0.10) were significantly differentially expressed between the two iPSC sets. These results show that there are strong batch effects on gene expression between the iPSCORE and Yoruba iPSC lines.

We investigated whether, despite the strong batch effects on gene expression between iPSCORE and Yoruba iPSCs, we could detect inherent transcriptional differences affecting cardiac-fate determination that were shared between the iPSC sets. Given the relatively small size of the Yoruba study, there was insufficient power to detect transcriptional differences between the lines with different differentiation outcomes (successfully completed versus terminated). Therefore, for each gene, we compared the mean expression differences between iPSCs with different cardiac-fate outcomes in iPSCORE (CM fate minus EPDC

the Yoruba set. Of the 91 signature genes that were differentially expressed in the iPSCORE iPSCs based on cell fate, eight had nominally significant expression differences in the same direction in the Yoruba iPSC set (shown in red).
(H) Bar plot showing that the eight iPSCORE differentially expressed genes in (G) with nominal significant expression differences in the same direction (e.g., overexpressed or down regulated) in the Yoruba iPSCs are greater than random expectation.
See also Figure S7.

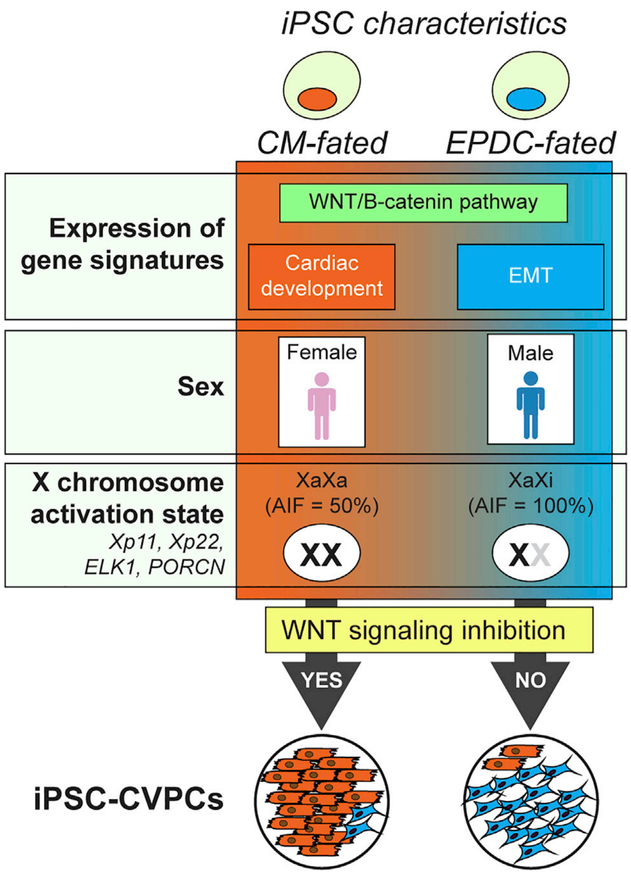

Figure 6. iPSC Characteristics that Influence Their Cardiac-Fate Determination

Cartoon showing iPSC characteristics that influence their cardiac-fate determination, including: (1) the expression levels of 91 genes grouped into three gene signature classes (WNT/B-catenin pathway, cardiac development genes, and genes involved in EMT); (2) sex: female iPSCs are more likely to differentiate to CMs than males; and (3) X chromosome activation state: female iPSCs that have activated both X chromosomes (XaXa) are more likely to differentiate to CMs.

fate) to the expression differences between iPSCs with different differentiation outcomes in the Yoruba set (successfully completed minus terminated; Figure 5G). We observed a small but significant correlation ($r = 0.0299$, $p = 4.71 \times 10^{-4}$, t test) between genes that were differentially expressed in the iPSCORE iPSCs and those that were differentially expressed in the Yoruba iPSCs. Furthermore, we specifically examined the 91 signature genes significantly associated with iPSCORE iPSC cardiac-fate outcome and found eight with nominally significant expression differences in the same direction (e.g., overexpressed or downregulated) in the two sets of iPSCs (Figure 5G), which is 2.5 times more than random expectation ($p = 0.012$, Fisher's exact test; Figure 5H). These data suggest that the iPSCORE iPSCs and Yoruba iPSCs shared transcriptional differences that affected cardiac-fate differentiation outcome.

DISCUSSION

While previous directed cardiac differentiation studies have observed the emergence of both cardiomyocytes and a non-contractile cell population, the origin of these non-contractile cells, and whether the same or different non-myocyte cell types are present across iPSC-CVPC samples, has not previously been addressed. We showed that two distinct cell types were present in 154 iPSC-CVPC samples derived from iPSCs in iPSCORE. One of the derived cell types were CMs, characterized by high expression levels of cardiac-specific genes, and the other derived cell type was EPDCs, characterized by high expression of marker genes for EMT, smooth muscle, and fibroblasts. We found the same two cardiac cell types present in iPSC-CMs derived from an independent collection of 39 Yoruba iPSCs, both of which were typically present by day 5, and their ratios remained relatively stable past day 10 (Banovich et al., 2018; Strober et al., 2019). A recent study showed that adding human ESC-derived epicardial cells to cardiomyocyte grafts *in vivo* improves transplantation efficacy, as it increases contractility, myofibril structure, and calcium handling and decreases tissue stiffness (Bargehr et al., 2019). Our findings suggest that the generation of EPDCs during iPSC-CM differentiation may enhance the structure of the derived CMs and that to efficiently use iPSC-CVPCs in a clinical setting, future studies may need to optimize the relative proportions of CMs and EPDCs that maximize their transplantation efficiency.

The scale of our study, 232 attempted differentiations of 191 iPSC lines into the cardiac lineage, provided the power to develop a framework to identify non-genetic transcriptional differences in iPSCs that influence their cardiac differentiation outcome. To minimize the factors that might influence differentiation outcome, such as the optimal cell confluence at which to start differentiation, we attempted to standardize all steps in the differentiation protocol in order to remove subjective decisions and diminish experimental differences between samples. We identified 91 signature genes whose differential expression was associated with differentiation outcome and showed that many of these genes are involved in cardiac development, including the Wnt/β-catenin pathway, muscle differentiation or cardiac-related functions, and the transition of epicardial cells to EPDCs by EMT (Figure 6). Many of the transcriptomic differences between iPSCORE iPSCs with CM fates versus those with EPDC fates may be due to aberrant epigenetic landscapes resulting from a combination of

the reprogramming method (Sendai virus) and cell of origin (fibroblasts). However, given that the Yoruba iPSCs were reprogrammed using a different method (episomal plasmid) and cell of origin (lymphoblastoid cell lines [LCLs]) and yet the iPSCORE and Yoruba iPSCs shared gene-expression differences associated with cardiac lineage outcome, it is likely that our findings will likely be generalizable to other collections of iPSCs. We hypothesize that the signature genes associated with cardiac lineage outcome will vary across iPSC collections and depend on the reprogramming method and cell type of origin, but will largely be involved in the same pathways identified in this study.

We observed that variability across iPSCs on X chromosome gene dosage (XaXa versus XaXi versus XY) played a role in cardiac lineage fate (Figure 6). While human iPSCs are known to have only partial XaXa (Barakat et al., 2015; Kim et al., 2014), we identified two loci (chrXp11 and chrXp22) encoding genes whose expression levels are associated with two distinct cardiac differentiation trajectories (CMs versus EPDCs). The higher expression of chrXp11 genes in CM-fated iPSCs may at least in part be due to fact that *ELK1* and *PORCN* are both encoded in this interval, as the protein product of *PORCN* (Porcupine) is inhibited by IWP-2 during CM differentiation (Mo et al., 2013) but not during EPDC differentiation (Bao et al., 2016; Iyer et al., 2015; Witty et al., 2014) (some EPDC protocols inhibit Porcupine but then reactivate the WNT pathway at a later time point [Guadix et al., 2017; Paik and Wu, 2017; Zhao et al., 2017]). Furthermore, we found that ELK1 targets are overexpressed in CM-fated iPSCs, which is consistent with previous studies showing that knockdown of *ELK1* in immortalized human bronchial epithelial cells, small airway epithelial cells, and luminal breast cancer cell line (MCF-7) is associated with increased EMT (Desai et al., 2017; Tatler et al., 2016). Also consistent with ELK1 playing a role in the association between X chromosome dosage and differentiation outcome is a previous study showing that *ELK1* overexpression or downregulation, respectively, mimics the phenotypes of XaXa or XaXi PSCs (Bruck et al., 2013). Of note, atrioventricular septal defects occur in ~20% of individuals with Down Syndrome (DS), and have a higher prevalence in female DS patients (Diogenes et al., 2017). Given that EPDCs play an essential role in septal formation (Gittenberger-de Groot et al., 2000), our study suggests that future work should investigate the extent to which X chromosome gene-expression levels are altered in cardiomyocytes from individuals with DS, and whether this is associated with the formation of fewer EPDCs.

Overall, our study suggests that expression differences of 91 signature and X chromosome genes result in the iPSCORE iPSC lines having differential propensities to respond to WNT inhibition during differentiation, and consequently are fated to produce iPSC-CVPC samples with different proportions of CMs and EPDCs. As iPSCs in the iPSCORE collection have passed standard quality checks to confirm their pluripotency and genomic integrity (Banovich et al., 2018; Panopoulos et al., 2017a), these transcriptomic expression differences associated with cardiac lineage outcome are not detected using current quality metrics. In conclusion, our findings suggest that to derive human iPSC lines that respond similarly in differentiation protocols, it may be necessary to improve reprogramming methods such that the transcriptome and X chromosome activation state is fully reset to the naive state, and incorporate inactivation of one of the X chromosomes in female lines as an early step in differentiation protocols.

EXPERIMENTAL PROCEDURES

Please refer to Supplemental Experimental Procedures for detailed methods.

Subject Information and Whole-Genome Sequencing

Individuals (108 females and 73 males) were recruited as part of the iPSCORE project (Panopoulos et al., 2017a) and included 7 MZ twin pairs, members of 32 families (2–10 members/family), and 71 singletons and were of diverse ancestries. Subject descriptions including subject sex, age, family, ethnicity, and cardiac diseases were collected during recruitment. As previously described (DeBoever et al., 2017), we generated whole-genome sequences from the blood or skin fibroblasts of the 181 subjects on the HiSeqX (Illumina; 150-bp paired end). The recruitment of these individuals was approved by the Institutional Review Boards of the University of California, San Diego and The Salk Institute (project no. 110776ZF).

iPSC Derivation and Somatic Mutation Analysis

As previously described, we reprogrammed fibroblast samples using non-integrative Cytotune Sendai virus (Life Technologies), and the 191 iPSCs (seven subjects had two or more clones each) were shown to be pluripotent and to have high genomic integrity with no or low numbers of somatic copy-number variants (CNVs) (D'Antonio et al., 2018; Panopoulos et al., 2017a).

Large-Scale Derivation of iPSC-CVPC Samples

To generate iPSC-derived cardiovascular progenitors (iPSC-CVPCs), we used a small-molecule cardiac differentiation protocol (Lian et al., 2013). The 25-day differentiation protocol consisted of five phases (Figure S1A); the optimizations for each step are described in the Supplemental Experimental Procedures.

Flow Cytometry

On day 25 of differentiation, iPSC-CVPCs were stained with cTnT antibody, acquired using fluorescence-activated cell sorting and analyzed using FlowJo V10.2.

Immunofluorescence Analysis of iPSC-CVPCs

Immunofluorescence was assessed in five iPSC-CVPC lines. Live frozen iPSC-CVPC harvested on day 25 were thawed, plated for 5 days, fixed, permeabilized, and incubated with antibodies (Table S1D).

Generation of RNA-Seq Data

For gene-expression profiling of iPSCs, we used RNA-seq data from 184 samples (cell lysates collected between passages 12 and 40). For gene-expression profiling of iPSC-CVPCs, we generated RNA-seq data from 180 samples at day 25 of differentiation. All RNA-seq samples were generated and analyzed using the same pipeline to obtain transcripts per million base pairs (TPM) (DeBoever et al., 2017).

Generation of scRNA-Seq Data

To capture the full spectrum of heterogeneity among the iPSC-CVPCs, we selected eight samples with variable percentage of cTnT (42.2%–95.8%). After removing proliferating cells and doublets, we obtained 34,905 cells.

CIBERSORT

Expression levels of the top 50 genes overexpressed in each of the three cell populations (total 150 genes) were used as input for CIBERSORT (Newman et al., 2015) to calculate the relative distribution of the three cell populations for the 180 iPSC-CVPC samples at day 25.

Characterizing Transcriptional Similarities of iPSCs, iPSC-CVPCs, and GTEx Adult Tissues

We performed PCA of RNA-seq on 184 iPSCs, 180 iPSC-CVPCs, and 1,072 RNA-seq samples from GTEx.

Determining Optimal CM/EPDC Ratio Estimates from CIBERSORT to Define iPSC Cardiac Fates

To obtain the optimal threshold, we conducted a series of differential expression analyses on 15,228 autosomal genes in the 184 iPSC lines (147 completed and 37 terminated) considering the ratio of population frequencies at ten thresholds. The 30:70 (CM/EPDC) ratio resulted in the highest number of differentially expressed genes (84 genes with Storey q value <0.1, t test), which is substantially greater than random expectation. Thus, we grouped the 184 iPSC lines into: (1) those that have CM fates, i.e., produced iPSC-CVPC with $\geq 30\%$ population 1; and (2) those that have EPDC fates, i.e., produced iPSC-CVPC with $>70\%$ population 2.

Comparing the Number of Differentially Expressed Genes with Random Expectation

To determine whether the number of significantly differentially expressed genes was higher than expected by chance, we shuffled the assignments of the 184 iPSC RNA-seq samples to differentiation fate (125 CM and 59 EPDC) 100 times.

Contribution of 91 Signature Genes in iPSCs to Determination of Cardiac Fate

For each of the 91 signature genes, we built a GLM with the expression of the gene as input and the differentiation outcome (e.g., percentage of population 1) as output using a logit link function. To understand the cumulative contribution of all 91 signature genes on cardiac differentiation fate, we built a GLM with an L1 norm penalty using the expression of all 91 genes as input and the differentiation outcome as output. To avoid overfitting the model, we used a 10-fold cross-validation.

Detecting Associations between Genetic Background and Differentiation Outcome

We obtained genotypes for 8,620,159 biallelic SNPs and short indels with allelic frequency $>5\%$ in the iPSCORE collection. Genotypes were obtained for each SNP in all individuals using *bcftools view* (Li, 2011). Linear regression was used to calculate the associations between the genotype of each variant and differentiation outcome (percent CM population in the iPSC-CVPCs), using passage at monolayer and sex as covariates.

GSEA Using the MSigDB Collection

We performed GSEA using the R *gage* package (Luo et al., 2009) on all MSigDB gene sets (Subramanian et al., 2005). False discovery rate correction was performed independently for each collection. The normalized mean expression difference between iPSCs that differentiated to CMs and iPSCs that differentiated to EPDCs was used as input for GSEA.

Associations between iPSC and Subject Features and Differentiation Outcome

A GLM was built in R using age, sex, ethnicity, age, and passage of the iPSCs at day 0 of differentiation as input and differentiation outcome as output (0 = EPDCs; 1 = CMs).

Identifying X Chromosome Inactivation in Female iPSCs and iPSC-CVPCs

To analyze X chromosome inactivation, we used 113 female iPSCs, of which 87 where CM-fated and 26 were EPDC-fated. We called ASE in RNA-seq from iPSC and iPSC-CVPCs as previously described (DeBoever et al., 2017). Genes lying in X chromosome pseudoautosomal (PAR) regions (PAR1: 60,001–2,699,520; PAR2: 154,931,044–155,260,560) were removed from analysis. We defined the strength of ASE for each gene as the fraction of RNA transcripts that were estimated to originate from the allele with higher expression (referred to as AIF).

Validation of Findings in Yoruba iPSC Set

The Yoruba iPSCs (Banovich et al., 2018) were generated from LCLs using episomal reprogramming. Differentiation was performed using a small-molecular method and iPSC-CMs were harvested on days 31 or 32. Fifteen lines successfully generated iPSC-CMs and 24 were terminated on or before day 10. We downloaded RNA-seq for 34 of the Yoruba iPSC and 13 iPSC-CM samples from the Gene Expression Omnibus (GEO: GSE89895) as well as 297 samples from 19 distinct iPSCs in a time-course experiment (days 0–15) performed on the same Yoruba iPSC samples (Strober et al., 2019). RNA-seq was aligned using STAR, and gene expression was quantified using the RSEM package and normalized to TPM. The RNA-seq for the 13 Yoruba iPSC-CMs and from all time-course

time points were analyzed using CIBERSORT, similar to the iPSCORE samples.

ACCESSION NUMBERS

Accession numbers for the RNA-seq data, scRNA-seq, and WGS genotypes are dbGaP: phs000924 (https://www.ncbi.nlm.nih.gov/projects/gap/cgi-bin/study.cgi?study_id=phs000924) and phs001325 (https://www.ncbi.nlm.nih.gov/projects/gap/cgi-bin/study.cgi?study_id=phs001325). The 191 iPSC lines are available through WiCell Research Institute: https://www.wicell.org/; NHLBI Next Gen Collection.

SUPPLEMENTAL INFORMATION

Supplemental Information can be found online at https://doi.org/10.1016/j.stemcr.2019.09.011.

AUTHOR CONTRIBUTIONS

K.A.F., A.D.-C., M.K.R.D., and M.D. conceived the study. A.D.-C. and K.F. performed iPSC-CVPC differentiations. A.D.-C. and F.C. generated molecular data. S.H. generated immunofluorescence images. M.C.W. generated Yoruba iPSC-CMs. F.S. and A.D.-C. generated scRNA-seq data. M.K.R.D., W.W.G., H.M., E.N.S., J.P.N., and M.D. performed data processing and computational analyses. M.P., E.A., and K.A.F. oversaw the study. M.K.R.D., M.D., and K.A.F. prepared the manuscript.

ACKNOWLEDGMENTS

This work was supported by a CIRM grant GC1R-06673-B, NSF-CMMI division award 1728497, and NIH grants HG008118, HL107442, DK105541, and DK112155. M.K.R.D. and J.P.N. were supported by T15LM011271. W.W.G. was supported by F31HL142151.

Received: April 12, 2019
Revised: September 27, 2019
Accepted: September 30, 2019
Published: October 24, 2019

REFERENCES

Banovich, N.E., Li, Y.I., Raj, A., Ward, M.C., Greenside, P., Calderon, D., Tung, P.Y., Burnett, J.E., Myrthil, M., Thomas, S.M., et al. (2018). Impact of regulatory variation across human iPSCs and differentiated cells. Genome Res. *28*, 122–131.

Bao, X., Lian, X., Hacker, T.A., Schmuck, E.G., Qian, T., Bhute, V.J., Han, T., Shi, M., Drowley, L., Plowright, A., et al. (2016). Long-term self-renewing human epicardial cells generated from pluripotent stem cells under defined xeno-free conditions. Nat. Biomed. Eng. *1*. https://doi.org/10.1038/s41551-016-0003.

Barakat, T.S., Ghazvini, M., de Hoon, B., Li, T., Eussen, B., Douben, H., van der Linden, R., van der Stap, N., Boter, M., Laven, J.S., et al. (2015). Stable X chromosome reactivation in female human induced pluripotent stem cells. Stem Cell Reports *4*, 199–208.

Bargehr, J., Ong, L.P., Colzani, M., Davaapil, H., Hofsteen, P., Bhandari, S., Gambardella, L., Le Novere, N., Iyer, D., Sampaziotis, F., et al. (2019). Epicardial cells derived from human embryonic stem cells augment cardiomyocyte-driven heart regeneration. Nat. Biotechnol. *37*, 895–906.

Bruck, T., Yanuka, O., and Benvenisty, N. (2013). Human pluripotent stem cells with distinct X inactivation status show molecular and cellular differences controlled by the X-linked ELK-1 gene. Cell Rep. *4*, 262–270.

Burridge, P.W., Matsa, E., Shukla, P., Lin, Z.C., Churko, J.M., Ebert, A.D., Lan, F., Diecke, S., Huber, B., Mordwinkin, N.M., et al. (2014). Chemically defined generation of human cardiomyocytes. Nat. Methods *11*, 855–860.

D'Antonio, M., Benaglio, P., Jakubosky, D., Greenwald, W.W., Matsui, H., Donovan, M.K.R., Li, H., Smith, E.N., D'Antonio-Chronowska, A., and Frazer, K.A. (2018). Insights into the mutational burden of human induced pluripotent stem cells from an integrative multi-omics approach. Cell Rep. *24*, 883–894.

DeBoever, C., Li, H., Jakubosky, D., Benaglio, P., Reyna, J., Olson, K.M., Huang, H., Biggs, W., Sandoval, E., D'Antonio, M., et al. (2017). Large-scale profiling reveals the influence of genetic variation on gene expression in human induced pluripotent stem cells. Cell Stem Cell *20*, 533–546.e7.

Desai, K., Aiyappa, R., Prabhu, J.S., Nair, M.G., Lawrence, P.V., Korlimarla, A., Ce, A., Alexander, A., Kaluve, R.S., Manjunath, S., et al. (2017). $HR^{+}HER2^{-}$ breast cancers with growth factor receptor-mediated EMT have a poor prognosis and lapatinib downregulates EMT in MCF-7 cells. Tumour Biol. *39*. https://doi.org/10.1177/1010428317695028.

Diogenes, T.C.P., Mourato, F.A., de Lima Filho, J.L., and Mattos, S.D.S. (2017). Gender differences in the prevalence of congenital heart disease in Down's syndrome: a brief meta-analysis. BMC Med. Genet. *18*, 111.

Dubois, N.C., Craft, A.M., Sharma, P., Elliott, D.A., Stanley, E.G., Elefanty, A.G., Gramolini, A., and Keller, G. (2011). SIRPA is a specific cell-surface marker for isolating cardiomyocytes derived from human pluripotent stem cells. Nat. Biotechnol. *29*, 1011–1018.

Gittenberger-de Groot, A.C., Vrancken Peeters, M.P., Bergwerff, M., Mentink, M.M., and Poelmann, R.E. (2000). Epicardial outgrowth inhibition leads to compensatory mesothelial outflow tract collar and abnormal cardiac septation and coronary formation. Circ. Res. *87*, 969–971.

GTEx Consortium, Laboratory, Data Analysis &Coordinating Center (LDACC)—Analysis Working Group, Statistical Methods groups—Analysis Working Group, Enhancing GTEx (eGTEx) groups, NIH Common Fund, NIH/NCI, NIH/NHGRI, NIH/NIMH, NIH/NIDA, Biospecimen Collection Source Site—NDRI, et al. (2017). Genetic effects on gene expression across human tissues. Nature *550*, 204–213.

Guadix, J.A., Orlova, V.V., Giacomelli, E., Bellin, M., Ribeiro, M.C., Mummery, C.L., Perez-Pomares, J.M., and Passier, R. (2017). Human pluripotent stem cell differentiation into functional epicardial progenitor cells. Stem Cell Reports *9*, 1754–1764.

Iyer, D., Gambardella, L., Bernard, W.G., Serrano, F., Mascetti, V.L., Pedersen, R.A., Talasila, A., and Sinha, S. (2015). Robust derivation of epicardium and its differentiated smooth muscle cell progeny

from human pluripotent stem cells. Development *142*, 1528–1541.

Kilpinen, H., Goncalves, A., Leha, A., Afzal, V., Alasoo, K., Ashford, S., Bala, S., Bensaddek, D., Casale, F.P., Culley, O.J., et al. (2017). Common genetic variation drives molecular heterogeneity in human iPSCs. Nature *546*, 370–375.

Kim, K.Y., Hysolli, E., Tanaka, Y., Wang, B., Jung, Y.W., Pan, X., Weissman, S.M., and Park, I.H. (2014). X chromosome of female cells shows dynamic changes in status during human somatic cell reprogramming. Stem Cell Reports *2*, 896–909.

Li, H. (2011). A statistical framework for SNP calling, mutation discovery, association mapping and population genetical parameter estimation from sequencing data. Bioinformatics *27*, 2987–2993.

Lian, X., Zhang, J., Azarin, S.M., Zhu, K., Hazeltine, L.B., Bao, X., Hsiao, C., Kamp, T.J., and Palecek, S.P. (2013). Directed cardiomyocyte differentiation from human pluripotent stem cells by modulating Wnt/beta-catenin signaling under fully defined conditions. Nat. Protoc. *8*, 162–175.

Lian, X., Bao, X., Zilberter, M., Westman, M., Fisahn, A., Hsiao, C., Hazeltine, L.B., Dunn, K.K., Kamp, T.J., and Palecek, S.P. (2015). Chemically defined, albumin-free human cardiomyocyte generation. Nat. Methods *12*, 595–596.

Luo, W., Friedman, M.S., Shedden, K., Hankenson, K.D., and Woolf, P.J. (2009). GAGE: generally applicable gene set enrichment for pathway analysis. BMC Bioinformatics *10*, 161.

Mo, M.L., Li, M.R., Chen, Z., Liu, X.W., Sheng, Q., and Zhou, H.M. (2013). Inhibition of the Wnt palmitoyltransferase porcupine suppresses cell growth and downregulates the Wnt/beta-catenin pathway in gastric cancer. Oncol. Lett. *5*, 1719–1723.

Newman, A.M., Liu, C.L., Green, M.R., Gentles, A.J., Feng, W., Xu, Y., Hoang, C.D., Diehn, M., and Alizadeh, A.A. (2015). Robust enumeration of cell subsets from tissue expression profiles. Nat. Methods *12*, 453–457.

Paik, D.T., and Wu, J.C. (2017). Simply derived epicardial cells. Nat. Biomed. Eng. *1*. https://doi.org/10.1038/s41551-016-0015.

Panopoulos, A.D., D'Antonio, M., Benaglio, P., Williams, R., Hashem, S.I., Schuldt, B.M., DeBoever, C., Arias, A.D., Garcia, M., Nelson, B.C., et al. (2017a). iPSCORE: a resource of 222 iPSC lines enabling functional characterization of genetic variation across a variety of cell types. Stem Cell Reports *8*, 1086–1100.

Panopoulos, A.D., Smith, E.N., Arias, A.D., Shepard, P.J., Hishida, Y., Modesto, V., Diffenderfer, K.E., Conner, C., Biggs, W., Sandoval, E., et al. (2017b). Aberrant DNA methylation in human iPSCs associates with MYC-binding motifs in a clone-specific manner independent of genetics. Cell Stem Cell *20*, 505–517.e6.

Perez-Pomares, J.M., de la Pompa, J.L., Franco, D., Henderson, D., Ho, S.Y., Houyel, L., Kelly, R.G., Sedmera, D., Sheppard, M., Sperling, S., et al. (2016). Congenital coronary artery anomalies: a bridge from embryology to anatomy and pathophysiology—a position statement of the Development, Anatomy, and Pathology ESC Working Group. Cardiovasc. Res. *109*, 204–216.

Strober, B.J., Elorbany, R., Rhodes, K., Krishnan, N., Tayeb, K., Battle, A., and Gilad, Y. (2019). Dynamic genetic regulation of gene expression during cellular differentiation. Science *364*, 1287–1290.

Subramanian, A., Tamayo, P., Mootha, V.K., Mukherjee, S., Ebert, B.L., Gillette, M.A., Paulovich, A., Pomeroy, S.L., Golub, T.R., Lander, E.S., et al. (2005). Gene set enrichment analysis: a knowledge-based approach for interpreting genome-wide expression profiles. Proc. Natl. Acad. Sci. U S A *102*, 15545–15550.

Tatler, A.L., Habgood, A., Porte, J., John, A.E., Stavrou, A., Hodge, E., Kerama-Likoko, C., Violette, S.M., Weinreb, P.H., Knox, A.J., et al. (2016). Reduced Ets domain-containing protein Elk1 promotes pulmonary fibrosis via increased integrin alphavbeta6 expression. J. Biol. Chem. *291*, 9540–9553.

Tohyama, S., Hattori, F., Sano, M., Hishiki, T., Nagahata, Y., Matsuura, T., Hashimoto, H., Suzuki, T., Yamashita, H., Satoh, Y., et al. (2013). Distinct metabolic flow enables large-scale purification of mouse and human pluripotent stem cell-derived cardiomyocytes. Cell Stem Cell *12*, 127–137.

Tukiainen, T., Villani, A.C., Yen, A., Rivas, M.A., Marshall, J.L., Satija, R., Aguirre, M., Gauthier, L., Fleharty, M., Kirby, A., et al. (2017). Landscape of X chromosome inactivation across human tissues. Nature *550*, 244–248.

Wang, X., Moon, J., Dodge, M.E., Pan, X., Zhang, L., Hanson, J.M., Tuladhar, R., Ma, Z., Shi, H., Williams, N.S., et al. (2013). The development of highly potent inhibitors for porcupine. J. Med. Chem. *56*, 2700–2704.

Witty, A.D., Mihic, A., Tam, R.Y., Fisher, S.A., Mikryukov, A., Shoichet, M.S., Li, R.K., Kattman, S.J., and Keller, G. (2014). Generation of the epicardial lineage from human pluripotent stem cells. Nat. Biotechnol. *32*, 1026–1035.

Zhao, J., Cao, H., Tian, L., Huo, W., Zhai, K., Wang, P., Ji, G., and Ma, Y. (2017). Efficient differentiation of TBX18(+)/WT1(+) epicardial-like cells from human pluripotent stem cells using small molecular compounds. Stem Cells Dev. *26*, 528–540.

Stem Cell Reports, Volume *13*

Supplemental Information

Association of Human iPSC Gene Signatures and X Chromosome Dosage with Two Distinct Cardiac Differentiation Trajectories

Agnieszka D'Antonio-Chronowska, Margaret K.R. Donovan, William W. Young Greenwald, Jennifer Phuong Nguyen, Kyohei Fujita, Sherin Hashem, Hiroko Matsui, Francesca Soncin, Mana Parast, Michelle C. Ward, Florence Coulet, Erin N. Smith, Eric Adler, Matteo D'Antonio, and Kelly A. Frazer

SUPPLEMENTAL MATERIAL

TABLE OF CONTENTS

SUPPLEMENTAL FIGURES 2-14

Figure S1. Optimization of iPSC-CVPC differentiation protocol 2-4

Figure S2: Distribution of single cells across the three clusters 5-6

Figure S3. Differentially expressed genes at ten CM:EPDC thresholds 7-8

Figure S4. Expression of 91 signature genes in 184 iPSCs 9-10

Figure S5. Associations between genetic background and differentiation outcome 11

Figure S6. X chromosome inactivation in iPSCs 12-13

Figure S7. Cell populations at 15 time points during iPSC-CM differentiations 14

TABLE LEGENDS 15-20

SUPPLEMENTAL EXPERIMENTAL PROCEDURES 20-29

REFERENCES 30-31

Figure S1: Optimization of iPSC-CVPC differentiation protocol. Related to Figure 1.

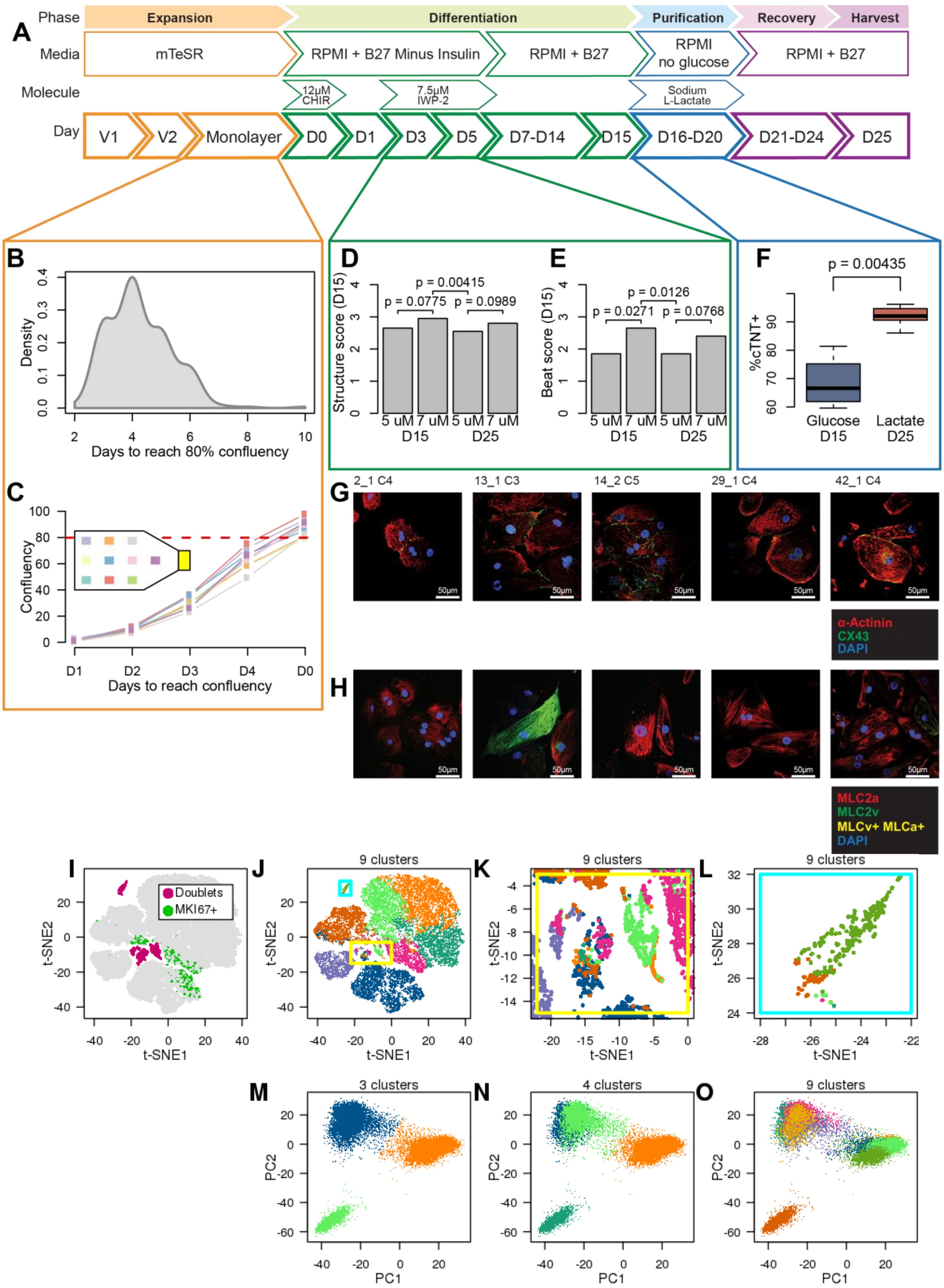

(A) Schematic of differentiation protocol. To achieve large-scale derivation of iPSC-derived cardiovascular progenitor cells (iPSC-CVPCs), we optimized existing small molecule protocols to increase throughput and efficiency. We optimized several steps of the protocol, including automating detection of iPSC monolayer confluency (orange box), optimizing the IWP-2 concentration (green box), and incorporating lactate selection (blue box).

(B) Density plot showing distribution of days recorded from 253 iPSC samples to reach 80% confluency. It was observed that 75-85% iPSC monolayer confluence at day 0 (D0), which marks the initiation of differentiation by WNT activation, yields the most efficient differentiations (Burridge et al., 2014; Lian et al., 2013); however, iPSCs have variable growth rates, and therefore it would be difficult to consistently achieve this confluency across hundreds of lines.

(C) Confluency levels from one line (2_3) measured from ten sections of T150 flask. Due to observed variability in iPSC growth rates, we developed ccEstimate, an automated tool that determines confluency by processing images from multiple locations in three T150 flasks over a period of at least 72 hours, and then estimates when a particular iPSC line will reach an average confluency of 80% based on its growth rate (Figure S2). Circles represent measured values. The points at D0 were obtained based on the ccEstimate algorithm's predictions.

(D, E) Effects of IWP-2 concentration (5.0µM or 7.5µM) given on D3 or D3 and D4 on (D) structure score and (E) beat score (Table S1H). We optimized WNT inhibition at D3, which is required for robust iPSC-CVPC differentiations by testing two concentrations of IWP-2 (5µM and 7.5µM) both with and without a media change between D3 and D4. We differentiated one iPSC line (iPSCORE_2_3_iPSC_C5_P13) under each of the four IWP-2 conditions, and observed that 7.5 µM IWP-2 without a media change between D3 and D4 resulted in iPSC-CVPCs with the thickest structures and strongest beating (structure score: $p = 0.00415$, beat score: $p = 0.0126$; Paired t test) (Table S1H). P-values were calculated using paired t test.

(F) Effects of metabolic purification of iPSC-CVPCs by lactate and glucose. To examine the efficacy of using lactate for iPSC-CVPC metabolic purification (Burridge et al., 2014; Kadari et al., 2015; Tohyama et al., 2013), we tested lactate and glucose at D16 in three different iPSC-CVPC lines (2_3, 8_2, and 3_2), and found that lactate resulted in significantly purer iPSC-CVPC populations at D25 (93.95% vs. 68.55%; $p = 0.00435$). P-values were calculated using Mann-Whitney U test.

(G) Immunofluorescence staining of five iPSC-CVPC lines at D30 with IF markers DAPI (blue), ACTN1 (red), and CX43 (green).

(H) Immunofluorescence staining of five iPSC-CVPC lines at D30 with IF markers DAPI (blue), MLC2a+ (red), and MLC2v+ (green), and MLC2v+ MLC2a+ (yellow).

(I) Filtering doublets from and selection of k-means clustering k in scRNA-seq: t-SNE plot of gene expression from 36,839 cells from 8 iPSC-CVPC and 1 ESC. We removed 1,934 cells from the scRNA-seq analysis (Figure 1), including cells that were visually identified as being doublets (pink) and actively dividing cells with (>2 UMI MK167, green).

(J) Cells are colored by k-means clusters with k = 9 cluster assignment. Cells that clustered together in the t-SNE plot, but were assigned to multiple different clusters were considered as doublets. Doublets are highlighted in the yellow and cyan box.

(K) Zoomed in region of the yellow box.

(L) Zoomed in region of the cyan box.

(M-O) PCA of gene expression from 36,839 cells from 8 iPSC-CVPC and 1 ESC colored by k-means clustering: (M) k = 3, (N) k=4; and (O) k = 9. The PCA shows that three cell populations are present and thus we used three clusters (k = 3) for all scRNA-seq analysis. In summary, we analyzed 34,905 single cells assigned to three cell populations.

Figure S2: Distribution of single cells across the three clusters. Related to Figure 1.

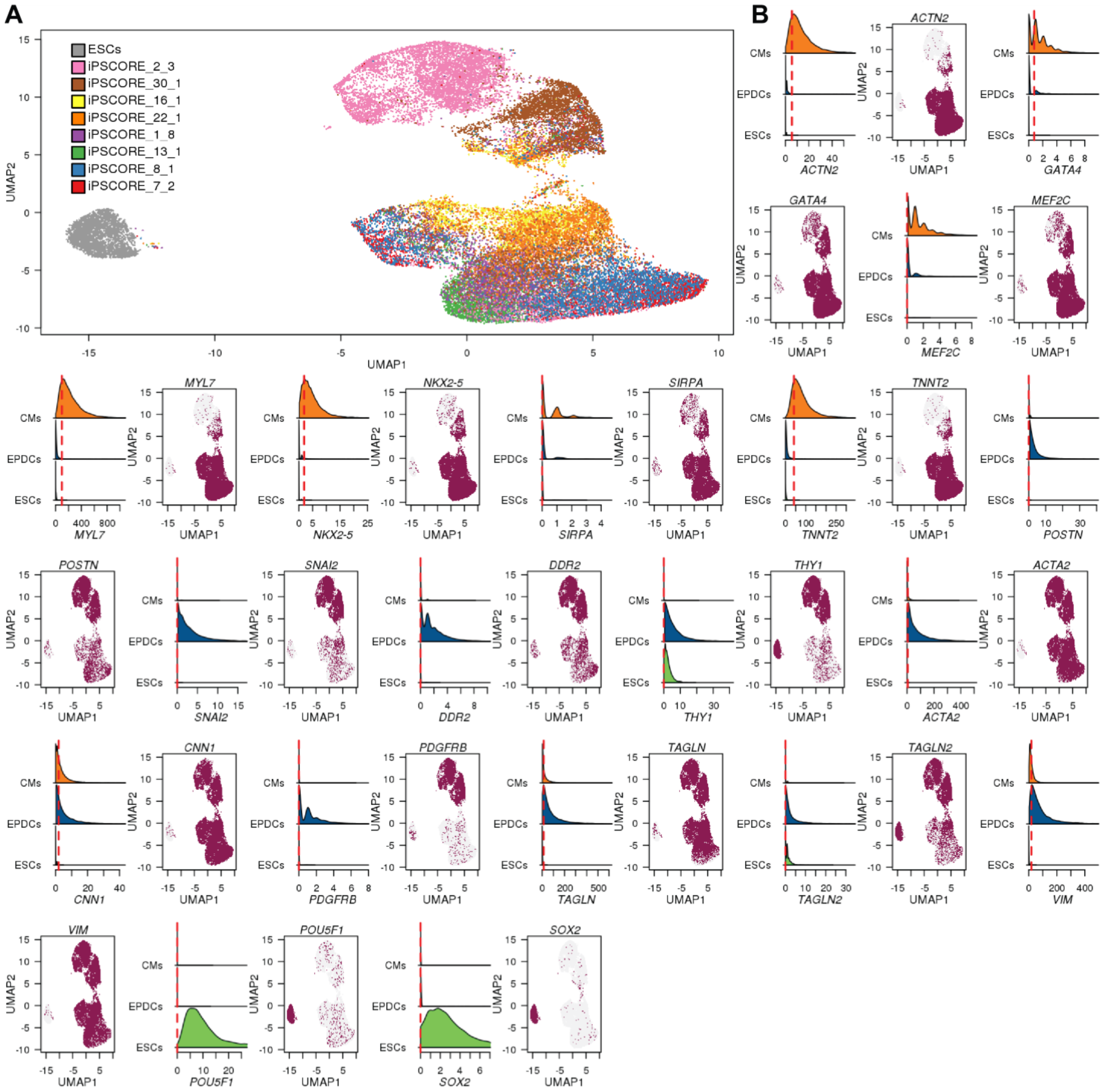

(A) Distribution of single cells across the three cell populations for the nine analyzed samples: scRNA-seq UMAP plots from 34,905 single cells showing their distributions across the three different clusters for the nine analyzed samples (8 iPSC-CVPCs lines and one ESC line). Each of the nine samples have a different color.

(B) Expression levels for marker genes: For each gene in Figure 1J, density plots show the gene expression distribution across all cells associated with each cell population (Population 1 = orange; Population 2 = blue;

Population 3 = green.). Red dashed line represents the median. UMAP plots from 34,905 cells show in maroon all the cells expressing the indicated marker gene higher than its median expression across the three populations.

Figure S3: Differentially expressed genes at ten CM:EPDC thresholds. Related to Figure 3.

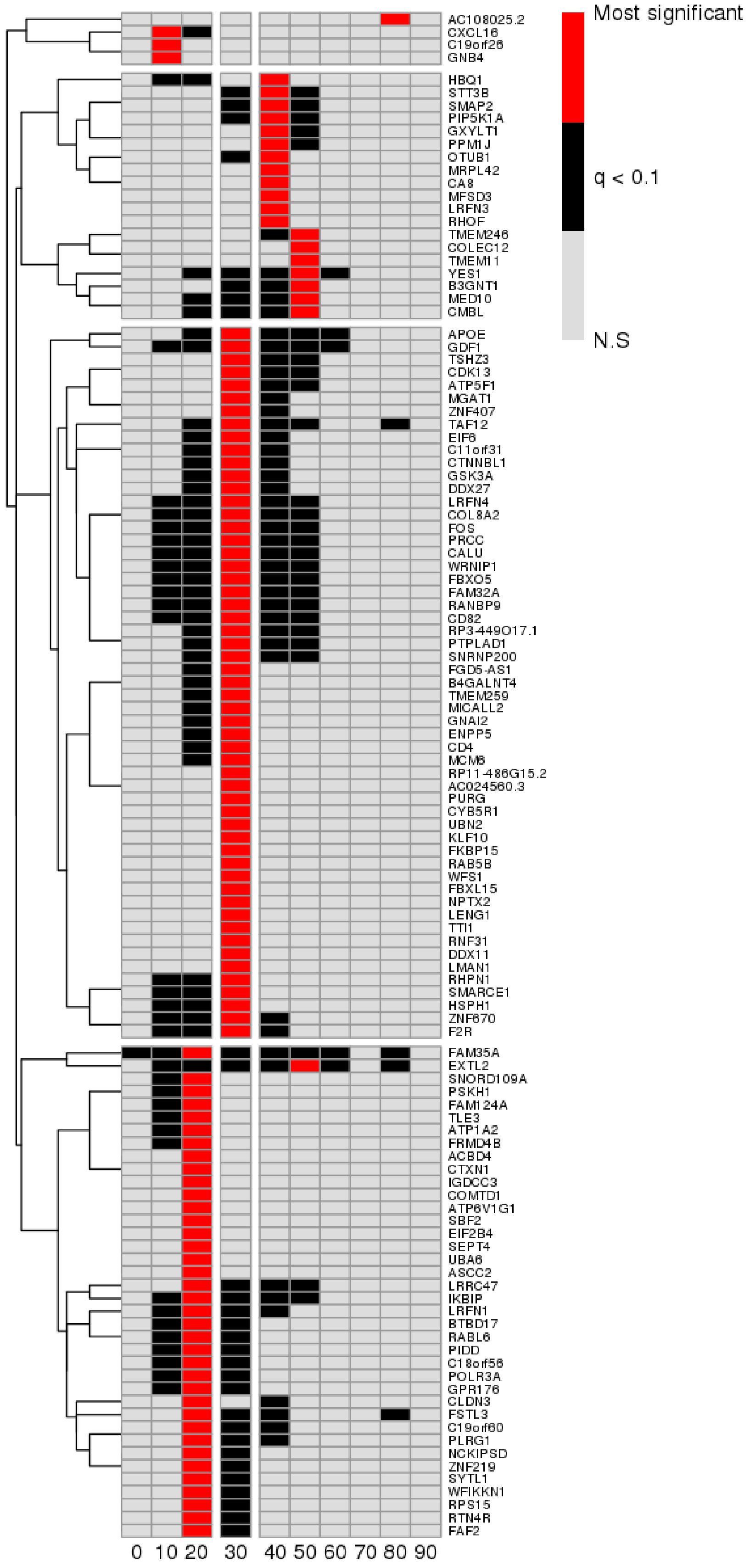

Analysis to determine the optimal CM:EPDC ratio to group the iPSC lines into those that were CM-fated and those that were EPDC-fated. We tested ten different CM:EPDC ratios and found 116 autosomal genes that were differentially expressed at one or more of these ratios (Storey q-value < 0.1, t-test Figure 3A,B, Table S3A-B). Heatmap showing at each threshold whether each of the 116 differentially expressed genes is significant (red or black = q-value < 0.1; gray: q-value > 0.1). Red squares indicate the threshold at which each gene is most significant. The heatmap shows that the 30:70 (CM:EPDC) threshold is where the most genes have their highest significance. For the vast majority of genes, the threshold at which they are most significantly differentially expressed is between 20% and 40%, confirming that the 30% threshold is optimal to distinguish between CM-fated and EPDC-fated iPSCs.

Figure S4: Expression of 91 signature genes in 184 iPSCs. Related to Figure 3.

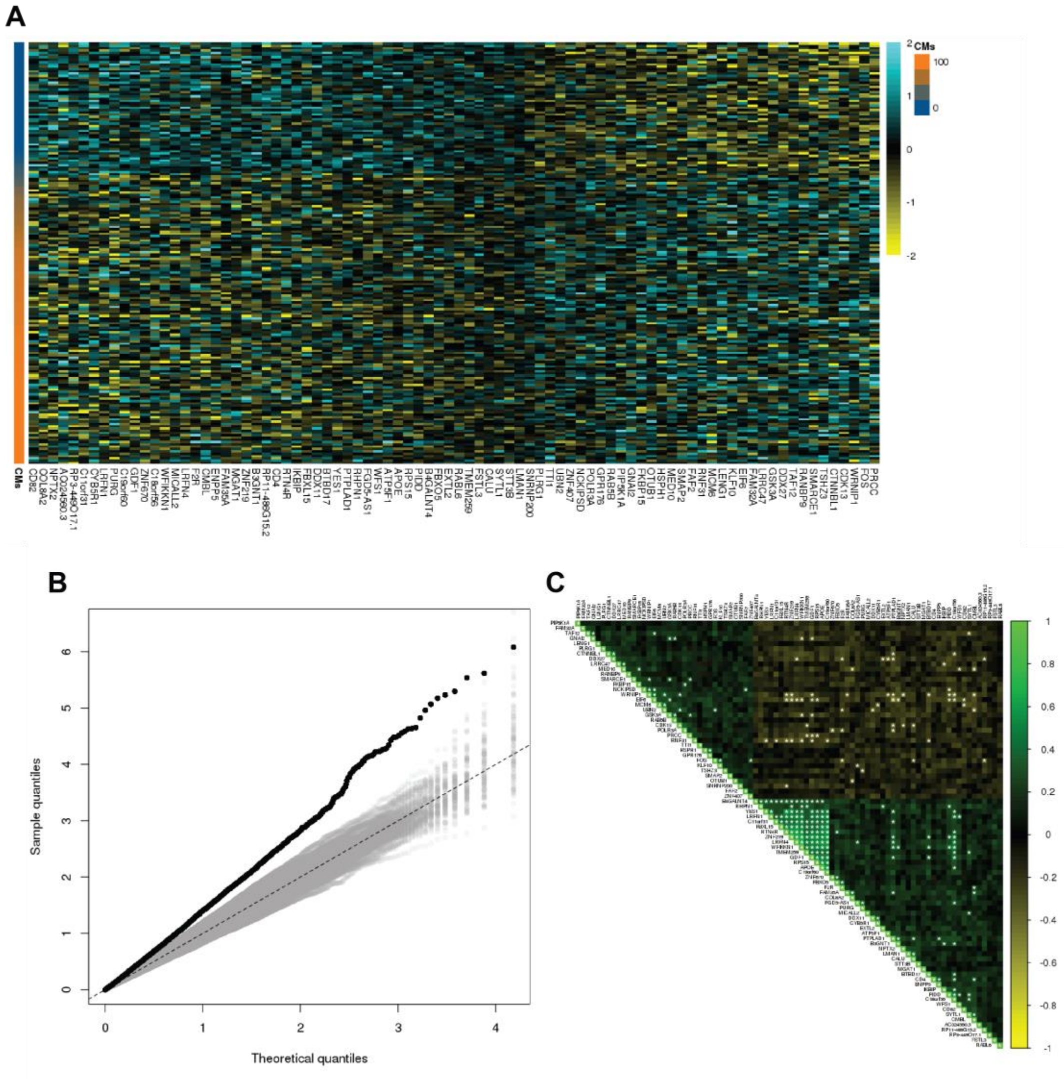

(A) Heatmap showing the expression levels of the 91 signature genes differentially expressed between CM-fated and EPDC-fated iPSCs. Each row represents an iPSC sample. The “CMs” scale represents the %CM population (Population 1) in the associated iPSC-CVPC samples for each iPSC.

(B) Comparison between the observed number of signature genes and random expectation: A QQ plot showing that the observed p-value distribution (black) was substantially different than random expectation. To determine if the identification of 84 signature genes that were significantly differentially expressed between CM-fated and EPDC-fated iPSCs was higher than random expectation, we shuffled the assignments of the 184 iPSC RNA-seq samples to differentiation fates (125 CM and 59 EPDC) 100 times. For each shuffle, we performed differential expression analysis and obtained the number of genes that were significantly differentially expressed (gray).

(C) Correlation between the 91 signature genes differentially expressed between CM-fated and EPDC-fated iPSCs: Heatmap showing the correlation of expression levels in the 125 CM-fated iPSCs versus the 59 EPDC-fated iPSCs for the 91 signature genes. White stars show significant correlations (Bonferroni p-value < 0.05).

Figure S5: Associations between genetic background and differentiation outcome

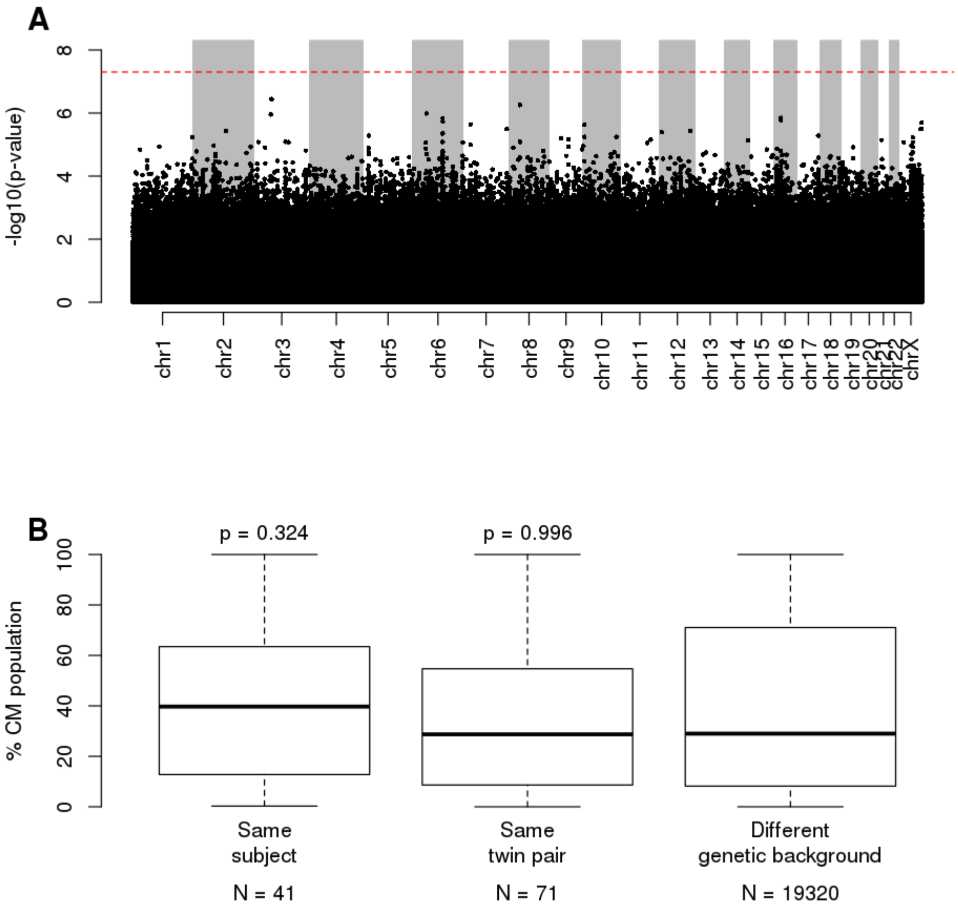

(A) Manhattan plot showing the association between genetic variation and differentiation outcome (measured as % CM population in iPSC-CVPCs). Red dashed line shows p-value = 0.05 adjusted using Bonferroni's method ($p = 5 \times 10^{-8}$).

(B) Boxplots showing distributions of the differences in the %CM population between differentiations of different iPSC clones from the same subject, from the same twin pair, and from individuals with different genetic backgrounds. P-values were calculated using Mann-Whitney U test.

Figure S6: X chromosome inactivation in iPSCs. Related to Figure 4.

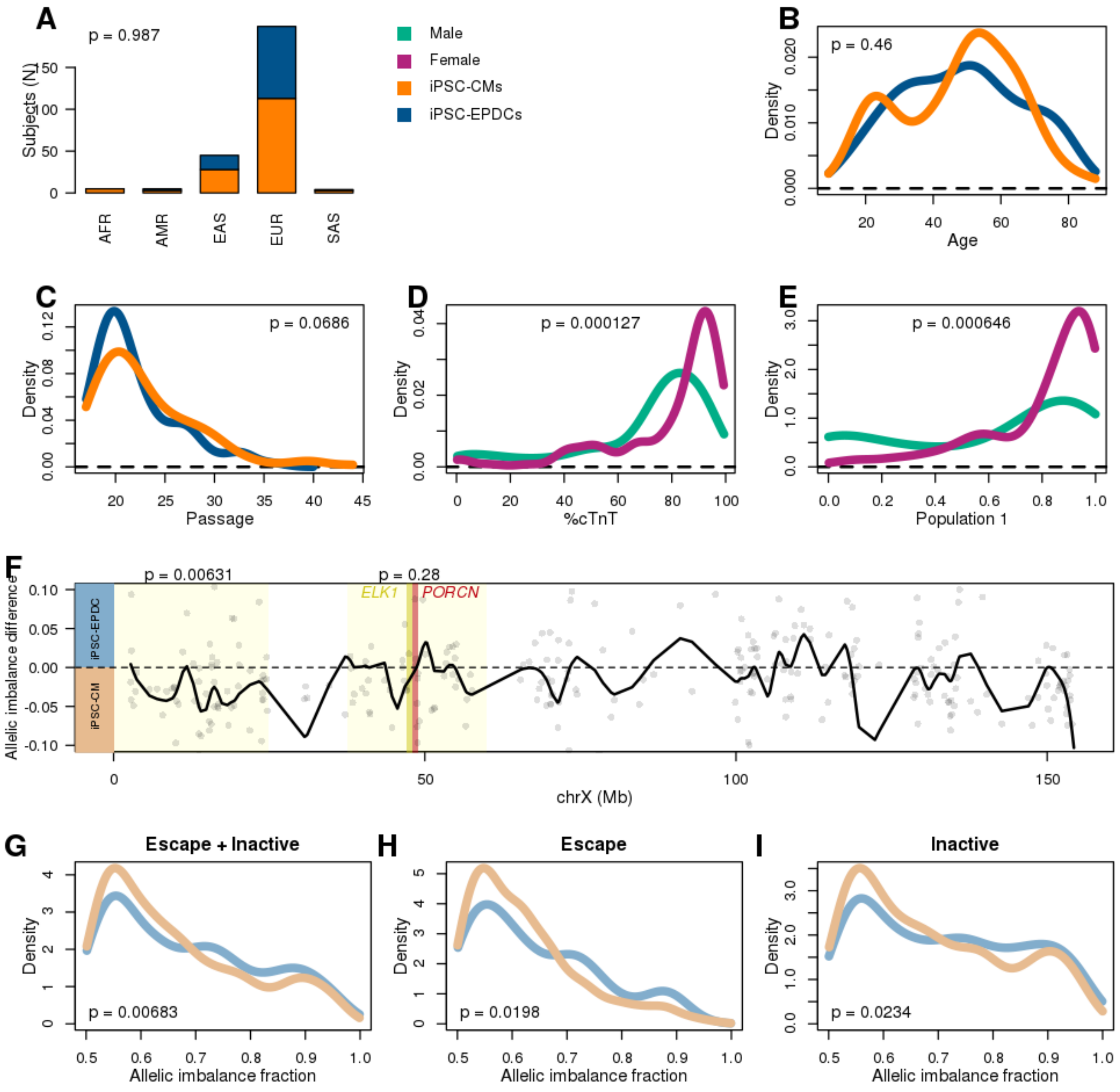

(A-C) Associations between differentiation outcome (orange: iPSC-CVPC samples with CM fraction > 30%; blue: with EPDC fraction > 70%) and (A) ethnicity (most similar superpopulation from the 1000 Genomes Project), (B) age at enrollment, and (C) passage at monolayer (D0). (A) is shown as barplots; (B,C) are shown as density plots. P-values were calculated using Z-test (glm function in R).

(D-E) Density plots showing the association between sex (teal: males; magenta: females) and (D) %cTnT, and (E) fraction of CM population for 191 iPSC lines. P-values were calculated using Mann-Whitney U test.

(F) Allelic imbalance difference between CM-fated and EPDC-fated iPSCs. The dots represent each gene on chrX, while the black solid line corresponds to the smoothed interpolation of differences for all the genes. The locations of the Xp22 and Xp11 loci on the chrX G-banding ideogram are highlighted in yellow, as well as *ELK1* (yellow) and *PORCN* (red). P-values above each locus indicate the difference in allelic imbalance between CM-fated and EPDC-fated iPSCs in each locus (Mann Whitney U test).

(G-I) Allelic imbalance fraction from inactive and escape genes in Xp22: Density plots showing the allelic imbalance differences in chrX genes on the Xp22 loci in female samples between iPSC lines with CM-fate (light blue) and EPDC-fate (light orange) differentiations. Allelic balances compared in Xp22 are from all genes in the region (G), escape genes (H), and inactive genes (I). P-values were calculated using Mann Whitney U test.

Figure S7: Cell populations at 15 time points during iPSC-CM differentiations

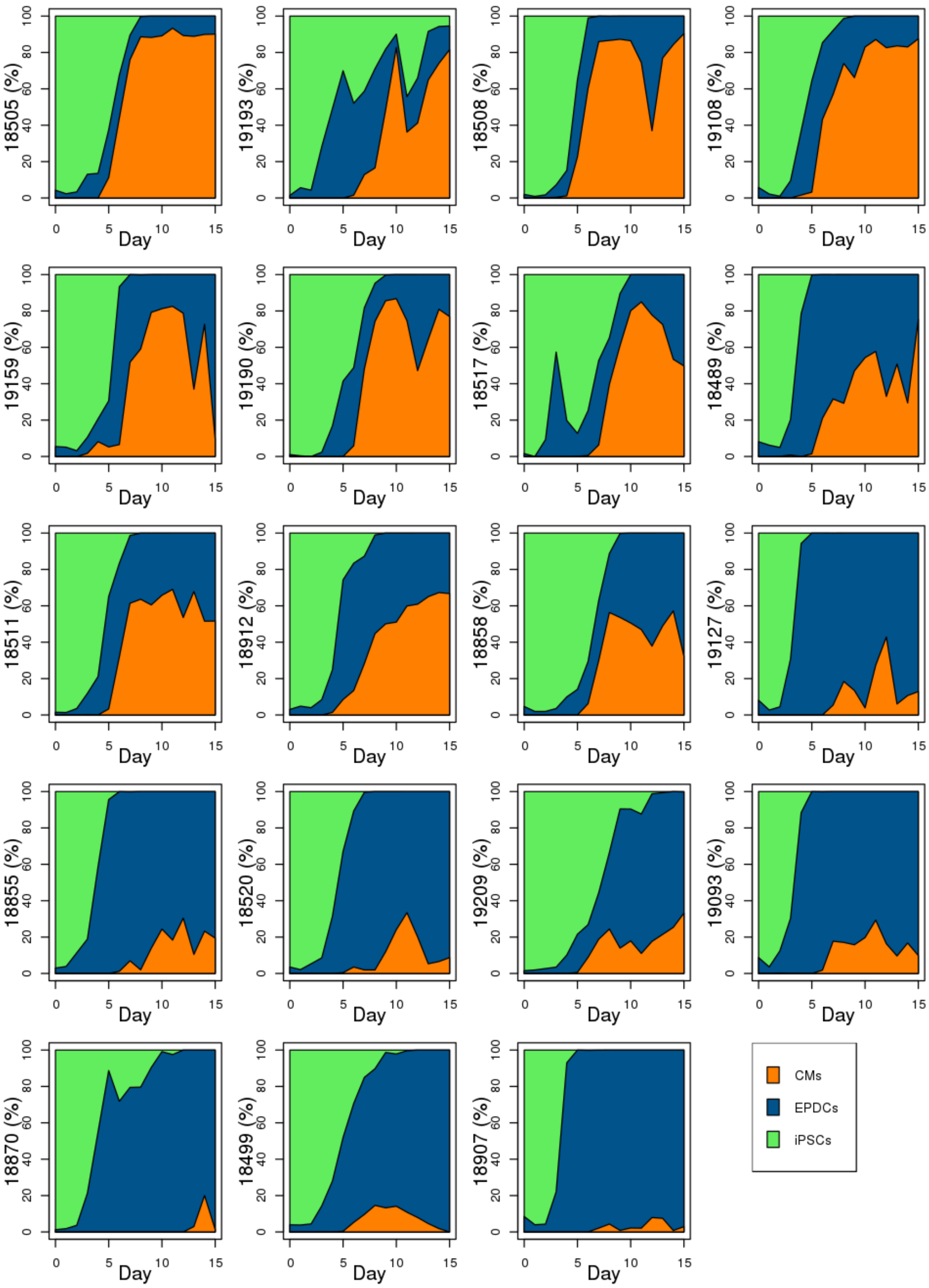

For each of 19 samples in Strober et al. (Strober et al., 2019), at each day during differentiation the relative distributions of iPSCs, CMs, EPDCs are shown. **Related to Figure 5.**

TABLE LEGENDS

Table S1: Characterization of cellular heterogeneity in iPSC-CVPC samples. Related to Figure 1.

Table S1A: Subject information for participants in iPSCORE for which iPSCs were used for iPSC-CVPC differentiation.

iPSCORE_ID indicates family and individual number (e.g. iPSCORE_family#_individual#). Subject_UUID is an assigned Universal Unique Identifier (UUID) for the subject. Family_ID classifies the subject by family to identify related family members. Columns D-G represent the twin and parent information for each subject, as included in dbGaP (phs001325.v1.p1; phs000924.v1.p1) as part of the iPSCORE Resource: Twin_ID_dbgap identifies the dbGaP id if the subject is a twin; Twin_type_dbGap indicates the type of twin (MZ = monozygotic; DZ = dizygotic) if the subject is a twin; Father_subject_ID_dbGap indicates the subject_UUID of the father of the subject if part of the iPSCORE resource; Mother_subject_ID_dbGap indicates the subject_UUID of the mother of the subject if part of the iPSCORE resource. Sex and Age_at_enrollment of the subject are shown. Ethnicities (Self-reported race/ethnicity, Recorded_Ethnicity_Grouping, and Most_similar_1KGP_population) are recorded as described by Panopoulos et al. (Panopoulos et al., 2017). Column M represents cardiac phenotypes.

Table S1B: Table linking identifiers for iPSCORE participants with iPSC-CVPC differentiations and metrics of differentiation outcome.

Unique Differentiation IDentifier (UDID) is a unique digit assigned for each attempted iPSC-CVPC differentiation. iPSCORE_ID indicates family and individual number (e.g. iPSCORE_family#_individual#). Subject_UUID is an assigned Universal Unique IDentifier (UUID) for the subject. iPSC_iPSCORE_ID is the iPSC line identifier submitted to dbGap (phs001325.v1.p1), which indicates clone and passage of iPSC. iPSCORE_resource indicates by TRUE or FALSE if this line is one of the 222 lines described by Panopoulos et al.(Panopoulos et al., 2017). iPSC_ID is the iPSC line identifier. iPSC_passage_at_monolayer (D0) is reported. D_to_D0 describe how many days the iPSC line was cultured to achieve 80% confluency before initiation of differentiation. If the UDID was harvested on D25 (Column I), the harvest density (Column J), number of cryovials frozen (Column K), and measured %cTNT+ by FACS (Column L) is reported. Successful_iPSC_CM_differentiation indicates if the iPSC-CVPC sample was harvested at D25 (e.g. not prematurely terminated). Population_1 indicates the estimated composition of population 1 (cardiomyocyte population) for each sample with RNA-seq (column M, see Table S1E) and the estimated_cell_type (Column N)

indicates iPSC-CVPC samples with ≥30% population 1 as CM and iPSC-CVPC samples with <30% population 1 as EPDC.

Table S1C: Table describing the number of lines and subjects for each attempted differentiation.

For each cell type: iPSC and derived iPSC-CVPCs (both terminated prior to D25 and D25), the number of differentiations performed (Column B) are given. For these differentiations, the number of unique lines used (Column C) from the number of unique subjects used (Column D) is provided.

Table S1D: Antibodies used for FACS and immunofluorescence.

This table describes the antibodies (Column A) and clone (Column B) used for FACS and immunofluorescence experiments. Catalog numbers (Column C), brand (Column D), dilution (Column E), time of staining in minutes (Column F), and temperature of staining (Column G) is indicated for each antibody.

Table S1E. Table linking identifiers for iPSC and iPSC-CVPC genomic data.

UDID is given if the iPSC or iPSC-CVPC genomic data were collected during an attempted differentiation, indicated by UDID (Column A). Subject_UUID (Column B) is an assigned Universal Unique Identifier (UUID) for the subject. Cell (Column C) indicates the stage for which the genomic data was generated (iPSC or iPSC-CVPC). Genomic data UUIDs are given in Columns D-E, including rna_assay_uuid (bulk RNA-seq; Column D), scrna_assay_uuid (scRNA-seq; Column E). Estimated cellular composition from CIBERSORT of populations 1-3 is given in Columns F-H.

Table S1F. Generated molecular data.

For each cell type (iPSC and iPSC-CVPC) (Column A) and for each assay for which molecular data was generated (RNA-seq and scRNA-seq), the number of data samples (Column C), from the number of unique lines (Column D), and from the number of unique subjects (Column E) are given.

Table S1G: scRNA-seq features for each sequenced single cell.

For each of the 34,905 cells with scRNA-seq data, the table shows iPSCORE subject ID (Column A) and UUID (Column B), barcode (Column C), associated population (Column D), and coordinates on the t-SNE plot (Columns E, F). For the H9 ESC line sample, which is not included in iPSCORE, iPSCORE ID and Subject UUID are labeled as “ESCs”. This table is ordered on population (e.g. clusters 1, 2, 3).

Table S1H: Table describing observed beat scores and structure scores for iPSC-CVPC differentiations.

UDID (Column A) for each differentiation measured is given. Beat.Score (Column B) indicates the estimated beat score for the differentiation and Structure.Score indicates the observed structure score for the sample (Column C).

Table S2: Overexpressed genes in each scRNA-seq population. Related to Figure 2.

For each of 34,528 genes (Columns A, B) with at least one transcript detected in the scRNA-seq samples, the mean UMI counts, log2 fold change, and FDR-adjusted p-value is shown for each population. The last column indicates the 150 genes used as input for CIBERSORT.

Table S3: iPSC gene signatures associated with cardiac differentiation fate. Related to Figure 3.

Table S3A-B: Differential expression between iPSCs differentiated to CMs and iPSCs differentiated to EPDCs using multiple thresholds

We used 10 different thresholds to divide iPSCs based on their %CM population detected using CIBERSORT (Figure S7). For each threshold (Columns B-M) (>0%, 10%, 20%, 30%, 40%, 50%, 60%, 70%, 80%, 90%; males and females; EPDC fate vs. Terminated), differential expression between the samples passing and not passing the threshold was calculated using t-test. Table S3A and Table S3B respectively show nominal p-values and Storey q-values. P-values of differentially expressed genes calculated by t-test are shown for all 15,228 human genes (Column A) expressed in iPSC-CVPCs. We examined genes that were differentially expressed in the iPSCs generated from females versus males (column L), and removed 242 genes that were differentially expressed from downstream analyses (Storey q-values < 0.1). Across the ten CM:EPDC ratios there were 116 genes that were differentially expressed at one or more ratio. At the 30% threshold, there were the greatest number of significantly differentially expressed genes. EPDC-fated iPSCs consisted of those with completed iPSC-CVPC differentiations (i.e. reached D25) with >70% Population 2 and iPSCs with differentiations that were terminated before D25. We did not observe significant expression differences between the 22 iPSCs that differentiated to EPDCs (>70% Population 2) and the 37 iPSCs whose differentiations were terminated before D25 (column M). For further analyses, we used the 30:70 (CM:EPDC) threshold, because it maximized the differences between CMs and EPDCs, i.e. the largest number of differentially expressed genes (84).

Table S3C: Differential expression analysis using 30:70 CM:EPDC ratio as threshold.

In this table, we report the differential expression analysis performed between the 125 iPSC samples defined as CM-fated and 59 iPSC samples defined as EPDC-fated (15,228 autosomal genes, labeled as "All iPSC samples" in column H). We also report the differential expression analysis performed between the 87 female iPSC samples defined as CM-fated and the 26 female iPSC samples defined as EPDC-fated (15,398 expressed genes located on both autosomes and on the chromosome X, labeled as "Female samples" in column H). For each gene (Columns A, B), shown are: 1) the mean normalized expression across the CM-fated iPSCs (column C), 2) the mean normalized expression across the EPDC-fated iPSCs (column D), 3) the difference between mean normalized expressions (column E), 4) the p-value (t-test) (Column F), and 5) Storey q-value (Column G). A positive difference between mean normalized expressions indicate CM-fated over-expression, whereas a negative difference between mean normalized expressions indicate EPDC-fated over-expression.

Table S3D: Description and supporting literature of 91 signature genes in iPSC.

In this table, we describe the known functions of the 91 signature genes identified as differentially expressed between CM-fated and EDPC-fated iPSCs. For each gene, gene name (Column A), ensemble gene id (Column B), Chromosome (column C), gene start (Column D) and end (Column E), the difference between mean normalized expressions (column F), the p-value (t-test) (Column G), and Storey q-value (Column H) are given. Additionally, for each gene functional descriptions (Column J) and PMIDs for supporting literature (Column I) are provided. This table is ordered on the difference between mean normalized expressions.

Table S3E: Regression estimates showing the associations between signature genes and %CM populations.

For each of the 91 signature genes (Columns A, B), linear regression estimate (Column C), standard error (Column D), p-values (Column E, calculated in R as 2*pnorm(estimate / standard error)) and R^2 (Column F) are shown. Columns G-J show the 35 signature genes that L1 norm identified as having significant contribution to cell fate determination: LASSO regression coefficient (Column G), median TPM (Column H), median contribution (Column I), and absolute value of the median contribution to the model (Column J) are shown. Column K shows the seven ELK1 targets as identified in the MSigDB gene set SCGGAAGY_ELK1_02 (Xie et al., 2005).

Table S3F: Associations between genetic variation and differentiation outcome.

For each variant with a GWAS p-value > 10^{-5}, shown are their chromosome (Column A), coordinates (Column B), reference and alternative allele (Columns C, D), dbSNP ID (Column E), allele frequency in iPSCORE (Column F), regression estimate (Column G), standard error (Column H) and p-value (Column I). Regression

estimate, standard error and p-value were calculated using the glm(CM population ~ genotype, family = "quasibinomial") function in R.

Table S4: X chromosome gene dosage plays a role in cardiac differentiation fate. Related to Figures 4 & 5.

Table S4A: GSEA showing functional enrichment of genes differentially expressed between CM-fated and EPDC-fated iPSCs

For each of 9,808 MSigDB gene sets (Column A), GSEA enrichment (Column B), and p-value (Column C) calculated using the R gage package are shown. Storey q-value was used to adjust for multiple testing hypothesis, q-values < 0.05 were considered significant. The analysis (Column E) shows whether the test was performed on all 184 iPSCs ("All iPSC-CVPC samples") or just on the 113 female samples ("Female samples"). Positive GSEA enrichment indicate enrichment for CM-fated iPSCs, whereas negative GSEA enrichment indicate enrichment for EPDC-fated iPSCs.

Table S4B: Table describing results of linear regression analysis to predict factors influencing differentiation potential of iPSC towards CM or EPDC fates. Related to Figure 4.

Factors (Column A) input into the linear regression model. Columns B-E describe the results of the model, including estimate (Column B), standard error (Column C), z-value (Column D), and p-value (Column E).

Table S4C: Allelic imbalance fraction of genes on the X chromosome not in pseudoautosomal regions in females from iPSC samples and from iPSC-CVPC samples. Related to Figure 4.

Gene_id indicates the ensemble gene id (Column A) for X chromosomes genes not in pseaudoautomsomal regions. Columns B-HR show the rna_assay_uuid (Table S1E) of each of the female iPSC (Figure 4D,E) and iPSC-CVPC samples (Figure 4F) for which the allelic imbalance fraction was calculated for each gene.

Table S5: Table describing differentiation outcomes and molecular data ID references from the Yoruba set. Related to Figure 5.

Data from 39 Yoruba iPSC samples (Banovich et al., 2018) (Column A) and their sex (Column B) are given. Outcome (Column C) indicates if the iPSC-CM differentiation was completed or terminated before completion. %cTnT values (Column E), GEO iPSC RNA-seq sample IDs (Column F), and GEO iPSC-CM sample IDs

(Column G) (GEO; GSE89895) are given. Five of the Yoruba iPSCs and two of the iPSC-CM samples did not have RNA-seq data.

SUPPLEMENTARY EXPERIMENTAL PROCEDURES

iPSCORE subject information

Fibroblasts obtained by skin biopsies from the181consented individuals (108 female and 73 male) used in this study were recruited as part of the iPSCORE project (Panopoulos et al., 2017). These individuals included seven monozygotic (MZ) twin pairs, members of 32 families (2-10 members/family) and 71 singletons (i.e. not related with any other individual in this study) and were of diverse ancestries: European (118), Asian (27), Hispanic (12), African American (4), Indian (3), Middle Eastern (2) and mix ethnicity (15). The recruitment of these individuals was approved by the Institutional Review Boards of the University of California, San Diego and The Salk Institute (Project no. 110776ZF). Subject descriptions including subject sex, age, family, ethnicity and cardiac diseases were collected during recruitment (Table S1). While individuals in the iPSCORE Resource were not selected for carrying specific diseases, six individuals had prolonged QT (due to dominant mutations in *KCNQ1* or *KCNH2*), and two members of the same family had Danon disease (due to mutations in *LAMP2*). In addition to fibroblast collection for iPSC reprogramming and differentiation, whole blood samples were obtained for whole genome sequencing.

Whole genome sequencing

As previously described (DeBoever et al., 2017), we generated whole genome sequences from the 181 subjects used for iPSC derivation. Genomic DNA was isolated from whole blood using DNEasy Blood & Tissue Kit (Qiagen) and Qubit quantified. DNA was then sheared using Covaris KE220 instrument and normalized to 1µg, where WGS libraries were prepared using TruSeq Nano DNA HT kit (Illumina) and normalized to 2 - 3.5nM in 6-samples pools. Pooled libraries were clustered and sequenced on the HiSeqX (Illumina; 150 base paired-end) at Human Longevity, Inc. (HLI).

iPSC derivation and somatic mutation analysis

As previously described (Panopoulos et al., 2017), we reprogrammed fibroblast samples from the 181 individuals in this study using non-integrative Cytotune Sendai virus (Life Technologies) (Ban et al., 2011) following the manufacturer's protocol. The 191 iPSCs used in this study (7 subjects had 2 or more clones each; Table S1B) were generated and shown to be pluripotent by analysis of RNA-seq by PluriTest (Muller et al., 2008) and for a subset based on >95% positive double staining for Tra-1-81and SEEA-4 (Panopoulos et al., 2017). The iPSCORE lines have been examined using SNP arrays and shown to have high genomic integrity with no or low numbers of somatic copy-number variants (CNVs) (Panopoulos et al., 2017). Eighteen iPSCORE lines have been analyzed using whole genome sequencing and the mutational profiles shown to

be stable (i.e., not evolving) between passage 12 and later passages, and throughout differentiation into iPSC-CVPCs (D'Antonio et al., 2018).

Large-scale derivation of iPSC-CVPC samples

To generate iPSC-derived cardiovascular progenitors (iPSC-CVPCs) we used a small molecule cardiac differentiation protocol (Lian et al., 2013). The 25-day differentiation protocol consisted of five phases (Figure S1A), the optimizations for each step are described in detail below: 1) *expansion*: we developed the ccEstimate algorithm (Figure S2) to automate the detection of 80% confluency for iPSCs in T150 flasks (Figure S1B,C); 2) *differentiation*: we tested whether increasing the dosage of IWP-2 to induce to inhibit the WNT pathway improved differentiation efficiency and found that 7.5 µM at D3 of the differentiation provided in a single dose for 48 hours results in the most efficient differentiation (Figure S1D, E, Table S1H); 3) *purification*: since fetal cardiomyocytes use lactate as primary energy source and have a higher capacity for lactate uptake than other cell types (Fisher et al., 1981; Werner and Sicard, 1987), we incorporated lactate metabolic selection for five days to improve iPSC-CVPC purity (Tohyama et al., 2013) (Figure S1F); 4) *recovery*: after metabolic selection, iPSC-CVPCs were maintained in cell culture for five days; and 5) *harvest*: we collected iPSC-CVPCs at D25 for downstream molecular assays and cryopreserved live cells.

The 232 attempted differentiations of the 191 iPSC lines (Table S1B) were performed as follows:

Expansion of iPSC: One vial of each iPSC line was thawed into mTeSR1 medium containing 10 µM ROCK Inhibitor (Sigma) and plated on one well of a 6-well plate coated overnight with matrigel. During the expansion phase, all iPSC passaging was performed in mTeSR1 medium containing 5 µM ROCK inhibitor, when cells were visually estimated to be at 80% confluency. The iPSCs were passaged using Versene (Lonza) from one well into three wells of a 6-well plate. Next, the iPSCs were passaged using Versene onto three 10 cm dishes at $2.54 x 10^4$ per cm^2 density. The iPSCs molonalyer was plated onto three T150 flasks at the density of 3.66×10^4 per cm^2 using Accutase (Innovative Cell Technologies Inc.). Prior to expansion with Versene, after thaw iPSCs were passaged 1-2 times using Dispase II (20mg/ml; Gibco/Life technologies). iPSCs were at passage 22.7 ± 4.8 (range 17 to 44) at the monolayer stage (i.e., initiation of differentiation; Table S1B).

Differentiation: At 80% iPSC confluency (measured using ccEstimate, see section below "Estimation of optimal time for initiation of iPSC-CVPCs differentiation using ccEstimate") cell lysates were collected from 32 lines for RNA-seq data generation, where these iPSC and subsequent generated molecular data are referred to as D0 iPSC (Table S1E). After reaching 80% confluency (usually within 4-5 days), differentiation was initiated with the addition of the medium containing RPMI 1960 (gibco-life technologies) with Penicillin – Streptomycin (Gibco/Life Technologies) and B-27 Minus Insulin (Gibco/Life Technologies) (hereafter referred to as RPMI Minus supplemented with 12µM CHIR-99021 (D0). After 24h of exposure to CHIR-99021, medium was changed to RPMI Minus (D1). On D3 medium was changed to 1:1 mix of spent and fresh RPMI Minus supplemented with 7.5µM IWP-2 (Tocris). On D5, after 48h of exposure to IWP-2, the medium was change to RPMI Minus. On D7, medium was changed to RPMI 1960 with Penicillin – Streptomycin (Gibco/Life

Technologies) and B-27 Supplement 50X (hereafter referred to as RPMI Plus) (Gibco/Life Technologies). Between D7 and D13, RPMI Plus medium was changed every 48h.

Purification: On D15 the cells were collected from the flask using Accutase and plated onto fresh T150 flasks at confluency 1-1.3 x 10^6 per cm^2. On D16, cells were washed with PBS without Ca^{2+} and Mg^{2+} (Gibco/Life Technologies) and medium was changed for RPMI 1960 no glucose (Gibco/Life Technologies) supplemented with Non-Essential Amino Acids (Gibco/Life Technologies), L-Glutamine (Gibco/Life Technologies), Penicillin-Streptomycin 10,000U (Gibco/Life Technologies) and 4mM Sodium L-Lactate (Sigma) in 1M HEPES (Gibco/Life Technologies). Medium supplemented with lactate was changed on D17 and D19.

Recovery: On D21 cells were washed with PBS and medium was changed for RPMI Plus. On D23 medium was again changed for RPMI Plus. The first beating cells were usually observed between D7 and D9 and as early as D7 (immediately after the media change) and robust beating was usually observed between D8 and D11. During the lactate selection iPSC-CVPC were beating robustly less than 16 hours after reseeding. For all successfully derived iPSC-CVPCs on D25, total-cell lysate material was collected and frozen for downstream RNA-seq assays.

Harvest: On D25 cells were collected using Accutase and processed for the following molecular material for downstream assays: 1) cell lysates (RNA-Seq); 2) permeabilized cells (ATAC-Seq); 3) live frozen cells (scRNA-seq); 4) cross-linked cells (ChIP-Seq, median number of vials/iPSC line = 3; ~1.0 x 10^7 cells/vial), and 5) dry cell pellets (methylation and protein). RNA-seq was generated from 180 iPSC-CVPC differentiations (149 lines from 139 subjects) that successfully reached D25 (Table S1E).

Estimation of optimal time for initiation of iPSC-CVPCs differentiation using ccEstimate

Heterogeneity of growth rates across different iPSC lines could result in different confluency at the monolayer stage (i.e., faster growing lines will be more confluent) and hence impact differentiation outcome. To reduce the effects of the iPSC lines having different growth rates, we developed an automatic pipeline that analyzes images of monolayer-grown cells, determines their confluency and predicts when cells reach 80% confluency to initiate the differentiation protocol (Figures S1C, S2). Cell confluency estimates (ccEstimate) are performed by first dividing each T150 flask into 10 sections (Figure S1C) and acquiring images for each section every 24 hours after cells are plated as a monolayer. The final image is acquired immediately after treatment with CHIR, which occurs when their confluence is at least 80% (Day 0). The time required for cells to reach 80% confluence is estimated on the basis of the confluence curve derived for each section in each flask. To digitally measure iPSC confluency, ccEstimate performs image analysis using the EBImage package in R (Pau et al., 2010). Images are read using the readImage function.

Confluency measurement data is collected for at least the first three days after plating as monolayer to train a generalized linear model (GLM) using the function glm in R to estimate when cells must be treated with CHIR. Estimation is performed

separately for each flask section and CHIR is added to all three flasks associated to a given line when at least 75% of sections have confluence 80% (Figure S1C).

Using this method, we could start differentiation at the same confluency level for each iPSC sample, thereby reducing or neutralizing the effects of different growth rates. On average, each sample required 4.23 ± 1.12 days to reach 80% confluency (Table S1E). The correlation between the number of days required to reach 80% confluency and the %CM population was -0.05, suggesting that iPSC growth rate does not affect differentiation outcome.

Optimization of IWP-2 concentration by visual estimation of iPSC-CVPCs structure and beating quality

To optimize the IWP-2 concentration, one iPSC line (2_3) was differentiated under four different IWP-2 conditions (Figure S1D, E): 1) 5µM IWP-2 added on D3, 2) 7.5µM IWP-2 added on D3, 3) 5µM IWP-2 added on D3 and D4, or 4) 7.5µM IWP-2 added on D3 and D4. In all four conditions cells were exposed to IWP-2 for 48 hours. At D15 of differentiation, the quality of generated iPSC-CVPC structures and beating were estimated by visual evaluation using two metrics that we established in the lab: 1) structure score; and 2) beat score. Both structure score and beat score were evaluated at 10 spots on each 150T flask that had also been used for digital measurement of cell confluency (Table S1H). Structure score and beat score had 4-point scales where 0 was the lowest and 3 was the highest grade. For structure score 0 = less than 10% of cells were cardiomyocyte-like with thick structures; 1 = 10-25% of cells were cardiomyocyte-like with thick structures; 2 = over 50% of cells were cardiomyocyte-like with thick structures; 3 = over 90% of cells were cardiomyocyte-like with thick structures. For beat score 0 = less than 10% of cells were cardiomyocyte-like beating robustly as a sheet; 1= 10-25% of cells were cardiomyocyte-like beating robustly; 2 = over 50 of cells were cardiomyocyte-like beating robustly; 3 = over 90% of cells were cardiomyocyte-like beating robustly. In cases of uncertainty or intermediate results, cells were assigned a lower grade. Grade 3 was assigned only for the iPSCs with thick, robustly beating sheets of cells.

Comparison of lactate and glucose treated iPSC-CVPCs

To examine the effects of lactate purification, three iPSC-CVPC lines derived from unrelated individuals (2_3, 8_2, and 3_2) were differentiated to D15 (Figure S1F). At D16, medium supplemented with either 4mM Sodium L-Lactate (Sigma) or 2mg/mL D-glucose (Gibco/Life Technologies). Medium was changed on D17 and D19. On D21 cells were washed with PBS and medium was changed for RPMI Plus. Lactate and glucose treated cells were harvested on D25.

Flow cytometry

On D25 of differentiation, $5x10^5$iPSC-CVPCs were permeabilized and blocked in 0.5% BSA, 0.2% TX-100 and 5% goat serum in PBS for 30 minutes at room temperature. Cells were stained with Troponin T, Cardiac Isoform Ab-1, Mouse Monoclonal Antibody (Thermo Scientific, MS-295-P0) at 4°C for 45 minutes, followed by Alexa Fluor 488 secondary antibody (Life Technologies, A11001). Stained cells were acquired using BD FACSCanto II system (BD Biosciences) and analyzed using FlowJo V10.2.

Immunofluorescence analysis of iPSC-CVPCs

Immunofluorescence (IF) was assessed in 5 iPSC-CVPC lines (13_1, 14_2, 29_1, 2_1, and 42_1). Cells for IF were obtained by thawing live frozen iPSC-CVPC harvested on D25 and plating them directly on 0.1% gelatin-coated glass-bottom plates for five days (D30). Cells were then fixed using 4% paraformaldehyde (PFA) in PBS or 20 min at room temperature (RT). Fixed cells were permeabilized for 8 min at RT with 0.1% Triton X-100 in PBS, blocked in 5% bovine serum albumin for 30 min at RT and incubated overnight at 4°C with a primary antibody. Cells were incubated with rabbit polyclonal anti-connexin 43 (Cx43) antibody (Invitrogen, 710700) and with mouse monoclonal anti-sarcomeric alpha-actinin antibody (Sigma, A7811), or with rabbit polyclonal anti-MLC2V (Proteintech, 10906-1-AP) and/or mouse monoclonal anti-MLC2A (Synaptic Systems, 311011). All antibodies are described in Table S1D.

After overnight incubation cells were washed three times with PBS and incubated with appropriate secondary antibodies: donkey anti-rabbit Alexa Fluor 488(Invitrogen, A-21206) and goat anti-mouse Alexa Fluor 568 (Invitrogen, A-11004**)** secondary antibodies for 45 minutes at RT. Cells were washed three times with PBS and nuclei were counterstained with DAPI and mounted. Slides were imaged using Olympus FluoView FV1000 confocal microscope at UCSD Microscopy Core.

Generation of RNA-seq data

For gene expression profiling of iPSCs, we used RNA-seq data from 184 samples (cell lysates were collected between passages 12 to 40, Table S1, dbGaP: phs000924) (DeBoever et al., 2017). For gene expression profiling of iPSC-CVPCs, we generated RNA-seq data from 180 samples at D25 differentiation (Table S1F, dbGaP: phs000924). All RNA-seq samples were generated and analyzed using the same pipeline (DeBoever et al., 2017). Briefly, we isolated total RNA from total-cell lysates using the Quick-RNA™ MiniPrep Kit (Zymo Research) from frozen total-cell lysate, including on-column DNAse treatment steps and eluted in 48 µl RNAse-free water. RNA elutions were run on a Bioanalyzer (Agilent) to determine integrity and all samples had RNA integrity number (RIN) values greater than 9. Illumina Truseq Stranded mRNA libraries were prepared and sequenced on HiSeq4000, to an average of 28 M 125 bp paired-end reads per sample. RNA-Seq reads were aligned using STAR (Dobin et al., 2013) with a splice junction database built from the Gencode v19 gene annotation. RNA-Seq data with percent uniquely mapped reads greater than 70% and percent duplication less than 50% were considered to be good quality. Transcript and gene-based expression values were quantified using the RSEM package (1.2.20) (Li and Dewey, 2011) and normalized to transcript per million bp (TPM).

Generation of scRNA-seq data

Rationale: To capture the full spectrum of heterogeneity among the iPSC-CVPCs, we selected eight samples with variable %cTnT (42.2 to 95.8%). Given the high correlation that we observed between %cTnT and %CM populations in these eight samples, as well as the high correlation between %cTnT and deconvoluted %CM population across all samples with bulk

RNA-seq, we concluded that eight samples were sufficient to capture the full diversity of heterogeneity among the 191 iPSC lines that were differentiated.

Generation: For eight iPSC-CVPCs sample and one H9 ESC line, single cells were captured using the 10x Chromium controller (10x Genomics) according to the manufacturer's specifications and manual (Manual CG00052, Rev C). Cells for each sample were loaded on the individual lane of a Chromium Single Cell A Chip. Libraries were generated using Chromium Single Cell 3' Library Gel Bead Kit v2 (10xGenomics) following manufactures manual. Libraries were sequenced using a custom program (26-8-98 Pair End) on HiSeq 4000. Each library was sequenced on an individual lane. In total we captured 36,839 cells. We retrieved FASTQ files and used CellRanger V2.1 (https://support.10xgenomics.com/) with default parameters using Gencode V19 gene annotation to generate single-cell gene counts for each individual sample.

Processing: To combine the scRNA-seq from each individual sample, we used *cellranger aggr* and obtained a total of 36,839 cells from 8 iPSC-CVPCs and 1 ESC sample. We removed 1,934 cells because they were not in G0 phase, as they expressed the proliferation marker MKI67 (Scholzen and Gerdes, 2000) at high levels (UMI > 2, Figure S4A-D). We also removed doublets (i.e. sequenced droplets containing more than one cell)(Kang et al., 2018)by visual inspection of the t-SNE plots (Figure S4). There were 34,905 cells remaining after proliferating cells and doublets were removed. K-means clustering was performed on the 34,905 cells using k values 3, 4, and 9 (FigureS4E-G). k = 3 was determined to be the most suitable value, as visual inspection of the principal component analysis showed 3 distinct clusters (Figure S4E). The clustering shown both in the heatmap and in the UMAP plots (Figure 1G, 1H, 1J) was performed on the top 10 principal components calculated based on the expression levels of each single cell, according to the CellRanger pipeline.

Differential expression: Differential expression across the three scRNA-seq clusters was performed by comparing the distribution of unique molecular identifiers (UMI) for a given gene from all the cells specific to one cluster (k-means; k = 3) with all the cells specific to the other two clusters using edgeR asymptotic beta test (Robinson and Smyth, 2008) (Table S2). Differentially genes that had a total UMI ≥ 1 and FDR < 0.05 were considered to be significantly overexpressed in a given cluster. For visualization of gene expression in the t-SNE plots, transcript levels for each gene were normalized using the *calcNormFactors* function in edgeR (Robinson et al., 2010).

CIBERSORT

The expression levels of the top 50 genes overexpressed in each of the three cell populations (total 150 genes), with nominal p-value < 1.0×10^{-13} and mean UMI > 1 (Table S1G), were used as input for CIBERSORT (Newman et al., 2015) to calculate the relative distribution of the three cell populations for all the 180 iPSC-CVPC samples at D25. CIBERSORT (https://cibersort.stanford.edu/) was run with default parameters using the TPM values for the 150 genes in all 180 iPSC-CVPC samples.

Characterizing transcriptional similarities of iPSCs, iPSC-CVPCs and GTEx adult tissues by principal component analysis

We performed principal component analysis (PCA) on RNA-seq using R prcomp function on 184 iPSCs, 180 iPSC-CVPCs and 1,072 RNA-seq samples from GTEx, including 303 left ventricle samples, 297 atrial appendage samples, 173 coronary artery samples and 299 aorta samples.

Determining optimal CM:EPDC ratio estimates from CIBERSORT to define iPSCs cardiac fates

For each iPSC line that had more than one iPSC-CVPC differentiation, we used the sample with the highest Population 1 fraction. To obtain the optimal threshold, we used the RNA-seq data to conduct a series of differential expression analyses on 15,228 autosomal genes in the 184 iPSC lines (147 completed and 37 terminated) with RNA-seq data considering the ratio of population frequencies in the corresponding derived iPSC-CVPCs (0:100, 10:90, 20:80, 30:70, 40:60, 50:50, 60:40, 70:30, 80:20 and 90:10, Table S3A-B). For example, for the 90:10 ratio we compared gene expression in the 60 iPSCs that differentiated into iPSC-CVPCs with >= 90% Population 1 to 124 iPSCs that differentiated into iPSC-CVPCs with less than 90% Population 1. iPSC lines that that had terminated differentiations were assigned a CM:EPDC ratio of 0:100. To calculate differential expression at each threshold between CM-fated iPSCs and EPDC-fated iPSCs, we first retained all genes with TPM ≥ 2 in at least 10 samples and then transformed the RNA-seq TPM data to standard normal distributions by quantile normalization using the function normalize.quantiles from R package preprocessCore (Bolstad et al., 2003). Quantile normalized expression levels were then corrected for the first 10 factors calculated by PEER (Stegle et al., 2012). To remove any biases resulting from the fact that the ratio of male to female iPSCs was 71:113, we identified 242 genes that were significantly differentially expressed between female and male iPSCs (Storey q-value < 0.1, t-test) and removed them from further analyses (Table S3A-B). Across the ten thresholds, there were 116 differentially expressed autosomal genes (t-test, Table S3A-B). Considering these 116 genes, the 30:70 (CM:EPDC) ratio resulted in the highest number of differentially expressed genes (84 genes with Storey q-value < 0.1, t-test, Figure 3, Table S3C), which is substantially greater than random expectation (Table S9). Thus, we grouped the 184 iPSC lines into: 1) those that have CM fates, i.e. produced iPSC-CVPC with >= 30% Population 1 (125 lines), and 2) those that have EPDC fates, i.e. produced iPSC-CVPC with > 70% Population 2 (22 lines differentiated to D25 and 37 terminated lines). We did not observe significant expression differences between the 22 iPSCs that were designated EPDC-fated because their corresponding iPSC-CVPCs had >70% Population 2 and the 37 iPSCs designated EPDC-fated because their differentiations were terminated before D25 (Table S3A-B).

Comparing the number of differentially expressed genes with random expectation

To determine if the number of significantly differentially expressed genes was higher than expected by chance, we shuffled the assignments of the 184 iPSC RNA-seq samples to differentiation fate (125 CM and 59 EPDC) 100 times. For each shuffle, we performed differential expression analysis and obtained the number of genes that were significantly differentially

expressed. In Figure S9 we show a QQ plot that demonstrates that the observed p-value distribution was substantially different than random expectation.

Contribution of 91 signature genes in iPSCs to determination of cardiac fate

Individual contributions: For each of the 91 signature genes, we built a generalized linear model (GLM) with the expression of the gene as input and the differentiation outcome (e.g. % Population 1) as output using the LinearRegression function from sklearn. To model the continuous property of the % Population 1 distributions, but maintain their boundary from 0-100, we used a logit link function to transform measurements of % cardiomyocyte to ln(OR) of % Population 1, calculated as ln(% Population 1/ (1 - % Population 1) and capped the percentages at 0.99 and 0.01 to avoid infinite or undefined odds ratios. For each gene, the percent of variance explained is defined as the model's R^2.

Cumulative impact: To understand the cumulative contribution of all 91 signature genes on cardiac differentiation fate, we built a generalized linear model (GLM) with an L1 norm penalty (ie LASSO) using the expression of all 91 genes as input and the differentiation outcome (e.g. % Population 1) as output using the LassoLarsCV function from sci-it learn. v0.19.1 To model the continuous property of the % Population 1 distributions, but maintain their boundary from 0-100, we used a logit link function to transform measurements of % cardiomyocyte to ln(OR) of % CM population, calculated as ln(% CM/ (1 - % CM) and capped the percentages at 0.99 and 0.01 to avoid infinite or undefined odds ratios. To avoid overfitting the model, we used a 10-fold cross validation implemented in sci-kit learn v0.19.1 with 10,000 max iterations (Pedregosa et al., 2011). The average R^2, as reported by sci-it learn, is calculated by finding the R^2 for each of the individual folds (i.e., 10 R^2s), and averaging these values to find how well the model performs across different data subsets.

Detecting associations between genetic background and differentiation outcome

We obtained genotypes for 8,620,159 biallelic SNPs and short indels with allelic frequency >5% in the iPSCORE collection (Panopoulos et al., 2017). Genotypes were obtained for each SNP in all individuals using *bcftools view* (Li, 2011). Linear regression was used to calculate the associations between the genotype of each variant and differentiation outcome (% CM population in the iPSC-CVPCs), using passage at monolayer and sex as covariates.

To test if differentiations of different iPSC clones from the same individual or same twin pair were more likely to produce similar outcomes than iPSC clones from individuals with different genetic backgrounds, we first calculated the absolute difference in %CM between each pair of 180 iPSC-CVPCs. Next, we tested if the distributions between the three groups were different using Mann-Whitney U test (Figure S11B).

Gene set enrichment analysis using the MSigDB collection

We performed gene set enrichment analysis (GSEA) using the R *gage* package (V 2.20.1)(Luo et al., 2009) on all MSigDB gene sets (Liberzon et al., 2011; Subramanian et al., 2005) from 8 collections, including Hallmark gene sets (H), positional

gene sets (C1), curated gene sets (C2), motif gene sets (C3), computational gene sets (C4), Gene Ontology (GO, C5), oncogenic signatures (C6), and immunologic signatures (C7). FDR correction was performed independently for each collection. The normalized mean expression difference between iPSCs that differentiated to CMs and iPSCs that differentiated to EPDCs (Table S3C) was used as input for GSEA. Gene lists that were significant after multiple testing correction (Storey q-value < 0.05) were considered significant.

Associations between iPSC and subject features and differentiation outcome

A generalized linear model (GLM) was built in R using age, sex, ethnicity, age, and passage of the iPSCs at D0 of differentiation as input and differentiation outcome as output (0 = EPDCs; and 1 = CMs). The model was built using the function glm (outcome ~ age + sex + ethnicity + passage, family=binomial(link='logit')).

Identifying X chromosome inactivation in female iPSCs and iPSC-CVPCs

To analyze X chromosome inactivation, we used 113 female iPSCs, of which 87 where CM-fated and 26 were EPDC-fated. To call allele specific effects (ASE) in RNA-Seq from iPSC and iPSC-CVPCs, we used the method previously described in DeBoever *et al*.(DeBoever et al., 2017). Genes lying in X chromosome pseudoautosomal (PAR) regions (PAR1: 60001-2699520, PAR2: 154931044 – 155260560) were removed from the analysis. We defined the strength of ASE for each gene as the fraction of RNA transcripts that were estimated to originate from the allele with higher expression (referred to as allelic imbalance fraction, AIF).

Validation of findings in Yoruba iPSC set

Generation of iPSCs: The Yoruba iPSCs in the Banovich*et al.* study (Banovich et al., 2018) were generated from lymphoblastoid cell lines (LCLs) using an episomal reprogramming strategy. Briefly, this included transfecting LCLs with the episomal plasmids and then culturing for seven days in hESC media (DMEM/F12 supplemented with 20% KOSR, 0.1 mM NEAA, 2mM GlutaMAX, 1% Pen/Strep, 0.1# 2-Mercaptoethanol, 25ng/µl of bFGF, and 0.5mM NaB). On day eight, the transfected cells were plated in a 6-well plates. After four days, NaB was removed from the hESC media. Colonies were observed within 21 days and passaging continued for an additional 10 weeks (1 passage / week), where cells were collected for cryopreservation. Material collected for RNA-seq of the iPSC were collected after an additional minimum of three passages.

Differentiation protocol: The Yoruba iPSC-CM derivation (Banovich et al., 2018) was performed using a small molecular method similar to iPSCORE iPSC differentiation protocol (see above: Large-scale iPSC-CVPC deviation). Briefly, 39 iPSCs were expanded until 70-100% confluency (three to five days). On D0, differentiation was initiated by the supplementation of media with 12µM of GSK3 inhibitor CHIR-99021 for WNT pathway activation. On D3 of differentiation, 2µM of Wnt-C59 was added (PORCN inhibitor). On D5 of differentiation, Wnt-C59 was removed from culturing media and differentiating cells were grown with regular media exchanges from D5 to D14. On D14, D16, and D18

cultures were exposed to 5mM Sodium L-lactate for cardiomyocyte purification. On D20-D25, differentiating cells were exposed to 1.7 mg/mL galactose daily to force aerobic metabolism and thus aid in cardiomyocyte maturation. On D25-D27, cells were incubated at physiological oxygen levels (10%). On D27 cells were electrically stimulated with 6.6 V/cm, 2ms and 1Hz for further aid in cardiomyocyte maturation. Finally, iPSC-CMs were harvested on D31 or D32. Purity of iPSC-CM Yoruba lines were measured by cTnT marker and flow cytometry. Out of the 39 iPSCs for which differentiation was attempted, 15 lines successfully generated iPSC-CMs and 24 were terminated on or before day 10 due to the fact that they did not form a beating syncytium (Table S5).

RNA-seq: We downloaded RNA-seq for 34 of the Yoruba iPSC (14 successful iPSC and 20 terminated iPSC, five iPSCs did not have RNA-seq) and 13 iPSC-CM samples (two iPSC-CMs did not have RNA-seq) from Gene Expression Omnibus (GEO; GSE89895) (Banovich et al., 2018), as well as 297 samples from 19 distinct iPSCs in a timecourse experiment (day 0-15) performed on the same Yoruba iPSC samples (Strober et al., 2019). These Yoruba RNA-seq data were generated from Illumina TrueSeq prepared libraries and sequenced at 50 bp single-end reads on an Illumina 2500. As iPSCORE RNA-seq was 125 bp paired-end reads, for comparative analyses, we trimmed all iPSCORE iPSC and iPSC-CM data to 50 bp and treated the paired-end reads as single-end reads. Both iPSCORE and Yoruba 50 bp RNA-seq was then processed as described above (Methods: Generation of RNA-seq data). Briefly, RNA-seq was aligned using STAR, then gene expression was quantified using the RSEM package and normalized to TPM.

Estimation of cellular composition: The RNA-seq for the 13 Yoruba iPSC-CMs and from all timecourse time points were analyzed using CIBERSORT similar to the iPSCORE samples (see CIBERSORT section above). Briefly, the TPM values of the 150 overexpressed genes (50 from each of the three single cell populations; Table S2) were used as input to CIBERSORT to calculate the relative distribution of the three populations.

Testing if iPSCORE differentially expressed genes with nominal significant expression differences in the same direction (e.g. over-expressed or down regulated) in the Yoruba iPSCs is greater than random expectation: Of 13,704 genes expressed both in the iPSCORE and Yoruba iPSCs, we obtained 6,909 for which the average normalized expression differences had either the same positive (CM fate/successful differentiation) or negative (EPDC fate/terminated differentiation) direction. The 6,909 genes included 47 of the 91 iPSCORE signature genes. We found that 466 (6.7%) of the 6,909 genes were nominally significant for being differentially expressed between the 14 successful and 20 terminated differentiations in the Yoruba samples, while 8 of the 47 iPSCORE differentially expressed genes (17.0%) had a nominal $p < 0.05$. This analysis shows that the 91 iPSCORE signature genes are 2.5 times more likely than expected (17.0% vs. 6.7%, $p = 0.012$, Fisher's exact test) to be differentially expressed in the Yoruba samples based on cardiac differentiation fate.

REFERENCES

Ban, H., Nishishita, N., Fusaki, N., Tabata, T., Saeki, K., Shikamura, M., Takada, N., Inoue, M., Hasegawa, M., Kawamata, S., *et al.* (2011). Efficient generation of transgene-free human induced pluripotent stem cells (iPSCs) by temperature-sensitive Sendai virus vectors. Proc Natl Acad Sci U S A *108*, 14234-14239.

Banovich, N.E., Li, Y.I., Raj, A., Ward, M.C., Greenside, P., Calderon, D., Tung, P.Y., Burnett, J.E., Myrthil, M., Thomas, S.M., *et al.* (2018). Impact of regulatory variation across human iPSCs and differentiated cells. Genome Res *28*, 122-131.

Bolstad, B.M., Irizarry, R.A., Astrand, M., and Speed, T.P. (2003). A comparison of normalization methods for high density oligonucleotide array data based on variance and bias. Bioinformatics *19*, 185-193.

Burridge, P.W., Matsa, E., Shukla, P., Lin, Z.C., Churko, J.M., Ebert, A.D., Lan, F., Diecke, S., Huber, B., Mordwinkin, N.M., *et al.* (2014). Chemically defined generation of human cardiomyocytes. Nat Methods *11*, 855-860.

D'Antonio, M., Benaglio, P., Jakubosky, D., Greenwald, W.W., Matsui, H., Donovan, M.K.R., Li, H., Smith, E.N., D'Antonio-Chronowska, A., and Frazer, K.A. (2018). Insights into the Mutational Burden of Human Induced Pluripotent Stem Cells from an Integrative Multi-Omics Approach. Cell Rep *24*, 883-894.

DeBoever, C., Li, H., Jakubosky, D., Benaglio, P., Reyna, J., Olson, K.M., Huang, H., Biggs, W., Sandoval, E., D'Antonio, M., *et al.* (2017). Large-Scale Profiling Reveals the Influence of Genetic Variation on Gene Expression in Human Induced Pluripotent Stem Cells. Cell Stem Cell *20*, 533-546 e537.

Dobin, A., Davis, C.A., Schlesinger, F., Drenkow, J., Zaleski, C., Jha, S., Batut, P., Chaisson, M., and Gingeras, T.R. (2013). STAR: ultrafast universal RNA-seq aligner. Bioinformatics *29*, 15-21.

Fisher, D.J., Heymann, M.A., and Rudolph, A.M. (1981). Myocardial consumption of oxygen and carbohydrates in newborn sheep. Pediatr Res *15*, 843-846.

Kadari, A., Mekala, S., Wagner, N., Malan, D., Koth, J., Doll, K., Stappert, L., Eckert, D., Peitz, M., Matthes, J., *et al.* (2015). Robust Generation of Cardiomyocytes from Human iPS Cells Requires Precise Modulation of BMP and WNT Signaling. Stem Cell Rev *11*, 560-569.

Kang, H.M., Subramaniam, M., Targ, S., Nguyen, M., Maliskova, L., McCarthy, E., Wan, E., Wong, S., Byrnes, L., Lanata, C.M., *et al.* (2018). Multiplexed droplet single-cell RNA-sequencing using natural genetic variation. Nat Biotechnol *36*, 89-94.

Li, B., and Dewey, C.N. (2011). RSEM: accurate transcript quantification from RNA-Seq data with or without a reference genome. BMC Bioinformatics *12*, 323.

Li, H. (2011). A statistical framework for SNP calling, mutation discovery, association mapping and population genetical parameter estimation from sequencing data. Bioinformatics *27*, 2987-2993.

Lian, X., Zhang, J., Azarin, S.M., Zhu, K., Hazeltine, L.B., Bao, X., Hsiao, C., Kamp, T.J., and Palecek, S.P. (2013). Directed cardiomyocyte differentiation from human pluripotent stem cells by modulating Wnt/beta-catenin signaling under fully defined conditions. Nat Protoc *8*, 162-175.

Liberzon, A., Subramanian, A., Pinchback, R., Thorvaldsdottir, H., Tamayo, P., and Mesirov, J.P. (2011). Molecular signatures database (MSigDB) 3.0. Bioinformatics *27*, 1739-1740.

Luo, W., Friedman, M.S., Shedden, K., Hankenson, K.D., and Woolf, P.J. (2009). GAGE: generally applicable gene set enrichment for pathway analysis. BMC Bioinformatics *10*, 161.

Muller, F.J., Brandl, B., and Loring, J.F. (2008). Assessment of human pluripotent stem cells with PluriTest. In StemBook (Cambridge (MA)).

Newman, A.M., Liu, C.L., Green, M.R., Gentles, A.J., Feng, W., Xu, Y., Hoang, C.D., Diehn, M., and Alizadeh, A.A. (2015). Robust enumeration of cell subsets from tissue expression profiles. Nat Methods *12*, 453-457.

Panopoulos, A.D., D'Antonio, M., Benaglio, P., Williams, R., Hashem, S.I., Schuldt, B.M., DeBoever, C., Arias, A.D., Garcia, M., Nelson, B.C., *et al.* (2017). iPSCORE: A Resource of 222 iPSC Lines Enabling Functional Characterization of Genetic Variation across a Variety of Cell Types. Stem Cell Reports *8*, 1086-1100.

Pau, G., Fuchs, F., Sklyar, O., Boutros, M., and Huber, W. (2010). EBImage--an R package for image processing with applications to cellular phenotypes. Bioinformatics *26*, 979-981.

Pedregosa, F., Varoquaux, G., Gramfort, A., Michel, V., Thirion, B., Grisel, O., Blondel, M., Prettenhofer, P., Weiss, R., Dubourg, V., *et al.* (2011). Scikit-learn: Machine Learning in Python. J Mach Learn Res *12,* 2825–2830.

Robinson, M.D., McCarthy, D.J., and Smyth, G.K. (2010). edgeR: a Bioconductor package for differential expression analysis of digital gene expression data. Bioinformatics *26*, 139-140.

Robinson, M.D., and Smyth, G.K. (2008). Small-sample estimation of negative binomial dispersion, with applications to SAGE data. Biostatistics *9*, 321-332.

Scholzen, T., and Gerdes, J. (2000). The Ki-67 protein: from the known and the unknown. J Cell Physiol *182*, 311-322.
Stegle, O., Parts, L., Piipari, M., Winn, J., and Durbin, R. (2012). Using probabilistic estimation of expression residuals (PEER) to obtain increased power and interpretability of gene expression analyses. Nat Protoc *7*, 500-507.
Strober, B.J., Elorbany, R., Rhodes, K., Krishnan, N., Tayeb, K., Battle, A., and Gilad, Y. (2019). Dynamic genetic regulation of gene expression during cellular differentiation. Science *364*, 1287-1290.
Subramanian, A., Tamayo, P., Mootha, V.K., Mukherjee, S., Ebert, B.L., Gillette, M.A., Paulovich, A., Pomeroy, S.L., Golub, T.R., Lander, E.S., *et al.* (2005). Gene set enrichment analysis: a knowledge-based approach for interpreting genome-wide expression profiles. Proc Natl Acad Sci U S A *102*, 15545-15550.
Tohyama, S., Hattori, F., Sano, M., Hishiki, T., Nagahata, Y., Matsuura, T., Hashimoto, H., Suzuki, T., Yamashita, H., Satoh, Y., *et al.* (2013). Distinct metabolic flow enables large-scale purification of mouse and human pluripotent stem cell-derived cardiomyocytes. Cell Stem Cell *12*, 127-137.
Werner, J.C., and Sicard, R.E. (1987). Lactate metabolism of isolated, perfused fetal, and newborn pig hearts. Pediatr Res *22*, 552-556.
Xie, X., Lu, J., Kulbokas, E.J., Golub, T.R., Mootha, V., Lindblad-Toh, K., Lander, E.S., and Kellis, M. (2005). Systematic discovery of regulatory motifs in human promoters and 3' UTRs by comparison of several mammals. Nature *434*, 338-345.
